# Biomarkers of sepsis: time for a reappraisal

**DOI:** 10.1186/s13054-020-02993-5

**Published:** 2020-06-05

**Authors:** Charalampos Pierrakos, Dimitrios Velissaris, Max Bisdorff, John C. Marshall, Jean-Louis Vincent

**Affiliations:** 1 Intensive Care Department, Brugmann University Hospital, Université Libre de Bruxelles, Brussels, Belgium; 2 Internal Medicine Department, University Hospital of Patras, Patras, Greece; 3 Department of Intensive Care, Erasme Hospital, Université Libre de Bruxelles, Route de Lennik 808, 1070 Brussels, Belgium; 4 Surgery/Critical Care Medicine, St. Michael’s Hospital, Toronto, Ontario Canada

**Keywords:** Procalcitonin, C-reactive protein, Diagnosis, Prognosis, Infection, Validation

## Abstract

**Introduction:**

Sepsis biomarkers can have important diagnostic, therapeutic, and prognostic functions. In a previous review, we identified 3370 references reporting on 178 different biomarkers related to sepsis. In the present review, we evaluate the progress in the research of sepsis biomarkers.

**Methods:**

Using the same methodology as in our previous review, we searched the PubMed database from 2009 until September 2019 using the terms “Biomarker” AND “Sepsis.” There were no restrictions by age or language, and all studies, clinical and experimental, were included.

**Results:**

We retrieved a total of 5367 new references since our previous review. We identified 258 biomarkers, 80 of which were new compared to our previous list. The majority of biomarkers have been evaluated in fewer than 5 studies, with 81 (31%) being assessed in just a single study. Apart from studies of C-reactive protein (CRP) or procalcitonin (PCT), only 26 biomarkers have been assessed in clinical studies with more than 300 participants. Forty biomarkers have been compared to PCT and/or CRP for their diagnostic value; 9 were shown to have a better diagnostic value for sepsis than either or both of these biomarkers. Forty-four biomarkers have been evaluated for a role in answering a specific clinical question rather than for their general diagnostic or prognostic properties in sepsis.

**Conclusions:**

The number of biomarkers being identified is still increasing although at a slower rate than in the past. Most of the biomarkers have not been well-studied; in particular, the clinical role of these biomarkers needs to be better evaluated.

## Introduction

Biomarkers have been evaluated for several applications in patients with sepsis including diagnosis of infection, prognostication, and therapeutic guidance. Sepsis is a common and severe condition [1, 2], responsible for high mortality and morbidity rates and also for reduced quality of life [1–4]. Sepsis biomarkers may provide information beyond what is available using other metrics and could therefore help inform clinical decision-making and potentially improve patient management. For example, more timely and appropriate antibiotic therapy could be administered and unnecessary antibiotics avoided if biomarkers were available that could accurately diagnose sepsis early. Similarly, biomarkers could help physicians monitor the effectiveness of therapeutic decisions and adjust treatment if necessary [5]. Many potential sepsis biomarkers have been proposed, procalcitonin (PCT) and C-reactive protein (CRP) being the most frequently studied. The Surviving Sepsis Campaign guidelines for the management of sepsis mention that sepsis biomarkers can complement clinical evaluation [6], but in the Sepsis-3 definition consensus, the role of biomarkers in sepsis diagnosis remains undefined [7].

In 2010, we published a literature review of biomarkers that had been studied for their potential diagnostic or prognostic role in sepsis [8]. We concluded that none of the 178 biomarkers identified had “sufficient specificity or sensitivity to be routinely employed in clinical practice” [8]. In this narrative review, we evaluate the progress that has been made in identifying new sepsis biomarkers since that report and reappraise the utility of such research in the management of patients with sepsis.

## Methods

We searched the Medline database via the PubMed portal between February 2009 and September 2019 using (“biomarker” AND “sepsis”) as keywords to identify all studies that evaluated a biomarker in sepsis. There were no restrictions by age or language, and all studies, clinical and experimental, were included. The reference lists from all relevant retrieved manuscripts were further reviewed in order to identify additional studies. For each identified biomarker, the PubMed database was searched again using the biomarker name and the keyword “biomarker.”

Newly found biomarkers were added to our previous database. Details related to the methodology used in each study were collected, namely (1) type of study (mono- vs. multicenter, prospective vs. retrospective, experimental vs. clinical), (2) study population (intensive care unit [ICU], emergency room, other population), (3) number of studied subjects, (4) reference non-sepsis population, and (5) purpose of study or use of biomarker being tested (diagnostic, prognostic, other clinical roles). Results of receiver operating characteristic (ROC) curve analysis were noted where this technique was used to assess biomarker specificity and sensitivity. The Quality Assessment of Diagnostic Accuracy Studies-2 (QUADAS-2) tool [9] was used to assess the methodological quality of the studies that included more than 300 patients and performed ROC analysis. For each biomarker, the main pathophysiological role (Additional file 1, Figure S1) was recorded. We also reported separately biomarkers that had been compared with PCT and/or CRP.

## Results

**Fig. 1:**
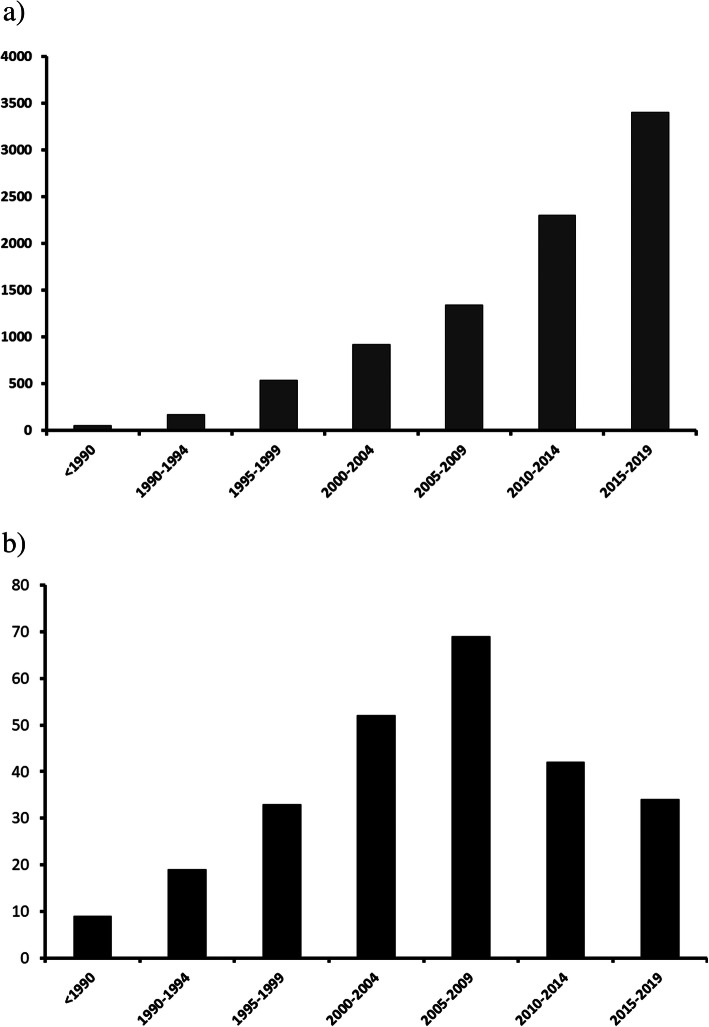
Changes over time in the **a** number of references meeting our search criteria and **b** number of new biomarkers referred to in identified references

A total of 5367 studies met our search criteria for the period 2009 to 2019 compared with the 3370 studies retrieved in our previous study [3]. A total of 80 new biomarkers (54 assessed in clinical studies, 23 in clinical and experimental studies, and 3 in only experimental studies) were added to the list of 178 biomarkers that had previously been identified. Despite a steady increase in the number of published studies related to sepsis biomarkers over time, the number of publications reporting *new* biomarkers has decreased since our prior review (Fig. 1).

The full list of biomarkers with selected references and major findings are shown in Additional file 1, Tables S1–9. Of the 258 biomarkers, 69 (27%) were assessed primarily for their diagnostic value, 100 (39%) for their prognostic value, and 89 (34%) for both diagnostic and prognostic purposes. A validation population was used in just 12 studies. Most of the biomarkers (*n* = 216 [84%]) have been assessed in fewer than five studies, and 81 (31%) have been studied only once. CRP and PCT are the biomarkers that have been studied most frequently, followed by interleukin (IL)-6, presepsin, and CD64 in 31, 25, and 21 studies, respectively.

Apart from CRP and PCT, only 26 biomarkers have been evaluated in studies that enrolled more than 300 patients (Tables 1 and 2). In 15 of these 24 studies (63%), sepsis was defined using either the 1992 ACCP/SCCM [34] or the 2001 International Sepsis Definitions Conference [35] definitions. In one study, the Sepsis-3 definition [7] was used. Other studies used definitions based on clinical signs compatible with sepsis or positive blood cultures. Of the 10 biomarkers evaluated for their diagnostic value in more than 300 patients, 6 (60%) were evaluated using receiver operating characteristic (ROC) curve analysis; the area under the curve (AUC) was > 0.8 for just three of the biomarkers (for inter-alpha inhibitor proteins [11], CD64 [13], and IL-6 [18]). Of the 18 biomarkers evaluated for their prognostic value in more than 300 patients, mortality was the primary study endpoint for 14 (78%); prediction of circulatory failure or organ dysfunction and failure of antibiotic therapy were the primary endpoints in the other studies. ROC curve analysis was used in the analysis of 9 of the 18 biomarkers (50%): the AUC for predicting mortality was > 0.8 only for pro-adrenomedullin, with a high specificity (specificity, 92%; sensitivity, 75%). In two studies, combining a sepsis biomarker with a severity score improved the predictive value (urokinase plasminogen activator receptor [uPAR] + APACHE II AUC, 0.83 [19]; adrenomedullin + Mortality in Emergency Department Sepsis (MEDS) score AUC, 0.81 [27]). All the studies that evaluated more than 300 patients and used ROC analysis had a high risk of bias because a pre-specified abnormal biomarker value was used (Additional file, Table S10).

**Table 1:** Sepsis biomarkers, except for C-reactive protein (CRP) and procalcitonin (PCT), that have been evaluated for their diagnostic value in clinical studies with more than 300 subjects

Biomarker [ref]	No. of patients	Sepsis definition	Study population	Reference group	Sensitivity/specificity (%)	AUC
Interleukin (IL)-27 [10]	702	Positive blood cultures	Pediatric ICU patients with infection	Non-infected critical care patients	84/63	0.75
Inter-alpha inhibitor proteins [11]	573	Positive blood cultures	Neonates with sepsis	Neonates with risk factors for sepsis	89/99	0.9
Group II phospholipase A2 [12]	525	ACCP 1992	ED patients with sepsis	ED patients with suspected infection (with and without SIRS)	NR (logistic regression analysis)	NR
Bactericidal/permeability increasing protein [12]	525	ACCP 1992	ED with sepsis	ED patients with suspected infection (with and without SIRS)	NR (logistic regression analysis)	NR
CD64 [13]	468	International Sepsis Definitions Conference 2001	Non-selected ICU population with sepsis	ICU patients admitted without sepsis	89/87	0.94
Selenoprotein P [14]	378	ACCP 1992	Non-selected population with sepsis or septic shock	Healthy individuals	NR (no test)	NR
Lipopolysaccharide-binding protein [15]	327	ACCP 1992	Surgical patients without sepsis at admission	Surgical patients with SIRS without sepsis	60/62	0.66
Syndecan-1 [16]	512	International Sepsis Definitions Conference 2001	Trauma patients (4 h after admission) without sepsis	Trauma patients without sepsis	NR (logistic regression analysis)	NR
Presepsin [17]	440	Sepsis-3	ICU patients with sepsis	ICU patients without sepsis	89/59	0.76
IL-6 [18]	306	SIRS and organ dysfunction, systolic blood pressure < 90 mmHg, or lactate ≥ 4 mmol/L plus infection	ED patients with suspected sepsis	ED patients with SIRS and organ dysfunction, systolic blood pressure < 90 mmHg, or lactate ≥ 4 mmol/L without infection	NR	0.86

*ED* emergency department, *ICU* intensive care unit, *COPD* chronic obstructive pulmonary disease, *SIRS* systemic inflammatory response syndrome, *NR* not reported, *AUC* area under the receiver operating characteristic curve

**Table 2:** Sepsis biomarkers, except for C-reactive protein (CRP) and procalcitonin (PCT), that have been evaluated for their prognostic value in clinical studies with more than 300 subjects

Biomarker [ref]	No. of patients	Sepsis definition	Study population	Main finding	Sensitivity/specificity (%)	AUC
Urokinase plasminogen activator receptor (uPAR) [19]	1914	International Sepsis Definitions Conference 2001	Critically ill patients and patients hospitalized in internal medicine ward	Levels ≥ 12 ng/mL predicted fatal outcome within 30 days	NR/> 70%	0.708 0.83 (when combined with APACHE II score)
Plasminogen activator inhibitor (PAI) 1 [20]	1790	ACCP 1992	Septic patients with disseminated intravascular coagulation (DIC)	Levels > 90 ng/mL predict fatal outcome within 30 days	NR (Kaplan-Meier survival functions)	NR
Interleukin (IL)-12 [21]	1444	Proven peritonitis or mediastinitis and systemic inflammation signs	Surgical patients	Pre-surgery IL-12-synthesizing capability was low in patients who had fatal sepsis after operation	NR	0.72
Thrombomodulin [22]	1103	ACCP 1992	Critically ill patients with sepsis	Levels > 14 ng/mL can predict circulatory failure or death—gray zone between 7 and 14 ng/mL	NR (logistic regression analysis)	NR
Syndecan-1 [22]	1103	ACCP 1992	Critically ill patients with sepsis	Levels > 240 ng/mL can predict circulatory failure or death—gray zone between 70 and 240 ng/mL	NR (logistic regression analysis)	NR
Fibrinogen [23]	1103	ACCP 1992	Critically ill patients with sepsis	Levels < 200 mg/dL related to increased risk of fatal outcome	NR (logistic regression analysis)	NR
Antithrombin activity [23]	1103	ACCP 1992	Critically ill patients with sepsis	Decrease in activity > 50% related to increased risk of fatal outcome	NR (logistic regression analysis)	NR
Brain natriuretic peptide (BNP) [24]	1000	International Sepsis Definition Conference 2001	ED patients	Levels > 113 pg/mL can predict fatal outcome within 28 days	86/55	0.73
Angiopoietin-2 [25]	931	NR	Critically ill patients with ARDS	Persistently increased levels related to fatal outcome within 90 days	NR (logistic regression analysis)	NR
Prothrombin time (PT) [26]	840	Suspected infection plus ≥ 3 signs of systematic inflammatory response	Critically ill patients with sepsis	Increase in PT time within first 7 days of sepsis was higher in patients who died within 28 days	NR (no test)	NR
Adrenomedullin [27]	837	International Sepsis Definitions Conference 2001	ED patients sepsis	Levels < 34.4 ng/L predicted fatal outcome within 30 days	86/61	0.77 0.81 (when combined with Mortality in Emergency Department Sepsis (MEDS) score)
Pro-adrenomedullin [28]	896	Clinical suspicion of infection	ED patients with sepsis	Levels ≥ 1.6 nmol/L predicted fatal outcome within 28 days	75/92	0.89
Heparin-binding protein [29]	759	Suspected infection and at least one clinical sign of systematic inflammatory response	ED patients with sepsis	Levels > 30 ng/mL predicted any organ dysfunction development within 72 h	78/76 (cross-tabulation analysis)	NR
D-dimer [30]	684	International Sepsis Definitions Conference 2001	Emergency department patients with sepsis	Higher in non-survivors than survivors within 28 days	NR	0.68
Troponin [31]	598	ACCP 1992	Critically ill patients	Levels > 0.06 ng/mL independent prognostic marker for 28-day mortality	NR (logistic regression analysis)	NR
YKL-40 [32]	502	ACCP 1992	Critically ill patients	Levels ≤ 505 ng/mL predicted survival in 90 days	53/76	0.64
CD64 [13]	468	International Sepsis Definition Conference 2001	Critically ill patients	Sustained elevated levels were related to non-appropriate antibiotic therapy	93/48	0.74
Cell-free DNA [33]	481	International Sepsis Definitions Conference 2001	ED patients	Levels > 1.6 μg/mL predicted short-term fatal outcome	70/76	0.77

*ARDS* acute respiratory distress syndrome, *NR* not reported, *IL* interleukin, *SOFA* sequential organ failure assessment, *AUC* area under the receiver operating characteristic curve

Forty biomarkers have been compared with CRP and/or PCT for their diagnostic value (Table 3); 9 were shown to have better diagnostic value and 11 improved the diagnostic value of CRP and/or PCT when used in combination with one of these two biomarkers. In 10 of the 23 studies in which these results were reported (43%), patients with systemic inflammatory response syndrome (SIRS) without infection were selected as the reference group; two studies used patients after major surgery as the reference group. A validation group of healthy volunteers was used in 5 studies (22%).

**Table 3:** Sepsis biomarkers that were compared with procalcitonin (PCT) and/or C-reactive protein (CRP) for sepsis diagnosis

Biomarker	Study group	Reference group	Comment [refs]
**Diagnostic performance similar to or worse than that of PCT and/or CRP**			
Cell-free DNA (cfDNA)	ICU patients with sepsis	ICU patients with SIRS	No better than PCT [36, 37]
Copeptin	ED patients with sepsis	ED patients with SIRS	No better than PCT [38]
ICAM-1	Patients with necrotic pancreatitis	Patients with sterile necrosis	No better than PCT [39]
Lipopolysaccharide-binding protein	ED patients with sepsis	ED patients with infection	No better than PCT [40]
	Non-critically ill patients with sepsis	Non-critically ill patients with infection	No better than PCT [41]
	Children with neutropenia and clinical sepsis and/or bacteremia	Children with febrile neutropenia without infection	No better than PCT [42]
	Patients with proven bacterial lower respiratory infection	Patients with proven viral lower respiratory infection	No better than CRP [43]
	Patients treated in internal medicine ward	Healthy control	No better than PCT [27]
Pancreatic stone protein	ED patients with sepsis	ED patients without infection	No better than PCT [44]
sCD22	Surgical patients with infection after major operation	Surgical patients with SIRS but without infection	Equal value to PCT [45]
Interleukin (IL)-2	ICU patients with sepsis	ICU patients with SIRS without infection	No better than CRP [46]
IL-1β	Neonates with infection and sepsis	Neonates with infection without sepsis	No better than CRP [47]
RANTES	Neonates with infection	Healthy neonates	No better than CRP [48]
Neopterin	ICU patients with sepsis	ICU patients without sepsis	Less accurate than PCT [49, 50]
Macrophage migration inhibitory factor (MIF)	Patients with infection in medical ward or ED	No bacterial infection	No better than PCT [51]
Adrenomedullin	Neutropenic patients with sepsis	Neutropenic patients with fever and clinically documented infection	No better than PCT [52]
Pro-adrenomedullin	Sepsis with organ dysfunction and or shock	Patients admitted to coronary unit without infection	No better than PCT [53]
High-mobility group-box 1 protein (HMGB1)	Infected patients admitted in the ward	Healthy individuals	No better than CRP or PCT [54]
IL-8	Neutropenic children with blood culture positive, and/or fever periods with a documented clinical sepsis and/or local infection	Neutropenic children with fever and no infection	No better than CRP [55]
IL-10	Patients with bacteremia and SIRS,	Patients with SIRS without bacteremia	Comparable with PCT [56]
Endocan	Critically ill patients with sepsis and organ dysfunction	Critically ill patients with infection and SIRS	Comparable with PCT [57]
Pro-atrial natriuretic peptide (ANP)	Burned patients that received antibiotics and had either microbiological confirmation of infection or antibiotics leaded to an improvement in clinical situation	Burned patients without infection	Comparable with PCT [58]
Pentraxin 3	Mechanically ventilated patients with ventilator associated pneumonia	Mechanically ventilated patient > 48 h without VAP	No better than CRP [59]
	Hematological patients with bacteremia and/or septic shock	Hematological patients with fever without infection	No better than CRP [60]
**Better diagnostic value than PCT and/or CRP**			
Thromboelastometry lysis index	Patients with severe sepsis	Patients after operation without sepsis	Better than PCT [61]
Decoy receptor 3	ICU patients with sepsis	ICU patients with SIRS	Positive when PCT was negative [62]
Group II phospholipase A2 (PLA2-II)	ED patients with sepsis and organ dysfunction	ED patients with SIRS without infection	Better than CRP [63]
Hepcidin	Infants with sepsis and or bacteremia	Infants with SIRS and not sepsis	Better than CRP [64]
sCD163	Patients with sepsis admitted to ICU	Patients with SIRS without sepsis	Better than PCT [65]
CD64	ICU patients with sepsis	ICU patients without sepsis	Better than PCT and CRP [66]
	Patients with ventilator associated pneumonia and sepsis	Patients with ventilator associated pneumonia without sepsis	Better than PCT and CRP [67]
Serum amyloid A	Full term infants with sepsis	Full term infants with risk for sepsis but without sepsis	Earlier increase in neonates with early onset sepsis than CRP [68]
Heparin-binding protein	Patients with sepsis for less than 48 h	Patients with infection without sepsis	Better than CRP and PCT [69]
Delta-like canonical Notch ligand 1 (DLL1)	Patients with abdominal infection or surgical site associated infection	Surgical patients, trauma patients without infection, and healthy volunteers	Better than CRP and PCT [70]
**Conflicting findings**			
IL-6	Critically ill patients with sepsis	Patients with SIRS without infection	IL-6 was not found to have lower diagnostic utility compared to PCT (meta-analysis) [71]
	Cirrhotic patients with infection at admission to ICU	Cirrhotic patients without sepsis	IL-6 was found to increase earlier than PCT in cirrhotic patients [72]
sCD25	ED patients with infection	ED patients with suspected infection but finally infection excluded	Equal diagnostic value to PCT for diagnosis of infection in ED [44]
	Patients admitted in ICU with infection and SIRS	Patients with SIRS without sepsis	Better performance than PCT to identify Sepsis I at ICU admission [73]
Calprotectin	ICU patients with infection	ICU patients without sepsis	Better than CRP and PCT [74]
	Patients after major operation who developed sepsis	Patients after major operation who did not develop sepsis	Similar value to PCT [75]
IL-27	Critically ill children with sepsis	Children with SIRS without infection	Better than PCT [76]
	ICU patients with sepsis	ICU patients without sepsis	No better than PCT [77]
sTREM	ICU patients with sepsis	ICU patients with SIRS	Better than PCT [78]
	ICU patients with sepsis	ICU patients with SIRS	No better than PCT and CRP [79]
Presepsin (CD14)	ED patients with sepsis	ED patients with at least two criteria of SIRS without sepsis	Better than PCT in diagnosis of sepsis in ED [80]
	Critically ill patients with sepsis and organ dysfunction	Critically ill patients without infection	No better than PCT regardless of the presence or not of AKI [17]
	Neonates with SIRS and positive blood cultures	Neonates with SIRS with negative blood cultures	Better than PCT [81]
**Better performance when combined with PCT and/or CRP**			
IL-6	Neonates with infection within the first week of life	Neonates with suspicion of infection but finally excluded within the first week of sepsis	Combination with CRP in neonates with suspected sepsis [82]
CD64	Neonates with sepsis	Healthy controls	Combination with PCT and CRP for diagnosis of neonatal sepsis [83]
Leptin	Patients with community acquired pneumonia with sepsis or complicated intraabdominal infection	SIRS without infection, healthy controls	Combination with CRP [84]
Pro-adrenomedullin	Septic patients	Patients with SIRS without sepsis	Combination to PCT [53, 85]
suPAR	Septic patients admitted to ICU	Critically ill patients with SIRS without infection and healthy controls	Combination with PCT for diagnosis of sepsis on day 1 of sepsis [86]
CD11b	Patients with Gram (+) infection	Patients with Gram (−) infection	Combination with CRP for differentiation from Gram (−) infection [87]
Fibrinogen	Neutropenic patients with sepsis	Neutropenic patients with fever without infection	Combination with CRP for diagnosis of sepsis [88]
BNP and antithrombin	Neutropenic patients with fever and bacteremia	Neutropenic patients with fever without infection	Combination with PCT for diagnosis of Gram (−) bacteremia [88]
IL-27	Pediatric patients with sepsis	Pediatric patients with SIRS without infection	Improvement of diagnostic accuracy of PCT for diagnosis of sepsis [77, 89]
α-2 macroglobulin	Surgical patients with sepsis	Surgical patients with SIRS without sepsis	Combination with PCT to exclude sepsis in surgical patients [90]
Decoy receptor 3 and uPAR	Patients with sepsis	Patients with SIRS without infection, healthy volunteers	Combination with PCT for diagnosis of sepsis [91]

*sTREM* soluble triggering receptor expressed on myeloid cells, *RANTES* regulated on activation, normal T-cell expressed, and secreted

Forty-four biomarkers were tested in 55 clinical studies for their use in answering specific, clinically relevant questions rather than simply for diagnosis and/or prognosis of sepsis in general (Table 4): 20 were assessed for use to diagnose infection in specific groups of critically ill patients where diagnosis may be difficult based on clinical evaluation and laboratory values, 8 were assessed for diagnosis of ARDS or associated endothelial damage in patients with sepsis, 6 were tested for their ability to identify specific infections or type of microorganism, 6 were studied for use in the diagnosis of disseminated intravascular coagulation, 4 were assessed for use in deciding which patients with hematological malignancy or neutropenia had a low risk of infection, 3 were assessed for their ability to diagnose infection before any clinical symptoms, 2 were evaluated for use in assessing the risk of delirium or encephalopathy in patients with sepsis, and 1 was assessed to differentiate between sepsis and graft rejection.

**Table 4:** Some examples of biomarkers that have been assessed for use in specific clinical situations

Situation	Biomarker
*To diagnose infection in patients with a particular pathology/condition*	
After cardiac surgery	Endocan [92], CD64 [93], pancreatic stone protein [94]
After major surgery	Peptidoglycan [95], elastase [96], leptin [84], calprotectin [75], a proliferation-inducing ligand [97], α-2 macroglobulin [89], lipopolysaccharide-binding protein [15]
COPD	Pentraxin 3 [98]
Cirrhosis	Interleukin (IL)-6 [72]
Trauma	IL-10 [99], NT-proCNP [100], P-selectin [101]
Catheter-related infections	Citrulline [102]
Infants with necrotic enterocolitis	IP-10 [103]
Neutropenic patients	Lipopolysaccharide-binding protein [104], pro-adrenomedullin [105]
Burns	IL-8 [106], MIF [107]
Autoimmune diseases	CD64 [108]
*To diagnose specific types of infection*	
Gram (−) vs. Gram (+)	Fibrin degradation products [109], lipopolysaccharide-binding protein [104], CD11b [87]
Virus vs. bacterial infection or co-infection	Transforming growth factor (TGF-β) [110], tumor necrosis factor (TNF)-α [111]
VAP	suPAR [112]
*Diagnosis of specific conditions*	
Sepsis vs. graft rejection	Lysozyme [113]
Diagnosis of ARDS	Club cell secretory protein (CC)-16 [114], surfactant protein [114]
Vascular leakage risk in ARDS	von Willebrand factor [115], angiopoietins (1 and 2) [25], IL-8 [116], syndecan-1 [117], HMGB-1 [118]
Recovery from ARDS—endothelial repair	sRAGE [119]
Identification of low risk of infection in hematological/oncological patients	IL-6 [120, 121], IL-8 [120–122], MCP-1 [55], IL-5 [123]
Identification of infection before clinical symptoms	IL-6 [124], IL-ra [125], soluble protein C receptors [126]
Risk of encephalopathy/delirium	VCAM [127], neuron-specific enolase [128]
Disseminated intravascular coagulation	P-selectin [129], protein C [130], microparticles [131], matrix-metalloproteinases [132], thrombin-antithrombin complex [133], a2PI [134]

*COPD* chronic obstructive pulmonary disease, *ARDS* acute respiratory distress syndrome, *TNF* tumor necrosis factor, *VAP* ventilator-associated pneumonia, *NT-ProCNP* N-terminal pro-C-type natriuretic peptide, *MIF* macrophage migration inhibitory factor, *VCAM* vascular cell adhesion molecule, *IP* interferon-gamma-inducible protein, *sUPAR* soluble urokinase plasminogen receptor, *IL-1ra* IL-1 receptor antagonist, *MCP* monocyte chemoattractant protein, *sRAGE* soluble receptor for advanced glycation end products, *HMGB* high-mobility group-box 1 protein

## Discussion

Our literature search illustrates that although new biomarkers have been proposed, little real progress has been made in identifying biomarkers with clinical significance. Using a similar method of searching for sepsis biomarkers to that of our previous study, we noted that the number of publications related to sepsis biomarkers has increased considerably over the years. The proportion of new biomarkers being identified has decreased, but this may reflect publication bias with journals becoming more selective in deciding what merits publication as the volume of these studies increases. Because of the complexity of the sepsis response with multiple mediators, and the improved sensitivity of many tests enabling identification of smaller concentrations of substances than in the past, it is likely that our list of biomarkers will expand further in the future. However, the potential utility of creating an ever-expanding list of potential biomarkers without a more rigorous framework to evaluate them is questionable. An improved methodological approach is needed in order to assess the utility of sepsis biomarkers in daily clinical practice.

Accurate evaluation of the possible clinical utility of a biomarker requires assessment in a large number of patients [5], but we identified only a few biomarkers that have been assessed in studies of more than 300 patients. Moreover, many of the biomarkers have been assessed in only a limited number of clinical studies and one third in just a single study. Patients with sepsis represent a very heterogeneous population, and potential biomarkers need to be assessed in studies with a significant number of patients to ensure random distribution of risk factors that may affect the results of the study (e.g., age, organ dysfunction, type of infection, comorbidities). However, the number of patients enrolled in a study is not the only factor to consider when evaluating the potential role of a sepsis biomarker, and of note, none of the large multicenter studies were able to draw conclusions about the biomarker under study that could change clinical practice.

There was considerable diversity in the methods used to assess sepsis-related biomarkers. Most biomarkers were proposed as being useful for diagnosing sepsis simply because they were increased or decreased to a larger extent in septic than in non-septic patients or healthy individuals. Many studies have assessed the sensitivity and specificity of the biomarker for sepsis diagnosis, but identification of sepsis was often based on the commonly used constellation of non-specific clinical and laboratory findings; in the absence of a “gold standard” diagnostic tool, this method cannot conclusively demonstrate the value of the biomarker with respect to diagnosing sepsis. Other parameters, including positive and negative predictive value or likelihood ratios, can provide greater insight into how well a biomarker performs but these were rarely provided [135]. Similarly, many biomarkers have been used to evaluate sepsis severity using all-cause mortality as the primary endpoint. Importantly, the majority of the studies that evaluated sepsis biomarkers using this method showed only a limited value; it seems highly unlikely that mortality in septic patients is related to only one pathophysiologic process that could be reflected by abnormal levels of a biomarker. Furthermore, the need for another prognostic test can be questioned because clinical data and other laboratory test results, including blood lactate levels, can already reflect severity and the risk of death in septic patients [136]. Prognostic biomarkers may be useful to triage patients in special environments, such as in the emergency room, when the information provided can help clinicians to decide whether hospitalization is necessary and, if so, on the ICU or on the regular floor. However, in a multicenter trial (TRIAGE III) in which emergency room physicians were asked to incorporate the prognostic information portrayed by abnormal uPAR levels into their triage decisions, there was no effect on mortality rates compared to standard practice without uPAR levels [137].

To be of value in clinical practice, a biomarker must be shown to provide an answer to a specific, clinically relevant question, rather than just having diagnostic or prognostic value in general. We identified just 55 studies in which a sepsis biomarker was shown to have a potentially useful role by answering a specific clinical question. For example, biomarkers that could identify specific types of infection may help in guiding a more targeted antibiotic therapy, and a biomarker able to identify septic patients at risk of ARDS may influence fluid management in such patients, reducing risks of fluid overload. Further study needs to better evaluate the potential utility and beneficial effects on outcomes of using biomarkers to answer specific clinical questions.

We attempted to categorize the various biomarkers according to their pathophysiological role, although for many it was not possible to identify a clear role, and some have multiple roles. Only a few biomarkers were found to have a role specifically related to sepsis pathophysiology rather than to a more general inflammatory reaction, including presepsin (the N-terminal fragment of the macrophage lipopolysaccharide [LPS] receptor), LPS-binding protein (LBP), bactericidal/permeability increasing protein, peptidoglycan, thrombomodulin, and anti-endotoxin core antibodies. Such biomarkers may help transform our understanding of sepsis from a “physiological syndrome to a group of distinct biochemical disorders” [138] and advance our search for adjunctive sepsis therapies.

CRP and PCT are by far the most widely used and studied biomarkers. Both increase transiently during sepsis, but long enough to allow for their detection, reflecting a real-time response. Although PCT is considered superior to CRP in many studies [139, 140], it is not a definitive test for diagnosing sepsis because PCT levels can also be increased in other conditions [141]. PCT, similar to CRP, may be more useful to rule out sepsis than to diagnose it [142–144], and the combination of these two biomarkers may improve their ability to exclude sepsis [145]. Studying the time course of these biomarkers may also be helpful to evaluate an individual patient’s response to therapy. Changes in serum CRP levels during the first 48 h after antibiotic initiation can help evaluate the response to initial antimicrobial therapy [146]. Likewise, a PCT-based algorithm may help reduce antibiotic exposure in septic patients without compromising clinical outcomes [147, 148]. However, not all studies have shown the same positive effect [149], suggesting that the effectiveness of PCT-based algorithms may depend on the physician’s experience and the clinical setting. Some biomarkers have been compared to PCT and CRP, most for their diagnostic value. A few were shown to be superior to PCT and/or CRP for this purpose, for example, presepsin and CD64 [66, 67, 150].

Measuring several biomarkers concurrently may be useful to overcome the limitations of any single biomarker. Combining biomarkers that are involved in different sepsis-related pathways may be particularly attractive. A seven-biomarker panel including cellular markers and interleukins correctly identified 89% of patients with ventilator-associated pneumonia (VAP) and 100% of patients without VAP [151]. Similarly, a combination of several sepsis-related biomarkers (PCT, presepsin, galectin-3, and soluble suppression of tumorigenicity 2) was found to have better prognostic value than PCT alone [152]. However, it is not clear from the existing literature whether the biomarkers included in such panels should be selected based on pathophysiological or other criteria. The combination of a biomarker panel with clinical information may be particularly useful in the diagnosis of sepsis or in the risk stratification of patients with sepsis [153].

The study has some limitations that should be acknowledged. First, although we performed an extensive search, we cannot be sure that some studies were not missed. Nevertheless, the large number of sepsis biomarkers that we retrieved suggests that we managed to identify the majority of the biomarkers that have been studied. Second, we included studies over a long period of time, during which the definition of sepsis has changed so that it is difficult to make comparisons. Third, it is difficult to compare different biomarkers because the methods used to evaluate the biomarkers and to define sepsis and the populations studied varied across the studies.

## Conclusions

Since our original search, many additional sepsis-related biomarkers have been identified. However, the precise roles of most biomarkers in the management of septic patients have not been well defined, and of the many biomarkers that have been studied, only a few have been evaluated in large or repeated studies. As such, it is not possible to draw any reliable conclusions about which compounds could be considered as the most “promising” candidates. Even the biomarkers that had an AUC > 0.8 for diagnosis or prognosis, making them potentially more interesting for further study, were evaluated in studies with a high risk of bias. Moreover, while there are multiple putative biomarkers, rarely have they been compared against each other to determine how they differ in what they are measuring, and which does it better. Almost all studies report a single marker in isolation, but given the complexity of sepsis, surely these markers are not biologically independent, so how can we know which is best to use?

It is therefore important to develop a more rigorous, standardized methodology to assess sepsis biomarkers and identify those that can provide valuable, clinically relevant information. Such an approach could include the following factors:

1. What is the question being asked?
  1. Greater likelihood of infection leading to administration of empiric antibiotics or performance of a diagnostic test (e.g., carcinoembryonic antigen [CEA] levels are used to detect early recurrence in patients with colon cancer, and so guide further investigations)
  2. Resolution of infection and therefore safety in stopping antibiotics
  3. Increased likelihood of benefiting from specific interventions, such as steroids or a biologic agent
  4. Increased risk of adverse outcome not apparent by other evidence
  5. Ensuring random distribution of risk factors in a randomized controlled trial
2. How is the study designed?
  1. What is the control group
  2. Which patients and how many are being studied
  3. How are outcomes adjudicated
  4. Is there a validation cohort
  5. Uniform techniques to evaluate results—sensitivity, specificity, positive and negative predictive values, likelihood ratios, and ROC analysis
3. Is the marker biologically plausible, and what do alterations tell us about the pathobiology of disease in this patient?

Consideration of these factors and their application to sepsis biomarker research may help identify new biomarkers with real clinical utility. Continuing to produce reports of novel biomarkers without developing a more rigorous framework to evaluate them and establishing a recognized purpose is futile: it is time for a reappraisal of the possible roles of biomarkers in sepsis.

## Supplementary information

**Additional file 1: Figure S1.** Biomarkers of sepsis: Time for a reappraisal Pierrakos et al. **Table S1.** Cytokine/chemokine biomarkers identified in the literature search. **Table S2.** Receptor biomarkers identified in the literature search. **Table S3.** Cell marker biomarkers identified in the literature search. **Table S4.** Coagulation-related biomarkers identified in the literature search. **Table S5.** Microcirculation related biomarkers identified in the literature search. **Table S6.** Vasodilation-related biomarkers identified in the literature search. **Table S7.** Biomarkers of organ dysfunction in sepsis identified in the literature search. **Table S8.** Acute phase proteins used as biomarkers in sepsis identified in the literature search. **Table S9.** Diverse sepsis biomarkers identified in the literature search. **Table S10**. QUADAS-2 score [1145] for quality assessment for the studies that included >300 patients where ROC curve analysis was used.

## Data Availability

Not applicable.

## Abbreviations

AUC: Area under the curve
CRP: C-reactive protein
ICU: Intensive care unit
IL: Interleukin
LBP: Lipopolysaccharide-binding protein
LPS: Lipopolysaccharide
MEDS: Mortality in Emergency Department Sepsis score
PCT: Procalcitonin
ROC: Receiver operating characteristic
uPAR: Urokinase plasminogen activator receptor
