# Supplementary material for: Biomarkers of sepsis: time for a reappraisal
Source: Crit Care. 2020 Jun 5;24:287. doi: 10.1186/s13054-020-02993-5 (PMC7273821; doi:10.1186/s13054-020-02993-5)
Supplement: Supplementary file 1 — Additional file 1: Figure S1. Biomarkers of sepsis: Time for a reappraisal Pierrakos et al. Table S1. Cytokine/chemokine biomarkers identified in the literature search. Table S2. Receptor biomarkers identified in the literature search. Table S3. Cell marker biomarkers identified in the literature search. Table S4. Coagulation-related biomarkers identified in the literature search. Table S5. Microcirculation related biomarkers identified in the literature search. Table S6. Vasodilation-related biomarkers identified in the literature search. Table S7. Biomarkers of organ dysfunction in sepsis identified in the literature search. Table S8. Acute phase proteins used as biomarkers in sepsis identified in the literature search. Table S9. Diverse sepsis biomarkers identified in the literature search. Table S10. QUADAS-2 score [1145] for quality assessment for the studies that included >300 patients where ROC curve analysis was used. [file 13054_2020_2993_MOESM1_ESM.pdf]

Biomarkers of sepsis: Time for a reappraisal

Pierrakos et al.

Additional file 1

Figure S1. Simple schematic of the main pathophysiological pathways influenced by the sepsis biomarkers that have been proposed

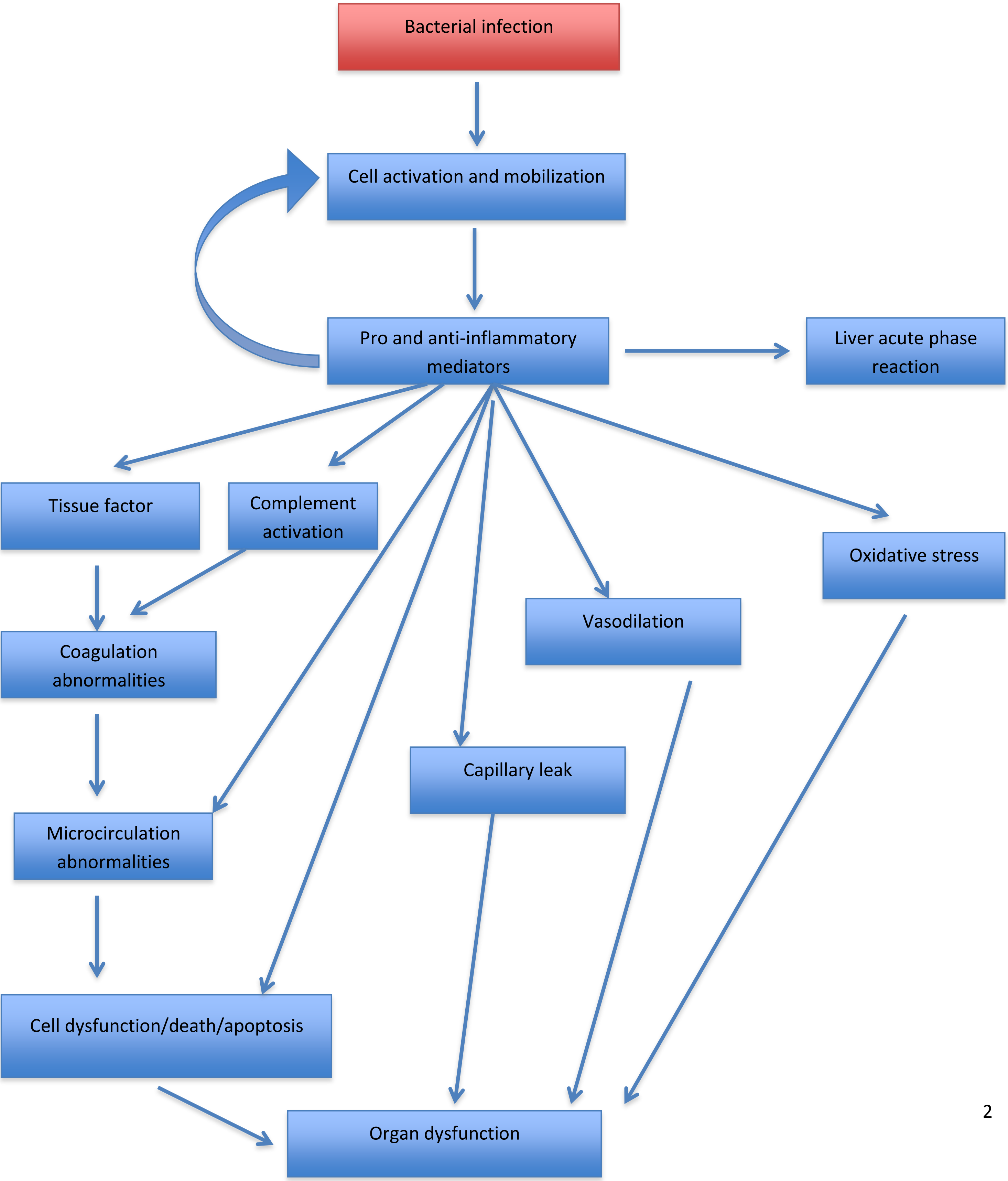

Table S1 Cytokine/chemokine biomarkers identified in the literature search (with some selected references)

**Name [refs]**	**Main pathophysiologic role**	**Main findings**
IL-1 receptor antagonists [1-7]	Bind to IL-1 receptors. Anti-inflammatory action [6]	Earlier diagnosis of sepsis in critically ill patients compared to conventional criteria. Predictor of poor outcome. Levels decreased with effective resuscitation
High mobility group box 1 (HMGB1) [8-21]	Pro-inflammatory cytokine secreted by macrophages several hours after their stimulation [20]	Increased 24 h after the initiation of treatment compared to baseline values in non-survivors. Peak observed 48 h after diagnosis
Tumor necrosis factor alpha (TNF-α) [22-27]	Pro-inflammatory cytokine- increases in endothelial permeability [28]	Correlation with APACHE II score
TNF-related weak inducer of apoptosis [29]*	Inflammation amplification, cell death inducer [30]	Higher in non-survivors. Lower levels in critically ill patients with sepsis compared to non-sepsis
A proliferation-inducing ligand [31, 32]*	B-cell activation [33]	Distinguished between survivors and non-survivors; lower in critically ill patients with sepsis compared to non-septic on admission.
Interleukin (IL)-1 beta [24, 34-41]	Pro-inflammatory cytokine released early in sepsis [42]	Conflicting results for identification of bacteremia in infants
IL-2 [43]	Promoting T-cell growth factor [44]	Lower in septic than non-septic patients
IL-4 [45-47]	Proliferation of Th2 cell production. IL-4 might also act as a barrier- sustaining agent to decrease the migration of bacteria	Low values on admission day associated with greater incidence of nosocomial pneumonia

	across the intestinal epithelium [48]	
IL-5 [49, 50]*	Cytokine for eosinophil growth and maturation and neutrophilic recruitment [51]	Increased in neutropenic children with septicemia
IL-6 [5, 11, 52-82]	Initiation of acute phase response [83]	Higher in patients with SIRS and infection compared to other critically ill patients. Persistent increase related to failure of sepsis treatment
IL-8 [78, 79, 84-88]	Regulator of neutrophil migration [89]	High serum levels predicted septic complication in pediatric patients
IL-10 [86, 90-97]	Repress expression of pro-inflammatory cytokines, mainly interferon -γ (IFN-γ) [98]	Diagnosis of septicemia - increased in non-survivors
IL-12 [99-103]	Early inflammatory action-production of IFN-γ [104]	Low IL-12 production after stimulation of whole blood with LPS predicted death from postoperative sepsis
IL-13 [105, 106]	Inhibits IL-6 secretion and TF expression [21, 107]	Higher in non-surviving septic patients
IL-15 [108]*	Blockage of TNFR-1 mediated apoptosis [109]	Persistently elevated levels in non-survivors
IL-17 [110]*	Pro-inflammatory cytokines that activate tissue-infiltrating neutrophils [111]	Higher admission values in trauma patients that later developed sepsis.
IL-18 [112-115]	Induction of IF-1 production by Th1 cells [116]	Distinguished between survivors and non-survivors. Higher in septic surgical patients compared to non-septic

IL-27 [117-120]*	Production from APC for early stimulation Th1 cells [121]	Helpful in the diagnosis of septicemia in combination with PCT in pediatric patients
IL-37 [122]*	Suppresses innate inflammatory reaction by binding to IL-18 receptor [123]	Increased in patients with sepsis compared to healthy controls
Pre B cell-colony enhancing factor (PBEF) [124-128]	Pro-inflammatory, shown to increase inflammatory cytokines, such as TNF-α, IL-1β, IL-16, and TGF-β1, and the chemokine receptor CCR3 [129]	Elevated levels in non-survivors
Monocyte chemotactic protein 1 [50, 87, 130-136]	Monocyte-macrophage recruitment and activation [137]	Increased in parallel with disease severity, prediction of 6-month mortality
Osteopontin [138-141]	Monocyte-macrophage recruitment and stimulation [142]	Persistent elevation in non-surviving septic patients. Diagnosis of sepsis in the emergency department
Leukocyte cell-derived chemotaxin 2 (LECT2) [143]*	Inhibits production of IL-1β, TNF-α, IL-6 [144]	Increased progressively in survivors. Lower in septic patients compared to healthy volunteers
GRO-alpha [145, 146]	Neutrophil recruitment [147]	Distinguished between survivors and non-survivors
Macrophage migration inhibitory factor (MIF) [134, 148-153]	Inhibition of macrophage migration and stimulation of secretion pro-inflammatory cytokines. Activation T-cells [154]	Prediction of death within 48 h. Limited diagnostic value
Regulated upon Activation Normal T cell Expressed and Secreted (RANTES) [155-158]	Chemotactic cytokine-angiogenetic factor [159]	Early diagnosis of sepsis in neonates: contradictory results
IP-10 [160, 161]	Chemokine secreted from cells stimulated with IFN-γ	Higher in neonates with necrotizing enterocolitis and sepsis compared

	and LPS. Plays an important role in recruiting activated T-cells [162]	to those with necrotizing enterocolitis without sepsis. Correlation with severity of acute respiratory infection.
Transforming growth factor (TGF-β) [163-165]	Suppresses the proliferation and differentiation of T- and B-lymphocytes and antigen presenting cells, and promotes fibrosis [166]	Distinguished between survivors and non-survivors with ARDS. No prognostic value in patients with Gram + infection. Differentiated influenza A (H1N1) infection from other causes of community-acquired infection
CXCL12 [167-169]*	Neutrophilic chemoattractive chemokine [170]	Higher in septic neonates and adults compared to non-septic. Predictive of death.
Macrophage inflammatory protein (MIP)-1 and -2 [88, 164, 171, 172]	Chemokine secreted from hematopoietic cells and fibroblasts. Secretion induced by endotoxin, interleukin (IL)-1, and tumor necrosis factor (TNF)-α. Plays a role in the regulation of leukocyte activation [171]	Distinguished between survivors and non-survivors at 28 days.

*new biomarker since earlier review

Table S2 Receptor biomarkers identified in the literature search (with some selected references)

**Name**	**Main pathophysiologic role**	**Main findings**
CRTh2 [173]	PGD_2 receptor on Th2 cell receptor-role in Th2 chemotaxis [174]	Lower in septic patients compared to healthy volunteers. Persistent low expression in non-survivors
Serum CXCR-4 [167, 169, 175]*	Neutrophil receptor of CXCL-12. Important for neutrophil mobilization [170]	Higher in septic neonates and adults compared to non-septic. Decreased 48h-72h after treatment initiation
CX3CR1 [176, 177]	Fractalkine receptor on monocytes. Important for monocyte recruitment to epithelial wall [178]	Persistently lower expression in non-survivors
Decoy receptor 3 [179-182]*	Soluble receptor of FasL secreted by APC after TLR-4 activation. Protection of lymphocytes from apoptosis [183]	Higher compared to patients with systemic inflammation without sepsis. Improved diagnostic value when combined with PCT and suPAR. Correlation with APACHE II score. Persistent increase in non-survivors
Soluble Fas [184-189]	Prevention of lymphocyte apoptosis [190]	Correlation to SOFA score
Soluble FLT-1 (VEGFR1) [191-194]	VEGF neutralization [195]	Correlation to SOFA score
Soluble and bound CD163 [54, 196-202]	Macrophage activation marker-T-lymphocyte proliferation inhibition [202]	Higher values in septic patients compared to non-septic critically ill patients. However, no diagnostic value in trauma patients. Distinguished between survivors and non-survivors
Soluble mannose receptor [198, 203, 204]*	Macrophage activation marker [205]	Distinguished between survivors and non-survivors. Higher in septic patients compared to non-septic critically ill patients.

Soluble protein C receptors [206-209]*	Inhibition of activated protein C anticoagulant activity [210]	Elevated levels on admission to ICU predicted sepsis development. Distinguished between survivors and non-survivors
Soluble Receptor for Advanced Glycation Endproducts (RAGE), (sRAGE) [211-217]	Competition with cellular RAGE neutralizing AGE actions [218]	Correlation with SOFA score
Soluble TNF receptors (1&2) [219-221]	Neutralization of TNF alpha- can be cleaved from the cell surface by the matrix metalloproteinase family in response to inflammatory signal [222]	Correlation with SOFA score. Higher in septic patients compared to non-septic healthy or critically ill patients. Validity has been assessed in neonates too.
Soluble triggering receptor expressed on myeloid cells (TREM)-1 [54, 201, 223-233]	Soluble form of TREM-1 secreted by phagocytes [234]	Diagnosis of septicemia- Distinguish between survivors and non-survivors
Soluble urokinase plasminogen activator receptor (suPAR) [235-253]	Facilitate recruitment of neutrophils and monocytes [254]	Distinguished between survivors and non-survivors. Improvement of APACHE II score predictive value. Early identification of bacteremia and VAP. Improve diagnostic value of PCT. Limited diagnostic value as sole biomarker in non-selected critically ill population.
CCR3 [173, 255]	Eotaxin receptor on Th2 cell - role in Th2 chemotaxis [173]	Lower serum levels in septic patients compared to healthy volunteers-Persistent low expression in non-survivors
Soluble IL-2 receptor [54, 256]	Indicative of T-cell activation [257]	Prediction of multiple organ failure development in patients with septic shock
Soluble tumorigenicity 2 (ST2) [258-262]	Attenuate Th2 response by binding to IL-33 [263]	Higher when compared to non-septic trauma or surgical patients. May improve prognostic value of

		PCT
Neutrophilic Toll-like receptor (TLR) 2 [264-266]	Recognition of bacterial peptidoglycan [267]	Higher in patients with sepsis compared to healthy subjects
Complement 5a receptors (C5aR) (cellular and soluble) [268-271]	Receptor of C5a-Complex C5a-C5aR significant for neutrophil migration, apoptosis diminution, coagulopathy reversion [272]	Cellular form: Negative correlation to sepsis severity<br>Soluble form: Increased levels in non-survivors
NKG2D [273]*	Receptor on NK cells-pathogen recognition [274]	Higher than in non-septic critically ill patients
CD28, cellular and soluble [275]	Costimulatory receptor of T-cells [276]	Distinguished between survivors and non-survivors
CD64 [277-299]	High affinity receptor for the Fcγ part of the IgG heavy chain; process related to bacterial phagocytosis; expressed on activated neutrophils [300]	Higher in septic patients compared to non-septic critically ill patients. Sustained elevated levels were related to inappropriate antibiotic therapy.
sCD127 [301]*	Diminish excessive IL-7 signaling [302]	Distinguished between survivors and non-survivors
Presepsin [261, 303-326]*	N-terminal fragment of soluble CD14 (a LPS receptor on macrophage) [327]	Higher in patients with bacterial infection compared to those without bacterial infection. Earlier increase than PCT and CRP in neonates. Comparable diagnostic value with PCT in adults without acute kidney injury. Day-1 values distinguished survivors from non-survivors. Improved prognostic value of PCT. Changes in the levels used to monitor effectiveness of treatment and decision making to continue antibiotic treatment.
CD35 [328-330]	Complement (C4) receptors on dendritic cells. Relation to B-	Distinguished between febrile

	cell activation [331]	bacterial and viral infections
IL-1 receptor 2 [258-260, 332]*	Decoy receptor of IL-1 that inactivates IL-1 [333]	Differentiated critically ill patients with sepsis from patients with SIRS; more efficient for Gram (-) infections
Programmed death-1 (PD-1) receptor [334]*	Cell surface receptor. Negative regulator of immune response [335]	Higher in patients with sepsis compared to healthy controls. Higher in non-survivors septic patients.
CD40, cellular and soluble [275, 336]	Member of tumor necrosis factor (TNF) receptor superfamily. Found on activated macrophages and platelets [337, 338]	Increased in parallel with disease severity.
GP130 [339, 340]	A key receptor regulating activation of the acute-phase response through interleukin (IL)-6 [339]	Increased in sepsis compared with healthy controls
TRPV1 receptor [341]	Receptor situated on leukocytes, macrophages, and endothelial cells. It is related to bacterial phagocytosis and vessel vasodilation [342]	Increased in sepsis compared with healthy controls

*new biomarker since earlier review

Table S3 Cell marker biomarkers identified in the literature search (with some selected references)

Name	Main pathophysiologic role	Main findings
CD10 [343-345]	Lowers expression on immature neutrophil surface [343]	Prediction of patient degradation within 48h after sepsis onset
CD11b [284, 294, 346-351]	Regulation of neutrophil adhesion and migration [352]	Higher in neonates and adults with sepsis compared to healthy volunteers-High negative predictive value in combination with CRP
CD11c [353, 354]	Regulation of neutrophil adhesion and migration [355]	Higher in patients with sepsis compared to patients with non-infectious systematic inflammatory syndrome or healthy controls
CD16 [343, 356]	Negative effect on human monocyte capacity to phagocyte Gram-bacteria [357]	Prediction of patient degradation within 48h after sepsis onset
CD18 [358, 359]	Leukocyte, surface bound antigen. Soluble form is result of seeding from surface antigen induced by TNF-α [359]	Distinguish surviving and non-surviving septic patients
Soluble CD22 [360]*	Fragment of CD22 that is B-cell membrane protein. CD22 acts as an inhibitory coreceptor for B cells. [360]	Higher in patients with intraabdominal infection compared to controls. Correlated with APACHE II score
Soluble CD25 [91, 361-365]	Marker of T regulatory cells; indicative of their expansion [365]	Higher in septic patients compared to non-septic critically ill patients. Prediction of AKI development
CD31 [366]*	Marker of T-cells with angiogenic capacity	Inversely related to SOFA score-Higher in septic patients compared

	related to endothelial repair [367]	to healthy volunteers
CD39 [368]*	Catalyzes the phosphohydrolysis of pro-inflammatory extracellular ATP [369]	Distinguished between survivors and non-survivors
CD41 [370]*	Platelet specific antigen [371]	Distinguished between patients with sepsis secondary to fungal infection and of other etiology
CD48 [372]	Upregulated in activated immune cells. Participates in cell adhesion [373]	Higher expression on monocytes and neutrophils in septic patients compared to those of healthy volunteers. Lower expression on lymphocytes of septic patients compared to those with viral infection
CD80 [275]	Expressed on APC- Co-stimulator factor of T-cells [374]	Higher compared to healthy volunteers. Higher in patients with septic shock compared to septic patients without septic shock
Human leukocyte antigen-G5 (HLA-G5) [375, 376]	Antigen with anti-inflammatory activities (inhibits cytolytic functions of NK and T-cells) [377]	Distinguished between survivors and non-survivors
Human leukocyte antigen-DR (mHLA-DR) [292, 378-381]	Expressed on APC. Related to activation of T-cells [382]	Persistently low levels related to fatal outcome. Limited diagnostic value. Diagnosis of sepsis in combination with PCT in surgical patients
CD69 [383]	Expressed on T-cells early after their activation. Co-stimulatory factor for T-cell activation and proliferation [384]	Higher compared to healthy volunteers

L-selectin [385-388]	Found on neutrophil membrane and contributes to their adhesion to endothelial cells and transmigration across them [389]	Distinguished between survivors and non-survivors. Lower than in healthy volunteers
G protein-coupled receptor (GCPR) [390]*	Chemokine receptor expressed on neutrophils, related to neutrophil migration capacity [391]	Distinguished between survivors and non-survivors

*new biomarker since earlier review

Table S4 Coagulation-related biomarkers identified in the literature search (with some selected references)

**Name**	**Main pathophysiologic role**	**Main findings**
Antithrombin III, and antithrombin activity [392-395]	Coagulation inhibition and anti-inflammatory activity [396]	Distinguished between survivors and non-survivors
Activated partial thromboplastin time (aPTT) (transmittance waveform) [397, 398]	Value depending on CRP-VLDL complex formation. Indicative of CRP activity [399]	Distinguished between septic patients and non-septic critically ill patients
D-dimer [400-402]	Breakdown of fibrinogen and fibrin. Indicative of excessive coagulation activation [400]	Distinguished between survivors and non-survivors
Thrombin-antithrombin complex [403, 404]	Marker of thrombin generation [405]	Distinguished between survivors and non-survivors. Diagnosis of DIC in patients with sepsis
Prothrombin time (PT) [400]	Indicative of consumption and depletion of endogenous hemostasis factors [394]	Distinguished between survivors and non-survivors
Fibrin degradation products [406, 407]	Indicative of excessive coagulation activation [408]	Distinguished between survivors and non-survivors. Higher in septic patients compared to other critically ill patients
Plasminogen activator inhibitor (PAI) 1 [409-412]	Fibrinolysis inhibition [412]	Distinguished between survivors and non-survivors in sepsis-induced DIC
Protein C (PC) [403, 413-420]	Antithrombotic action via inactivation factors V and VIIIa [421]	Distinguished between patients with sepsis and patients with pneumonia without sepsis. Predictive of complications
Alpha-2 plasmin inhibitor (a2PI) [422]	Fibrinolysis inhibitor [423]	Early diagnosis of DIC in septic patients

Fibrinogen [395, 424]	Low activation of secondary fibrinolysis [425]	In combination with CRP, useful in diagnosis of severe sepsis in neutropenic patients
Thromboelastometry lysis index [426]	Indicative of hypercoagulable state [427]	Higher in septic surgical patients compared to non-septic surgical patients; better diagnostic capacity than PCP and CRP
Fibronectin [428-431]*	Clot stabilization, platelet activation and cell adhesion and invasion [432]	Lower in septic patients compared to non-septic patients
ADAMTS-13 [433-440]	von Willebrand factor cleaving protease [441]	Predicted organ dysfunction and mortality in septic patients
von Willebrand factor [440, 442-444]	Platelet adhesion and accumulation promotion [445]	Distinguished between survivors and non-survivors
Microparticles [446-448]	Fragments originating from plasma membrane, secreted by activated cells with procoagulation activity [449]	Development of DIC in patients with septic shock
Beta-thromboglobulin [450]	Platelet specific protein secreted by activated platelets. Proteolytic product of platelet factor 4 [451]	Higher in septic patients compared to healthy controls
Plasmin alpha2-antiplasmin complex [452]	A stable inactive complex; marker of plasmin production and fibrinolytic activation [453]	Prediction of MOF development
TIMP-1 [454-457]	Natural inhibitor of matrix metalloproteinases with anti-fibrinolytic function [458]	Distinguished between survivors and non-survivors

Tissue factor [459]	Transmembrane glycoprotein, is expressed by various cell types that activates coagulation cascade by binding to the factor VIIa [460]	Higher in patients with sepsis-induced ARDS compared to other patients with ARDS. Distinguished between survivors and non-survivors
Platelet factor 4 [461]	Platelet secreted protein, indicative of platelet activation [451]	Higher compared to healthy volunteers
Reduced coagulation factor XI (rFXI) [462]	Coagulation factor related to intrinsic coagulation system [463]	Increased in septic patients compared to healthy controls. Correlation with disease severity

*new biomarker since earlier review

Table S5. Microcirculation related biomarkers identified in the literature search (with some selected references)

**Name**	**Main pathophysiologic role**	**Main findings**
Angiopoietin-1, 2 [136, 464-472]	Secreted by peri-endothelial cells. Activation of Tie2 related to vascular integrity [473]. Angiopoietin-2 is an Angiopoietin 1 antagonist; binds to Tie-2 without inducing signal transduction [474]	Distinguished between survivors and non-survivors
Endothelial leukocyte adhesion molecule (ELAM)-1 [475, 476]	Endothelial specific molecule which mediates leukocyte adhesion to endothelium [476]	Higher in septic patients compared to non-septic trauma patients. Distinguished between survivors and non-survivors
Circulating endothelium progenitor cells, (cEPC) [477, 478]	Related to endothelium repair [479]	Higher in septic patients compared to healthy volunteers
Intercellular adhesion molecule (ICAM) [385, 480-486]	Contribute to neutrophil adhesion and transendothelial migration [487]	Diagnosis of sepsis in neonates. Correlation with the severity of sepsis.
Vascular cell adhesion molecule (V-CAM) 1 [385, 488-490]	Adhesion protein expressed by endothelial cells which binds to lymphocytes [491]	Prediction of sepsis encephalopthy development
Vascular adhesion protein (VAP)-1 [492]	Transmembrane glycoprotein in the vascular wall with two functions: enzymatic activity as monoamine oxidase and an adhesion molecule for lymphocytes [493]	Higher in septic patients compared to healthy controls
Serum laminin [494]	Component of extracellular matrix lamin derived by its	Distinguished between fungal and bacterial infection

	hydrolization [494]	
Syndecan-1 [492, 495-498]*	Glycocalyx component-Indicative of glycocalyx damage [499]	Distinguished between survivors and non-survivors. Early identification of sepsis complications in trauma patients. Prediction of respiratory failure in patients with sepsis due to pneumonia
Hepatocyte growth factor (HGF), cellular and soluble [500-502]	Pleiotropic factor synthetized by vascular cells and stimulates endothelial cells growth [503]	Response to antibiotic treatment
Neopterin [199, 504, 505]	Pyrazino-pyrimidine compound derived from GTP secreted by macrophage and endothelial cells after stimulation by TNF-α and INF-γ [506]	Higher in septic patients compared to non-septic critically ill patients
Platelet-derived growth factor, PDGF–BB [507]	Stimulates the proliferation of endothelial cells [508]	Distinguished between survivors and non-survivors
Vascular endothelial growth factor, VEGF, VEGF-A [191, 509-513]	Stimulates the proliferation of endothelial cells and inhibits endothelial apoptosis [514]	Distinguished between survivors and non-survivors; higher levels compared to healthy volunteers
E-selectin [515-518]	Arrest and facilitation of the migration of activated neutrophils at sites of vascular inflammation [519]	Prediction of acute kidney injury in critically ill patients with severe sepsis
Cysteinyl-leukotriene [520]	Eicosanoid derived from arachidonic acid via the 5-lipoxygenase pathway causing vascular leakage and neutrophil extravasation [521]	Distinguished between survivors and non-survivors
Heparin binding protein [522-	Protein founded in azurophilic granulae of	Prediction of MOF development. Monitoring response to treatment

530]*	neutrophils and secreted in the presence of bacteria; induces vascular leakage [531]	Higher in patients with sepsis compared to patients with infection or healthy controls
Raftlin [532]*	Major protein in lipid rafts produced by the endothelial protein C receptor-dependent cleavage of protease activated receptor 1 [533]	Higher compared to healthy controls; correlation with sepsis severity
Xanthine oxidase [534]	Oxygen dependent enzyme; adherence of activated polymorphonuclear neutrophils with endothelial cells lead to conversion of xanthine dehydrogenase to xanthine oxidase within the endothelium leading to the killing of endothelial cells [535]	Distinguished between survivors and non-survivors
Thrombomodulin [495, 517, 536, 537]	Membrane glycoprotein expressed on endothelial cells, which plays a major role in the protein C anticoagulation pathway [538]	Early prediction of MOF. Distinguished between survivors and non-survivors
Endocan [440, 539, 540, 540-544]	Soluble peptidoglycan that is expressed in human endothelial cells in response to pro-inflammatory cytokines. Enhances production of pro-inflammatory cytokines, increases microvascular permeability [545]	Persistent increased levels predicted death in patients admitted to ICU with sepsis. Diagnosis of pulmonary infection after cardiac surgery 48h before conventional diagnosis in patients with chronic kidney disease
14 kDA endocan fragments	Indicative of endocan	Higher in septic patients compared

[546, 547]	proteolysis [546]	to healthy volunteers

*new biomarker since earlier review

Table S6. Vasodilation-related biomarkers identified in the literature search (with some selected references)

**Name**	**Main pathophysiologic role**	**Main findings**
Adrenomedullin [547-555]	Peptide secreted by vascular smooth muscle cells and vascular endothelial cells that induces vasodilation mainly via generation 3,5cAMP [556]	Distinguished between survivors and non-survivors. Increased vasopressor needs
Pro-adrenomedullin [308, 551, 557-567]	Fragment of precursor of Adrenomedullin [558]	Distinguished between survivors and non-survivors. Prediction of organ failure. Improved the diagnostic value of PCT
Pro-vasopressin [558]*	Fragment of precursor of vasopressin [558]	Distinguished between survivors and non-survivors, in patients with septic shock
C-type natriuretic peptide [565, 568-572]	Secreted by endothelium and hyperpolarizes the underlying smooth muscle causing vasodilation [573]	Early diagnosis of sepsis in trauma patients. Treatment response monitoring. Higher in septic patients with brain lesions on MRI compared to septic patients without brain lesions
Substance P [574-576]	Secreted by macrophage lymphocytes and eosinophils; Acts via NK-1R receptor and has pro-inflammatory and vasodilatory activities; Increases also the vessels permeability [577]	Distinguished between survivors and non-survivors
Tetrahydrobiopterin [578, 579]	Endothelial nitric oxide synthase co-factor [580]	Higher in septic patients compared to non-septic critically ill patients

Vasoactive intestinal peptide (VIP) [581-583]	Neuropeptide that has vasodilatory activity by increasing adenylyl cyclase activity and cAMP concentration [584]	Higher in patients with septic shock compared to septic patients without shock
Elastin peptides [585]	Prostanoid synthesis and increased NO [586]	Lower in septic patients compared to healthy volunteers
Nitrite/nitrate [587-589]	NO bioreactor products [590]	Higher in patients with septic shock than patients with cardiogenic shock
CGRP [574, 591, 592]	Vasodilatory activity by increasing adenylyl cyclase and NO [593]	Correlation to APACHE II score
47kD HK [594]	Cleaved product of high molecular weight kininogen (bradykinin precursor molecular); indicator of bradykinin release [594]	Distinguished between survivors and non-survivors
Asymmetric dimethylarginine (ADMA) [595-597]*	Nonspecific NO synthase inhibitor [598]	Distinguished between survivors and non-survivors
FAAH mRNA [599]*	Enzyme which converts anadamide (a potential vasodilator) to arachidonic acid [600]	Lower in septic patients compared to healthy controls. Prediction of septic shock development
Hemoglobin subunit beta (Hbβ) [601]*	Component of Hemoglobin related to NO transfer and release [602]	Higher in septic shock patients compared to healthy controls
Cyclic nucleotides [603]	Single phosphate nucleotide. Producing vascular smooth muscle relaxation and vasodilatation [604]	Distinguished between survivors and non-survivors at 28 days
Neuropeptide Y [591, 605]	Vasoconstrictor peptide secreted by perivascular	Higher in patients with sepsis compared to healthy volunteers

	nerves [606]	

*new biomarker since earlier review

Table S7. Biomarkers of organ dysfunction in sepsis identified in the literature search (with some selected references)

Name	Main pathophysiologic role	Main findings
Pro-atrial natriuretic peptide (ANP) [607-612]	Secreted by atrial distention [613]	Distinguished between survivors and non-survivors. Could not predict the need of hospital admission for patients admitted to the ED with pyelonephritis
Brain natriuretic peptide (BNP) [568, 614-629]	Secreted by ventricular distention [613]	Distinguished between survivors and non-survivors. Fast decline of BNP may indicate response to treatment. Comparable predictive value with PC. Early sepsis diagnosis in trauma patients
Angiotensin converting enzyme (ACE) [630-636]	Localized to the luminal surface of pulmonary endothelial cells; it converts angiotensin I and activates bradykinin- Indicative of pulmonary endothelial cell dysfunction [633]	Lower in patients with septicemia and pneumonia compared to non-septic critically ill patients. It has no prognostic value
Endothelin-1, preproendothelin-1 [561, 637-641]	Peptide with vasoconstrictor and inotropic activities secreted by failing cardiac cells or liver endothelial cells [642]	Diagnosis of heart/liver dysfunction. In combination with pro-ADM can be used as predictor of death. Low diagnostic value in infants
Club cell secretory protein (CC)-16 [643]*	Protein secreted by bronchiolar club cells [644]	Identification of ARDS development
Chromogranin A [645, 646]*	A glycoprotein co-released with catecholamines from the adrenal medulla and peripheral nerve endings with vasodilatory activity. Indicative of adrenergic	Prediction of septic shock development

	activation and cardio-vascular dysfunction [647]	
Glial fibrillary acidic protein (GFAP) [648]	Secreted from activate astrocyte and is related to reactive gliosis [649]	Diagnosis of septic encephalopathy in children
Neuron specific enolase (NSE) [650-652]	Gamma isomer of enolase, a cytoplasmic enzyme of glycolysis located predominantly in neurons [653]	Diagnosis of septic encephalopathy. Distinguished between survivors and non-survivors
Neurofilament chains (plasma) [654]*	Proteins that are important for the neural cytoskeleton. Biomarkers of neuro-axonal injury [655]	Prediction of poor cerebral function outcome in sepsis survivors
Surfactant protein [643, 656, 657]*	Complex of lipids and proteins, functions to keep alveoli from collapsing at expiration. Serum concentration increase indicator of lung parenchymal destruction [658]	Early diagnosis of ARDS in septic patients
Parkinson disease 7 (PRK7) [659] [664]*	Multifunctional peptide involved in antioxidant defense of cells [660]	Early prediction of ARDS development in patients with sepsis or septic shock
Troponin-I [661-672]	3-unit complex located on actin filament, essential for cardiac muscle contraction [673]	Distinguished between survivors and non-survivors
Neutrophil gelatinase associated lipocalin (NGAL) [618, 674-686]*	Belongs to the lipocalin superfamily. Lipocalin secreted by activated neutrophils and epithelial cells [687]	Higher in septic patients with acute kidney injury compared to other septic patients. Prediction of myocardial dysfunction and fatal outcome. Prediction of renal function recovery. Urine NGAL had

		prognostic value in infants
Heart-type fatty acid-binding protein [688-690]*	Constitutes 5–15 % of the cardiac cells' cytosolic protein pool [691]	Diagnosis of heart dysfunction in sepsis- Distinguished between survivors and non-survivors
(alpha) glutathione S-transferase, urinary [692, 693]	detoxification enzymes electively present in the distal tubule [694]	Higher in septic patients compared to non-septic critically ill patients
Secretoneurin [695, 696]*	33-aminoacid peptide secreted by neuroendocrine cells and myocardium in states of heart failure as compensatory mechanisms. It enters cardiac cells by endocytosis and binds directly to calmodulin improving calcium homeostasis [697]	Levels on day 1 of ICU admission distinguished between survivors and non-survivors in patients with septic shock
MEGX test [698, 699]	Monoethylglycinexylidide measurement; lidocaine metabolite through cytochrome P-450 [700]	Correlation to SAPS II score. Diagnosis of liver dysfunction
Micro-RNA-122 [701-703]*	Micro-RNA specifically secreted from liver cells. It is secreted to plasma after hepatic cell damage [704]	Differentiated sepsis from infection within 24h after ICU admission. Improved the prognostic value of the SOFA score
Noncoding RNA [705, 706]*	Non protein-coding RNA with pro-inflammatory action [707]	Higher in septic patients compared to healthy controls. High levels can distinguish survivors and non-survivors in sepsis
S-100B (beta) [651, 652, 708-710, 710]	Calcium-binding protein released predominantly by astroglial cells after brain injury. Marker of the disruption of the blood	Higher in patients with sepsis encephalopathy compared to other septic patients. Distinguished between survivors and non-survivors

	brain barrier [711]	
C-terminal agrin fragment [712]*	Fragment produced by cleavage of agrin by neurotrypsin cleared via glomerular filtration [713]	Correlation with creatinine. Predicted need for renal replacement therapy
Proenkephalin [714, 715]*	Encephalin precursor expressed in epithelial cells of glomeruli [716]	Diagnosis of acute kidney injury in septic patients. Distinguished between survivors and non-survivors
Urinary human kidney Injury molecule type 1 (KIM-1) [717, 718]*	Cell-surface protein highly up-regulated on the surface of injured kidney epithelial cells [719]	Early diagnosis of acute kidney injury -Persistent elevation in non-survivors
Glutamate/glutamine [720]	Amino acid used by hepatic cells. Indicative of liver dysfunction [721]	Distinguished between survivors and non-survivors
Netrin-1 [718, 722]*	Lamin-like protein highly expressed in tubular epithelial cells after ischemia [723]	Early diagnosis of acute kidney injury

*new biomarker since earlier review

Table S8. Acute phase proteins used as biomarkers in sepsis identified in the literature search (with some selected references)

**Name**	**Main pathophysiologic role**	**Main findings**
Serum amyloid A (SSA) [724-734]	Apolipoprotein secreted by many cells triggered by IL-1, IL-6, and TNFa- ligand to TLR2 [735, 736]	Higher in septic neonates compared to healthy controls. Persistent high levels predict fatal outcome
Group II phospholipase A2 (PLA2-II) [68, 296, 737-742]	Enzyme secreted by different cells (e.g phagocytes and platelets) with bactericidal activity [743]	Higher compared to non-septic critically ill patients. Increased values on admission predicted fatal outcome. Limited diagnostic value in trauma patients
Ceruloplasmin [744, 745]	A copper containing glycoprotein synthesized in the liver increasing as part of acute-phase response; it has pro-inflammatory and anti-inflammatory properties [746]	Higher in septic neonates compared to healthy controls
C-reactive protein (CRP) [55, 63, 234, 285, 363, 552, 747-776]	Protein synthetized in the liver with multiple physiological roles: complement activation, pro and anti-inflammatory effects [777]	Distinguished between survivors and non-survivors
Ferritin [778]	Acute phase protein with a role in modulation host immune activity [779]	Distinguished between survivors and non-survivors, in children with sepsis
Transferrin [780]*	Acute phase protein with long half-life; transferrin sialic acid content indicative of neuraminidase activity [781]	Correlation with sepsis severity

Alpha-1-acid glycoprotein (AGP) [782-786]*	A heavily glycosylated protein; decreases the activity of neutrophils, it has an antiagreggant effect and possibly pro-inflammatory and anti-inflammatory effects [787]	Distinguished between survivors and non-survivors. Higher in patients with sepsis compared to non-septic critically ill patients
Hepcidin [788, 789]	Peptide that inhibits iron absorption in the small intestine [790]	Higher in neonates with sepsis compared to those without
Apolipoprotein C2 [791]	Downregulation in sepsis [792]	Higher in septic neonates compared to non-septic neonates
α-2 macroglobulin [793-796]	Binding protein, proteinase inhibitor [797]	Diagnosis of sepsis in surgical critically ill patients. Limited prognostic and diagnostic value in neonates
Apolipoprotein E [798, 799]	Increased concentration because of decrease in clearance [800]	Higher in children with bacterial infection compared to children without infection or non-bacterial infection
Lipopolysaccharide-binding protein (LBP) [17, 72, 801-810]	Connects to LPS of Gram (-) bacteria; facilitates the connection of LPS to CD14 [810]	Higher in surgical patients with sepsis than surgical patients with infection. Diagnostic accuracy comparable to CRP. Combination with other biomarkers (PCT, CRP) did not improve diagnostic efficiency. Serial measurement in patients with sepsis predicted ARDS development and fatal outcome
Procalcitonin (PCT) [55, 63, 120, 234, 285, 323, 362, 363, 505, 552, 618, 748-776, 806, 811-870]	Prehormone of calcitonin- Its secretion is stimulated mainly by IL-6, IL-1, TNF-a [871]	Higher in patients with septicemia compared to other critically ill patients
Pentraxin 3 (PTX3) [136, 872-885]	Secreted by macrophages and dendritic cells, can facilitate pathogen	Distinguished between survivors and non-survivors. Higher in septic patients compared to healthy

	recognition and removal [886]	controls. Improved the predictive value of PCT. Better prognostic value in patients with VAP
Complement factors C5a,C3a [887-890]	Fragment of C5 factor with chemoattractant components [891]	Higher in septic patients compared to healthy volunteers-Inverse relationship with SOFA score
HSP 70 [892-896]	Heat shock protein released by liver and spleen as acute phase reactant and participate in activation and maturation of DCs and protection of cells (antiapoptic effect) [897]	Higher in patients with septic shock compared to healthy controls
Pancreatic stone protein [362, 850, 898-904]	Acute phase protein secreted from pancreas stimulated by TNF-α, IL-6, IF-γ [905]	Distinguished between survivors and non-survivors in patients with peritonitis. Higher in infected compared to non-infected patients in ED and after cardiac operation
Fetuin-A [906]*	Acute phase protein that is counter-regulated by pro-inflammatory cytokines. [907]	Lower in non-survivors with sepsis. Negative correlation with APACHE II score

*new biomarker since earlier review

Table S9 Diverse sepsis biomarkers identified in the literature search (with some selected references)

Name	Main pathophysiologic role	Main findings
Apolipoprotein CI [908, 909]	HDL apolipoprotein that induces inflammatory response via its connection to LPS [910]	Distinguished between survivors and non-survivors
Selenoproteins [911-913]	Selenium rich protein with high antioxidant capacity [914]	Lower in septic patients compared to healthy volunteers. Septic shock prediction
Copeptin [915-921]	Reflects AVP levels as released in equimolar ratio with AVP [922]	Distinguished between survivors and non-survivors- Higher in patients with respiratory infection compared to non-septic ED patients. Not efficient in pediatric patients
Gc-globulin [923]	Protein of extracellular actin-scavenger system [924]	Prediction of development of respiratory failure and sepsis in trauma patients
Cold inducible RNA binding protein [925]*	Damage-associated protein secreted by damaged tissues that stimulates pro-inflammatory cytokine release. Contribution to endothelial dysfunction [926]	Distinguished between survivors and non-survivors
Coenzyme Q10 [927]*	Lipid founded in mitochondria, its role is an electron shuttle of the respiratory chain cofactor of dehydrogenases and an	Lower in septic patients compared to healthy volunteers

	antioxidant factor [928]	
F2-isoprostanes [929, 930]	Products of non-enzymatic lipid peroxidation [931]	Prediction of multiorgan failure development
Homocysteine [932]*	Secreted by activated mononuclear cells [933]	Distinguished between survivors and non-survivors
Malondialdehyde [934]*	Product of lipid peroxidation [933]	Distinguished between survivors and non-survivors
Uric acid [935-937]	Oxidative product of purine metabolism with antioxidant activity and nephrotoxic effects [938, 939]	Elevated uric acid at admission related to higher mortality
Myoglobin [940]*	Heterodimer composed of a peptide chain and heme found in the cytoplasm of myocardial and skeletal muscle cells. Rapid release into the blood circulation in case of injury [941]	Positive correlation with SOFA score 24h after ICU admission
Urinary 8-hydroxy-2'-deoxyguanosine (8-OhdG) [942]	Nucleoside produced by oxidative DNA damage [943]	Distinguished between survivors and non-survivors
Urinary bilirubin oxidative metabolites (BOMs) [944]	Bilirubin metabolites excreted in urine indicative of antioxidant activity of bilirubin [945]	Increased in parallel with disease severity
Cholesterol (total, LDL-C, HDL-C) [946-952]	Neutralizes bacterial endotoxin, enhances the antigen-presenting function of monocytes and principal precursor for steroid biosynthesis	Distinguished between survivors and non-survivors

	[952]	
Phosphatidylethanolamine(18:0/20:4) [953]*	Aminophospholipid localized in the inner leaflet of plasma membrane. It is released to plasma after cellular blebbing [954]	Increased on day 1 in neutropenic patients with sepsis or septic shock
Diiodotyrosine [955]	Phagocytosing leukocytes metabolize T_4 to diiodotyrosine [956]	Increased in parallel with disease severity
Erythropoietin (EPO) [957]	Glycoprotein whose production is stimulated by tissue hypoxia [958]	Distinguished between survivors and non-survivors
Leptin [959-963]	Leptin is secreted by mononuclear cells ; it contributes in mononuclear activation, stimulates the production of pro-inflammatory cytokines and enhances the production of Th1 cells [964]	Higher in septic patients compared to non-septic critically ill patients
Adiponectin [965]*	Hormone secreted by adipocytes; via anti-inflammatory mechanism it limits leukocyte chemotaxis/infiltration and endothelial cell activation [966]	Distinguished between survivors and non-survivors
Renin [957]	Enzyme secreted by renal juxtaglomerular cells in response to sympathetic nervous system activation and decrease glomerular filtration [967]	Increased in parallel with disease severity

G-CSF and GM-CSF [968-972]	Hematopoietic growth factor central role functional activation of immature and mature neutrophils [973]	Distinguished between survivors and non-survivors
Procollagen III [974, 975]*	Component of Type III collagen; reflects collagen synthesis and tissue reparative process [976]	Higher in patients with severe sepsis compared to healthy volunteers. Predicted severe sepsis development
Albumin [977-981]	Indicative of increased vascular permeability [982]	Distinguished between survivors and non-survivors in patients with community-acquired bloodstream infection. Lactate/albumin was superior prognostic index than single lactate measurement for predicting 28-day mortality
Annexin V binding leukocytes [983]	Protein that shows high affinity for the surface presented phosphatidylserine; indicative of apoptosis [984]	Lower in surgical patients with sepsis compared to healthy volunteers
Anti-endotoxin core antibodies (EndoCab) [985, 986]	Indicative of endotoxin level and evaluation of endogenous immunity against endotoxin [987, 988]	Distinguished between survivors and non-survivors
Bactericidal/permeability increasing protein (BPI) [741]*	Cationic protein stored in polymorphonuclear leukocytes with bactericidal activity for Gram (–) bacteria; inhibit the numerous biological effects of LPS [989]	Higher in septic patients compared to non-septic patients admitted to emergency department
Cell-free DNA (cfDNA) [990-1000]	Coming from dead cells of	Distinguished between

	damaged tissue; indicative of tissue damage due to inflammation [1001]	survivors and non-survivors. Early sepsis diagnosis in patients with inflammatory reaction. Worse performance compared to PCT. Normal concentration can exclude sepsis in febrile patients
Ceramide [1002]	Product coming from hydrolysis of sphingomyelin by acid sphingomyelinase; acid sphingomyelinase is activated in response to stimulation with LPS, TNFα [1003]	Prediction of multiorgan failure development in sepsis
Circulating nucleosomes [1004-1007]	Complexes formed by DNA and histone proteins released by apoptotic cell death [1008]	Prediction of MOF development. Higher compared to non-septic critically ill patients. Markedly elevated in septic patients with severe coagulopathy
Dendritic cell [1009, 1010]	Cells that induce primary immune leading to T-cell activation [1011]	Correlation to SAPS and SOFA score
Factor VII activating protein, FSAP [1012, 1013]*	Plasma serine protease is activated by dead cells and removes nucleosomes from apoptotic cells [1014]	Distinguished between survivors and non-survivors
Granzyme A [1015]*	Serine-protease present in the intracytoplasmatic granules increased in agreement with NK activation [1014]	Distinguished between survivors and non-survivors. Lower in septic burned patients compared to non-septic
H-ficolin [1016]*	Protein related to activation of the lectin	Low in neutropenic children with septicemia

	complement pathway-indicative of innate immunity [1017]	
Catalytic antibodies [1018]*	Antibodies with enzyme-like properties [1019]	Distinguished between survivors and non-survivors
Inter-alpha inhibitor proteins [1020, 1021]	Protein that binds bikunin (a pro-inflammatory protein active when it is free)-Regulator of free bikunin levels [1022]	Lower in neonates with septicemia compared to others
Lactotransferrin (LTF) [1023-1025]	Protein secreted by neutrophils that limits microbial growth [1026]	Response to antibiotic treatment
Nociceptin/orphanin FQ (N/OFQ) [1027]	Opioid secreted by polymorphonuclear cells and monocytes with pro-inflammatory activity; increases capillary leakage and leads to immune cell chemotaxis [1028]	Distinguished between survivors and non-survivors
Peptidoglycan [1029]	Cell wall component [1030]	Diagnosis of bacteremia
Plasmalogen [1031]*	Phospholipid that protects cell membranes from oxidative stress [1032]	Lower in septic patients compared to healthy volunteers
Resistin (RETN) [1033-1038]	Cysteine-rich protein released by neutrophils with pro-inflammatory activity. Its pro-inflammatory role is related to the NF-kappa B signaling pathway [1039, 1040]	Distinguished between survivors and non-survivors-Higher in septic patients compared to non-septic

Selenium binding protein-1 [1041]*	Increased inversely with selenium concentration; inverse relation with glutathione peroxidases [1042]	Distinguished between survivors and non-survivors
Trefoil factor 2 [1043]*	Structural peptide secreted by predominantly by gastric mucous neck cells; secretion upregulated in gut injuries [1044]	Predicted MOF development
YKL-40 [1045]*	Glycoprotein consisting matrix protein of specific granules secreted early by activated neutrophils and macrophage [1046]	Distinguished between survivors and non-survivors
Bcl-2 [1047]	Protein that prevents dendritic cell apoptosis [1047, 1048]	Distinguished between survivors and non-survivors
Caspase-1 [1049]	Enzyme that converts proIL-1β to IL-1β and related to macrophage apoptosis [1050]	Higher in septic patients compared to healthy controls
Dipeptidylpeptidase activity [1051]	Cell surface protease that selectively removes N-terminal dipeptide from peptides [1052]	Decreased in septic patients compared to healthy controls
Gelsolin [1053, 1054]	Actin binding protein [1055]	Distinguished between survivors and non-survivors. Lower in septic patients compared to non-septic critically ill patients
Elastase [1056-1058]	Protein released by neutrophils to form neutrophilic extracellular	Response to antibiotic therapy in patients with osteomyelitis

	traps [1059]	
Growth arrest specific protein (Gas 6) [1060, 1061]	A vitamin K dependent protein released mainly by leukocytes and epithelial cells; participates in cell survival (neutrophils and epithelial cells) [1062]	Higher in septic patients compared to patients with infection but without sepsis
Elastase-alpha-1-proteinase inhibitor complex [1056, 1063]	A1-proteinase inhibitor inactivates elastase by forming complexes with it [1064]	Higher in neutropenic children with septicemia compared to others
Hyaluronan [1065, 1066]	Connective tissue polysaccharide; serum levels are regulated by production and influx via lymph and clearance by the liver [1067]	Distinguished between survivors and non-survivors
Intracellular nitric oxide in leukocyte [447, 1068]	Indicative of total NO concentration [447]	Negative correlation with SOFA score
Lysophosphatidylcholine [837, 1069, 1070]*	Endogenous lysophospholipid related to macrophage 1 stimulation [1071]	Serial measurement distinguished survivors and non-survivors
Matrix-metalloproteinases [454, 456, 457, 685, 1072, 1073]	Zn^{2+} dependent endopeptidase secreted by neutrophils and involved in extracellular matrix degradation [1074]	Limited predictive value for death. No diagnostic value
NF-kB activity [1075]	Transcriptional regulatory factor that plays fundamental role in activation of the cytokine cascade and production of other pro-inflammatory	Distinguished between survivors and non-survivors

	mediators [1076]	
Ischemia-modified albumin [1077-1079] *	Produced after modification of the N-terminus albumin by reactive oxygen species [1080]	Higher in septic patients compared to healthy controls
Sphingomyelinase [1081]	Hydrolyzes the ubiquitous sphingophospholipid sphingomyelin to ceramide which is associated to apoptosis [1082]	Distinguished between survivors and non-survivors
Sulfite [1083]	Inorganic substance released by activated llb neutrophils [1084]	Response to treatment
Citrulline [1085-1088]*	Amino acid that is synthesized solely by the gut. An intermediate amino acid in urea cycle for L-arginine synthesize and NO production [1089]	Decreased levels predict development of ARDS in patients with sepsis. Decreased levels on day 1 in patients with neutropenic fever and bacteremia. Low levels of citrulline in catheter-related bloodstream infections
Histidine-rich glycoprotein [1090, 1091]*	Multidomain protein produced by liver. Maintains neutrophils and vascular endothelial cells in an inactive state. Decreases rapidly in sepsis [1091]	Decreased levels could differentiate diagnosis of sepsis versus patients with SIRS. Low levels on day 1 were associated with higher mortality
Calprotectin [1092-1097]*	Protein of cytosolic fraction of neutrophils released in circulation after their activation [1098]	Prediction of bacterial infection within 24h of admission. Better diagnostic value than PCT. Differential diagnosis bacterial sepsis to viral infection
Uncoupling protein 2 (UCP2) [1099]*	Mitochondrial protein	Higher compared to healthy

	that cause a decrease in ATP synthesis [1100]	controls. Correlation with the severity of sepsis. Decreased after effective antibiotic treatment initiation
Soluble CD154 (CD40L) [1101]*	Expressed on a variety of cells. Ligand to CD40 receptor related to T-cells stimulation [337]	Distinguished between survivors and non-survivors. Higher compared to healthy volunteers
P-selectin [385, 1102-1107]	Glycoprotein that is stored in endothelial cells or in platelets. It is associated with leukocyte recruitment [1108]	Higher in critically ill patients that developed an infection after their admission compared to others that never developed infection. High levels related to fatal outcome
Anadamide [1109]	Endocannabinoid that modulates NO production [1110]	Increased in sepsis compared with healthy controls
2-arachidonoylglycerol [1111]	Endocannabinoid with anti-inflammatory activity [1112]	Increased in sepsis compared with healthy controls
Carbamoyl phosphate synthase [1113, 1114]	Protein primarily localized in mitochondria [1114]	Increased in sepsis compared with healthy controls
Terminal complement complex [1115]	Product of final common reaction sequence of complement activation [1115]	Decreased in plasma in sepsis compared to healthy controls
Ghrelin [959, 1116]	Peptide produced by the stomach with anti-inflammatory activity, reestablishing the proliferation of CD4 T cells [1117]	Increased in sepsis compared with healthy controls
H2S [1118, 1119]	H2S is generated from cysteine metabolism, a reaction catalyzed by	Detection in expired gas of children. Higher in septic than non-septic mechanically

	phosphotyrosine-5’-phosphate-dependent enzymes. Different biological effects including pro-inflammatory, anti-inflammatory and vasodilatory [1120]	ventilated children and neonates
Serum lysozyme (enzyme activity) [1121]	Protein that is released from leukocytes and macrophages, a mediator of myocardial depression and vasodilation. It also deposits in the systemic vasculature causing acute organ failure [1122]	Higher in septic transplant recipients patients compared to those with graft rejection without any infection.
Neurotensin [1123]	Neuropeptide that induces hypotension [1124]	Increased in sepsis and related to death
PIGF [1125]	A member of the vascular endothelial growth factor family with pleiotropic activities. It counteracts the production of VEGF protective hosts from heart dysfunction and vascular leakage [1126]	Increased in sepsis and related to death
Selenium [1127, 1128]	An essential trace metal, which has direct antioxidant activity by incorporating into selenoproteins. Low levels are associated with increased oxidative stress [1129]	Low levels predicted fatal outcome
Myeloperoxidase [1130, 1131]*	Enzyme secreted by activated neutrophils	Increased in sepsis compared to non-septic critically ill

	[1132]	patients
Tenascin-C [1133]*	Glycoprotein which increases pro-inflammatory cytokines secretion by macrophages inhibiting at the same time the synthesis of anti-inflammatory cytokines [1133]	Increased in sepsis compared to non-septic critically ill patients. Association with sepsis severity and fatal outcome
Fibroblast growth factor-21 [1134]*	Amino acid predominantly produced by the liver. It has an anti-inflammatory effect [1135]	Distinguished between survivors and non-survivors
Developmental endothelial locus-1 [1136]*	Endogenous inhibitor of leukocyte adhesion to endothelium [1137]	Higher in septic patients compared to healthy control. Higher in more severely ill septic patients
Apoptosis inhibitor of macrophage (AIM) [1138]*	IgM-binding protein which acts as recognition molecule. It also has apoptosis inhibitor function for macrophages, T-cells and NKT cells [1139]	Higher in septic patients compared to healthy control. Distinguished between survivors and non-survivors at ICU admission
Acylcarnitine [1140]*	Acetylated form of L-carnitine [1140]	Distinguished between survivors and non-survivors
C1q/tumor necrosis factor-like protein 3 (CTRP3) [1141]*	Adipokine with metabolic (inhibition of gluconeogenesis, lowering glucose levels) and anti-inflammatory effects [1142]	Lower in critically ill patients with sepsis compared to critically ill patients without sepsis. Correlation with disease severity
Delta-like canonical Notch ligand 1 (DLL1) [1143]*	Monocyte transmembrane protein, upregulated during	Higher in septic patients than in patients after major surgery or trauma patients without

	infection and plays a role in monocyte accumulation and amplification of pro-inflammatory cytokine response [1144]	infection or healthy controls

*new biomarker since earlier review

Table S10. QUADAS-2 score [1145] for quality assessment for the studies that included >300 patients where ROC curve analysis was used

	Patient selection		Index test		Reference standard		Flow and Timing
	Risk of Bias	Applicability	Risk of Bias	Applicability	Risk of Bias	Applicability	Risk of bias
**Diagnosis of Sepsis**							
Hanna et al 2015 (interleukin-27) [120]	Low	Low	Unclear	Low	Low	Low	Low
Chaaban et al 2019 (inter-alpha inhibitor proteins) [1021]	Low	High	High	Low	High	Low	Unclear
Dimoula et al 2014 (CD64) [298]	Low	Low	High	Low	Low	Low	Low
Sakr et al 2008 (lipopolysaccharide-binding protein) [810]	Low	Low	High	Low	Low	Low	Low
Nakamura et al 2019 (presepsin) [867]	High	Low	High	Low	Low	Low	Unclear
Henning et al 2019 (IL-6) [82]	Low	Low	High	Low	Low	Low	Low
**Survival prognosis**							
Giamarellos-Bourboulis et al 2012 (uPAR) [239]	Low	Low	High	Low	Low	Low	Low

Novotny et al 2006 (IL-12) [101]	Unclear	High	High	Low	Low	Low	Low
Chen et al 2009 (BNP) [615]	Unclear	Low	High	Low	Low	Low	Unclear
Chen et al 2013 (adrenomedullin) [550]	Low	Low	High	Low	Low	Low	Low
Saeed et al 2019 (pro-adrenomedullin) [567]	Low	Low	High	Low	Low	Low	Low
Rodelo et al 2012 (D-dimer) [402]	Low	Low	High	Low	Low	Low	Low
Kornblit et al 2013 (YKL-40) [1045]	Low	Low	High	Low	Low	Low	Low
Dimoula et al 2014 (CD64) [298]	Low	Low	High	Low	Low	Low	Low
Ranniko et al 2018 (cell-free DNA) [1146]	Unclear	Low	High	Low	Low	Low	Low

IL: interleukin

References

1. Ghani RA, Zainudin S, Ctkong N, Rahman AF, Wafa SR, Mohamad M et al. Serum IL-6 and IL-1-ra with sequential organ failure assessment scores in septic patients receiving high-volume haemofiltration and continuous venovenous haemofiltration. Nephrology (Carlton). 2006;11:386-93.

2. Nguyen HB, Loomba M, Yang JJ, Jacobsen G, Shah K, Otero RM et al. Early lactate clearance is associated with biomarkers of inflammation, coagulation, apoptosis, organ dysfunction and mortality in severe sepsis and septic shock. J Inflamm (Lond). 2010;7:6.

3. Schmidhammer R, Wassermann E, Germann P, Redl H, Ullrich R. Infusion of increasing doses of endotoxin induces progressive acute lung injury but prevents early pulmonary hypertension in pigs. Shock. 2006;25:389-94.

4. Hynninen M, Valtonen M, Markkanen H, Vaara M, Kuusela P, Jousela I et al. Interleukin 1 receptor antagonist and E-selectin concentrations: a comparison in patients with severe acute pancreatitis and severe sepsis. J Crit Care. 1999;14:63-8.

5. Kuster H, Weiss M, Willeitner AE, Detlefsen S, Jeremias I, Zbojan J et al. Interleukin-1 receptor antagonist and interleukin-6 for early diagnosis of neonatal sepsis 2 days before clinical manifestation. Lancet. 1998;352:1271-7.

6. Arend WP, Malyak M, Guthridge CJ, Gabay C. Interleukin-1 receptor antagonist: role in biology. Annu Rev Immunol. 1998;16:27-55.

7. Intke C, Korpelainen S, Hamalainen S, Vanska M, Koivula I, Jantunen E et al. Interleukin-1 receptor antagonist as a biomarker of sepsis in neutropenic haematological patients. Eur J Haematol. 2018;101:691-698

8. Yu H, Qi Z, Zhao L, Shao R, Fang Y, Li C. Prognostic value of dynamic monitoring of cellular immunity and HMGB1 in severe sepsis: Delayed chronic inflammation may be the leading cause of death in late severe sepsis. Clin Lab. 2016;62:2379-85.

9. Zheng YJ, Xu WP, Ding G, Gao YH, Wang HR, Pan SM. Expression of HMGB1 in septic serum induces vascular endothelial hyperpermeability. Mol Med Rep. 2016;13:513-21.

10. Wang XW, Karki A, Zhao XJ, Xiang XY, Lu ZQ. High plasma levels of high mobility group box 1 is associated with the risk of sepsis in severe blunt chest trauma patients: a prospective cohort study. J Cardiothorac Surg. 2014;9:133.

11. Pavare J, Grope I, Kalnins I, Gardovska D. High-mobility group box-1 protein, lipopolysaccharide-binding protein, interleukin-6 and C-reactive protein in children with community acquired infections and bacteraemia: a prospective study. BMC Infect Dis. 2010;10:28.

12. Ueno T, Ikeda T, Ikeda K, Taniuchi H, Suda S, Yeung MY et al. HMGB-1 as a useful prognostic biomarker in sepsis-induced organ failure in patients undergoing PMX-DHP. J Surg Res. 2011;171:183-90.

13. Gamez-Diaz LY, Enriquez LE, Matute JD, Velasquez S, Gomez ID, Toro F et al. Diagnostic accuracy of HMGB-1, sTREM-1, and CD64 as markers of sepsis in patients recently admitted to the emergency department. Acad Emerg Med. 2011;18:807-15.

14. Velasquez S, Matute JD, Gamez LY, Enriquez LE, Gomez ID, Toro F et al. Characterization of nCD64 expression in neutrophils and levels of s-TREM-1 and HMGB-1 in patients with suspected infection admitted in an emergency department. Biomedica. 2013;33:643-52.

15. Karlsson S, Pettila V, Tenhunen J, Laru-Sompa R, Hynninen M, Ruokonen E. HMGB1 as a predictor of organ dysfunction and outcome in patients with severe sepsis. Intensive Care Med. 2008;34:1046-53.

16. Gibot S, Massin F, Cravoisy A, Barraud D, Nace L, Levy B et al. High-mobility group box 1 protein plasma concentrations during septic shock. Intensive Care Med. 2007;33:1347-53.

17. Gaini S, Koldkjaer OG, Moller HJ, Pedersen C, Pedersen SS. A comparison of high-mobility group-box 1 protein, lipopolysaccharide-binding protein and procalcitonin in severe community-acquired infections and bacteraemia: a prospective study. Crit Care. 2007;11:R76.

18. Gaini S, Pedersen SS, Koldkjaer OG, Pedersen C, Moller HJ. High mobility group box-1 protein in patients with suspected community-acquired infections and sepsis: a prospective study. Crit Care. 2007;11:R32.

19. Sunden-Cullberg J, Norrby-Teglund A, Rouhiainen A, Rauvala H, Herman G, Tracey KJ et al. Persistent elevation of high mobility group box-1 protein (HMGB1) in patients with severe sepsis and septic shock. Crit Care Med. 2005;33:564-73.

20. Shao YM, Yao HG, Liang XZ, Xia YH. [Relation between level of expression of high mobility group protein B1 in hepatic tissue with the severity and prognosis of sepsis in rat]. Zhongguo Wei Zhong Bing Ji Jiu Yi Xue. 2006;18:668-72.

21. Wang H, Bloom O, Zhang M, Vishnubhakat JM, Ombrellino M, Che J et al. HMG-1 as a late mediator of endotoxin lethality in mice. Science. 1999;285:248-51.

22. Hall MW, Geyer SM, Guo CY, Panoskaltsis-Mortari A, Jouvet P, Ferdinands J et al. Innate immune function and mortality in critically ill children with influenza: a multicenter study. Crit Care Med. 2013;41:224-36.

23. Drewry AM, Ablordeppey EA, Murray ET, Beiter ER, Walton AH, Hall MW et al. Comparison of monocyte human leukocyte antigen-DR expression and stimulated tumor necrosis factor alpha production as outcome predictors in severe sepsis: a prospective observational study. Crit Care. 2016;20:334.

24. van Vught LA, Wiewel MA, Hoogendijk AJ, Scicluna BP, Belkasim-Bohoudi H, Horn J et al. Reduced responsiveness of blood leukocytes to lipopolysaccharide does not predict nosocomial infections in critically ill patients. Shock. 2015;44:110-4.

25. Badiu DC, Paunescu V, Aungurenci A, Pasarica D. Proinflammatory cytokines in peritonitis. J Med Life. 2011;4:158-62.

26. Calandra T, Baumgartner JD, Grau GE, Wu MM, Lambert PH, Schellekens J et al. Prognostic values of tumor necrosis factor/cachectin, interleukin-1, interferon-alpha, and interferon-gamma in the serum of patients with septic shock. Swiss-Dutch J5 Immunoglobulin Study Group. J Infect Dis. 1990;161:982-7.

27. Riche F, Panis Y, Laisne MJ, Briard C, Cholley B, Bernard-Poenaru O et al. High tumor necrosis factor serum level is associated with increased survival in patients with abdominal septic shock: a prospective study in 59 patients. Surgery. 1996;120:801-7.

28. Ferro TJ, Gertzberg N, Selden L, Neumann P, Johnson A. Endothelial barrier dysfunction and p42 oxidation induced by TNF-alpha are mediated by nitric oxide. Am J Physiol. 1997;272:L979-L988.

29. Roderburg C, Benz F, Schuller F, Pombeiro I, Hippe HJ, Frey N et al. Serum levels of TNF receptor ligands are dysregulated in sepsis and predict mortality in critically ill patients. PLoS One. 2016;11:e0153765.

30. Burkly LC, Michaelson JS, Hahm K, Jakubowski A, Zheng TS. TWEAKing tissue remodeling by a multifunctional cytokine: role of TWEAK/Fn14 pathway in health and disease. Cytokine. 2007;40:1-16.

31. Lendak DF, Mihajlovic DM, Novakov-Mikic AS, Boban JM, Ubavic M, Brkic SV. APRIL and sTACI could be predictors of multiorgan dysfunction syndrome in sepsis. Virulence. 2018;9:946-53.

32. Roderburg C, Koch A, Tacke F, Nieuwenhuijsen L, Bruensing J, Vargas Cardenas D et al. Serum concentrations of A Proliferation-Inducing Ligand (APRIL) are elevated in sepsis and predict mortality in critically ill patients. J Crit Care. 2013;28:882-11.

33. Planelles L, Medema JP, Hahne M, Hardenberg G. The expanding role of APRIL in cancer and immunity. Curr Mol Med. 2008;8:829-44.

34. Ayazi P, Mahyar A, Daneshi MM, Jahanihashemi H, Esmailzadehha N, Mosaferirad N. Comparison of serum IL-1beta and C reactive protein levels in early diagnosis and management of neonatal sepsis. Infez Med. 2014;22:296-301.

35. de Bont ES, Martens A, van Raan J, Samson G, Fetter WP, Okken A et al. Tumor necrosis factor-alpha, interleukin-1 beta, and interleukin-6 plasma levels in neonatal sepsis. Pediatr Res. 1993;33:380-3.

36. Strait RT, Kelly KJ, Kurup VP. Tumor necrosis factor-alpha, interleukin-1 beta, and interleukin-6 levels in febrile, young children with and without occult bacteremia. Pediatrics. 1999;104:1321-6.

37. Strait RT, Ruddy RM, Friedland LR, Duncan KM, Wilmott RW. A pilot study of the predictive value of plasma tumor necrosis factor alpha and interleukin 1 beta for Streptococcus pneumoniae bacteremia in febrile children. Acad Emerg Med. 1997;4:44-51.

38. Ozdemir A, Oygur N, Gultekin M, Coskun M, Yegin O. Neonatal tumor necrosis factor, interleukin-1 alpha, interleukin-1 beta, and interleukin-6 response to infection. Am J Perinatol. 1994;11:282-5.

39. Kelly JL, O'Sullivan C, O'Riordain M, O'Riordain D, Lyons A, Doherty J et al. Is circulating endotoxin the trigger for the systemic inflammatory response syndrome seen after injury? Ann Surg. 1997;225:530-41.

40. Murch O, Collin M, Sepodes B, Foster SJ, Mota-Filipe H, Thiemermann C. Lysophosphatidylcholine reduces the organ injury and dysfunction in rodent models of gram-negative and gram-positive shock. Br J Pharmacol. 2006;148:769-77.

41. Kurt AN, Aygun AD, Godekmerdan A, Kurt A, Dogan Y, Yilmaz E. Serum IL-1beta, IL-6, IL-8, and TNF-alpha levels in early diagnosis and management of neonatal sepsis. Mediators Inflamm. 2007;2007:31397.

42. Weber A, Wasiliew P, Kracht M. Interleukin-1 (IL-1) pathway. Sci Signal. 2010;3:cm1.

43. BalcI C, Sungurtekin H, Gurses E, Sungurtekin U, Kaptanoglu B. Usefulness of procalcitonin for diagnosis of sepsis in the intensive care unit. Crit Care. 2003;7:85-90.

44. Aziz M, Yang WL, Matsuo S, Sharma A, Zhou M, Wang P. Upregulation of GRAIL is associated with impaired CD4 T cell proliferation in sepsis. J Immunol. 2014;192:2305-14.

45. Wu HP, Wu CL, Chen CK, Chung K, Tseng JC, Liu YC et al. The interleukin-4 expression in patients with severe sepsis. J Crit Care. 2008;23:519-24.

46. Hynninen M, Pettila V, Takkunen O, Orko R, Jansson SE, Kuusela P et al. Predictive value of monocyte histocompatibility leukocyte antigen-DR expression and plasma interleukin-4 and -10 levels in critically ill patients with sepsis. Shock. 2003;20:1-4.

47. DiPiro JT, Howdieshell TR, Goddard JK, Callaway DB, Hamilton RG, Mansberger AR, Jr. Association of interleukin-4 plasma levels with traumatic injury and clinical course. Arch Surg. 1995;130:1159-62.

48. Chomarat P, Banchereau J. An update on interleukin-4 and its receptor. Eur Cytokine Netw. 1997;8:333-44.

49. Lee WJ, Woo SH, Kim DH, Seol SH, Park SK, Choi SP et al. Are prognostic scores and biomarkers such as procalcitonin the appropriate prognostic precursors for elderly patients with sepsis in the emergency department? Aging Clin Exp Res. 2016;28:917-24.

50. Aquino VM, Cost C, Gomez A, Bowers DC, Ramilo O, Ahmad N et al. Predictive value of interleukin-5 and monocyte chemotactic protein-1 for bacteremia in children with febrile neutropenia. J Pediatr Hematol Oncol. 2012;34:e241-e245.

51. Linch SN, Danielson ET, Kelly AM, Tamakawa RA, Lee JJ, Gold JA. Interleukin 5 is protective during sepsis in an eosinophil-independent manner. Am J Respir Crit Care Med. 2012;186:246-54.

52. Ricarte-Bratti JP, Brizuela NY, Jaime-Albarran N, Montrull HL. IL-6, MMP 3 and prognosis in previously healthy sepsis patients. Rev Fac Cien Med Univ Nac Cordoba. 2017;74:99-106.

53. Nakstad B, Sonerud T, Solevag AL. Early detection of neonatal group B streptococcus sepsis and the possible diagnostic utility of IL-6, IL-8, and CD11b in a human umbilical cord blood in vitro model. Infect Drug Resist. 2016;9:171-9.

54. Tranca S, Oever JT, Ciuce C, Netea M, Slavcovici A, Petrisor C et al. sTREM-1, sIL-2Ralpha, and IL-6, but not sCD163, might predict sepsis in polytrauma patients: a prospective cohort study. Eur J Trauma Emerg Surg. 2017;43:363-70.

55. Noor MK, Shahidullah M, Mutanabbi M, Barua C, Mannan MA, Afroza S. Comparison between CRP and IL-6 as early markers of neonatal sepsis. Mymensingh Med J. 2008;17:S72-S76.

56. Prinsen JH, Baranski E, Posch H, Tober K, Gerstmeyer A. Interleukin-6 as diagnostic marker for neonatal sepsis: determination of Access IL-6 cutoff for newborns. Clin Lab. 2008;54:179-83.

57. Veleminsky M, Jr., Stransky P, Veleminsky M, Sr., Tosner J. Relationship of IL-6, IL-8, TNF and sICAM-1 levels to PROM, pPROM, and the risk of early-onset neonatal sepsis. Neuro Endocrinol Lett. 2008;29:303-11.

58. Chawla LS, Seneff MG, Nelson DR, Williams M, Levy H, Kimmel PL et al. Elevated plasma concentrations of IL-6 and elevated APACHE II score predict acute kidney injury in patients with severe sepsis. Clin J Am Soc Nephrol. 2007;2:22-30.

59. de Bont ES, Martens A, van Raan J, Samson G, Fetter WP, Okken A et al. Diagnostic value of plasma levels of tumor necrosis factor alpha (TNF alpha) and interleukin-6 (IL-6) in newborns with sepsis. Acta Paediatr. 1994;83:696-9.

60. Ma L, Zhang H, Yin YL, Guo WZ, Ma YQ, Wang YB et al. Role of interleukin-6 to differentiate sepsis from non-infectious systemic inflammatory response syndrome. Cytokine. 2016;88:126-35.

61. Plesko M, Suvada J, Makohusova M, Waczulikova I, Behulova D, Vasilenkova A et al. The role of CRP, PCT, IL-6 and presepsin in early diagnosis of bacterial infectious complications in paediatric haemato-oncological patients. Neoplasma. 2016;63:752-60.

62. Lin S, Huang Z, Wang M, Weng Z, Zeng D, Zhang Y et al. Interleukin-6 as an early diagnostic marker for bacterial sepsis in patients with liver cirrhosis. J Crit Care. 2015;30:732-8.

63. Neunhoeffer F, Plinke S, Renk H, Hofbeck M, Fuchs J, Kumpf M et al. Serum concentrations of interleukin-6, procalcitonin, and C-reactive protein: Discrimination of septical complications and systemic inflammatory response syndrome after pediatric surgery. Eur J Pediatr Surg. 2016;26:180-5.

64. Naffaa M, Makhoul BF, Tobia A, Kaplan M, Aronson D, Saliba W et al. Interleukin-6 at discharge predicts all-cause mortality in patients with sepsis. Am J Emerg Med. 2013;31:1361-4.

65. Jekarl DW, Lee SY, Lee J, Park YJ, Kim Y, Park JH et al. Procalcitonin as a diagnostic marker and IL-6 as a prognostic marker for sepsis. Diagn Microbiol Infect Dis. 2013;75:342-7.

66. Miguel-Bayarri V, Casanoves-Laparra EB, Pallas-Beneyto L, Sancho-Chinesta S, Martin-Osorio LF, Tormo-Calandin C et al. Prognostic value of the biomarkers procalcitonin, interleukin-6 and C-reactive protein in severe sepsis. Med Intensiva. 2012;36:556-62.

67. Urbonas V, Eidukaite A, Tamuliene I. The diagnostic value of interleukin-6 and interleukin-8 for early prediction of bacteremia and sepsis in children with febrile neutropenia and cancer. J Pediatr Hematol Oncol. 2012;34:122-7.

68. Lausevic Z, Vukovic G, Stojimirovic B, Trbojevic-Stankovic J, Resanovic V, Lausevic M. Kinetics of C-reactive protein, interleukin-6 and -10, and phospholipase A2-II in severely traumatized septic patients. Vojnosanit Pregl. 2010;67:893-7.

69. Billeter A, Turina M, Seifert B, Mica L, Stocker R, Keel M. Early serum procalcitonin, interleukin-6, and 24-hour lactate clearance: useful indicators of septic infections in severely traumatized patients. World J Surg. 2009;33:558-66.

70. Latifi SQ, O'Riordan MA, Levine AD, Stallion A. Persistent elevation of serum interleukin-6 in intraabdominal sepsis identifies those with prolonged length of stay. J Pediatr Surg. 2004;39:1548-52.

71. von Lilienfeld-Toal M, Dietrich MP, Glasmacher A, Lehmann L, Breig P, Hahn C et al. Markers of bacteremia in febrile neutropenic patients with hematological malignancies: procalcitonin and IL-6 are more reliable than C-reactive protein. Eur J Clin Microbiol Infect Dis. 2004;23:539-44.

72. Pavcnik-Arnol M, Hojker S, Derganc M. Lipopolysaccharide-binding protein in critically ill neonates and children with suspected infection: comparison with procalcitonin, interleukin-6, and C-reactive protein. Intensive Care Med. 2004;30:1454-60.

73. Dollner H, Vatten L, Austgulen R. Early diagnostic markers for neonatal sepsis: comparing C-reactive protein, interleukin-6, soluble tumour necrosis factor receptors and soluble adhesion molecules. J Clin Epidemiol. 2001;54:1251-7.

74. Tsokos M, Reichelt U, Jung R, Nierhaus A, Puschel K. Interleukin-6 and C-reactive protein serum levels in sepsis-related fatalities during the early postmortem period. Forensic Sci Int. 2001;119:47-56.

75. Lacour AG, Gervaix A, Zamora SA, Vadas L, Lombard PR, Dayer JM et al. Procalcitonin, IL-6, IL-8, IL-1 receptor antagonist and C-reactive protein as identificators of serious bacterial infections in children with fever without localising signs. Eur J Pediatr. 2001;160:95-100.

76. Mandi Y, Farkas G, Takacs T, Boda K, Lonovics J. Diagnostic relevance of procalcitonin, IL-6, and sICAM-1 in the prediction of infected necrosis in acute pancreatitis. Int J Pancreatol. 2000;28:41-9.

77. Magudumana MO, Ballot DE, Cooper PA, Trusler J, Cory BJ, Viljoen E et al. Serial interleukin 6 measurements in the early diagnosis of neonatal sepsis. J Trop Pediatr. 2000;46:267-71.

78. de Bont ES, Vellenga E, Swaanenburg JC, Fidler V, Visser-van Brummen PJ, Kamps WA. Plasma IL-8 and IL-6 levels can be used to define a group with low risk of septicaemia among cancer patients with fever and neutropenia. Br J Haematol. 1999;107:375-80.

79. Diepold M, Noellke P, Duffner U, Kontny U, Berner R. Performance of Interleukin-6 and Interleukin-8 serum levels in pediatric oncology patients with neutropenia and fever for the assessment of low-risk. BMC Infect Dis. 2008;8:28.

80. Patel RT, Deen KI, Youngs D, Warwick J, Keighley MR. Interleukin 6 is a prognostic indicator of outcome in severe intra-abdominal sepsis. Br J Surg. 1994;81:1306-8.

81. Weidhase L, Wellhofer D, Schulze G, Kaiser T, Drogies T, Wurst U et al. Is Interleukin-6 a better predictor of successful antibiotic therapy than procalcitonin and C-reactive protein? A single center study in critically ill adults. BMC Infect Dis. 2019;19:150.

82. Henning DJ, Hall MK, Watsjold BK, Bhatraju PK, Kosamo S, Shapiro NI et al. Interleukin-6 improves infection identification when added to physician judgment during evaluation of potentially septic patients. Am J Emerg Med. 2019;158361.

83. Jawa RS, Anillo S, Huntoon K, Baumann H, Kulaylat M. Analytic review: Interleukin-6 in surgery, trauma, and critical care: part I: basic science. J Intensive Care Med. 2011;26:3-12.

84. Kraft R, Herndon DN, Finnerty CC, Cox RA, Song J, Jeschke MG. Predictive value of IL-8 for sepsis and severe infections after burn injury: A clinical study. Shock. 2015;43:222-7.

85. Calfee CS, Thompson BT, Parsons PE, Ware LB, Matthay MA, Wong HR. Plasma interleukin-8 is not an effective risk stratification tool for adults with vasopressor-dependent septic shock. Crit Care Med. 2010;38:1436-41.

86. Hynninen M, Valtonen M, Vaara M, Markkanen H, Kuusela P, Saxen H et al. Plasma interleukin-8, interleukin-10, and E-selectin levels in neutropenic and non-neutropenic bacteremic patients. Eur J Clin Microbiol Infect Dis. 1997;16:587-91.

87. El-Maghraby SM, Moneer MM, Ismail MM, Shalaby LM, El-Mahallawy HA. The diagnostic value of C-reactive protein, interleukin-8, and monocyte chemotactic protein in risk stratification of febrile neutropenic children with hematologic malignancies. J Pediatr Hematol Oncol. 2007;29:131-6.

88. Fujishima S, Sasaki J, Shinozawa Y, Takuma K, Kimura H, Suzuki M et al. Serum MIP-1 alpha and IL-8 in septic patients. Intensive Care Med. 1996;22:1169-75.

89. Hechtman DH, Cybulsky MI, Fuchs HJ, Baker JB, Gimbrone MA, Jr. Intravascular IL-8. Inhibitor of polymorphonuclear leukocyte accumulation at sites of acute inflammation. J Immunol. 1991;147:883-92.

90. Rose WE, Shukla SK, Berti AD, Hayney MS, Henriquez KM, Ranzoni A et al. Increased endovascular Staphylococcus aureus inoculum Is the link between elevated serum interleukin 10 concentrations and mortality in patients with bacteremia. Clin Infect Dis. 2017;64:1406-12.

91. Matera G, Puccio R, Giancotti A, Quirino A, Pulicari MC, Zicca E et al. Impact of interleukin-10, soluble CD25 and interferon-gamma on the prognosis and early diagnosis of bacteremic systemic inflammatory response syndrome: a prospective observational study. Crit Care. 2013;17:R64.

92. Zeitoun AA, Gad SS, Attia FM, Abu Maziad AS, Bell EF. Evaluation of neutrophilic CD64, interleukin 10 and procalcitonin as diagnostic markers of early- and late-onset neonatal sepsis. Scand J Infect Dis. 2010;42:299-305.

93. Spasova MI, Terzieva DD, Tzvetkova TZ, Stoyanova AA, Mumdzhiev IN, Yanev IB et al. Interleukin-6, interleukin-8, interleukin-10, and C-reactive protein in febrile neutropenia in children with malignant diseases. Folia Med (Plovdiv). 2005;47:46-52.

94. Marchant A, Alegre ML, Hakim A, Pierard G, Marecaux G, Friedman G et al. Clinical and biological significance of interleukin-10 plasma levels in patients with septic shock. J Clin Immunol. 1995;15:266-73.

95. Heper Y, Akalin EH, Mistik R, Akgoz S, Tore O, Goral G et al. Evaluation of serum C-reactive protein, procalcitonin, tumor necrosis factor alpha, and interleukin-10 levels as diagnostic and prognostic parameters in patients with community-acquired sepsis, severe sepsis, and septic shock. Eur J Clin Microbiol Infect Dis. 2006;25:481-91.

96. Wang CH, Gee MJ, Yang C, Su YC. A new model for outcome prediction in intra-abdominal sepsis by the linear discriminant function analysis of IL-6 and IL-10 at different heart rates. J Surg Res. 2006;132:46-51.

97. Potjo M, Theron AJ, Cockeran R, Sipholi NN, Steel HC, Bale TV et al. Interleukin-10 and interleukin-1 receptor antagonist distinguish between patients with sepsis and the systemic inflammatory response syndrome (SIRS). Cytokine. 2019;120:227-33.

98. Ouyang W, Rutz S, Crellin NK, Valdez PA, Hymowitz SG. Regulation and functions of the IL-10 family of cytokines in inflammation and disease. Annu Rev Immunol. 2011;29:71-109.

99. Bro-Jeppesen J, Kjaergaard J, Wanscher M, Nielsen N, Friberg H, Bjerre M et al. Systemic inflammatory response and potential prognostic implications after out-of-hospital cardiac

arrest: A substudy of the Target Temperature Management trial. Crit Care Med. 2015;43:1223-32.

100. Sherwin C, Broadbent R, Young S, Worth J, McCaffrey F, Medlicott NJ et al. Utility of interleukin-12 and interleukin-10 in comparison with other cytokines and acute-phase reactants in the diagnosis of neonatal sepsis. Am J Perinatol. 2008;25:629-36.

101. Novotny AR, Emmanuel K, Ulm K, Bartels H, Siewert JR, Weighardt H et al. Blood interleukin 12 as preoperative predictor of fatal postoperative sepsis after neoadjuvant radiochemotherapy. Br J Surg. 2006;93:1283-9.

102. Castellheim A, Thorgersen EB, Hellerud BC, Pharo A, Johansen HT, Brosstad F et al. New biomarkers in an acute model of live Escherichia coli-induced sepsis in pigs. Scand J Immunol. 2008;68:75-84.

103. Weighardt H, Heidecke CD, Westerholt A, Emmanuilidis K, Maier S, Veit M et al. Impaired monocyte IL-12 production before surgery as a predictive factor for the lethal outcome of postoperative sepsis. Ann Surg. 2002;235:560-7.

104. Steinhauser ML, Hogaboam CM, Lukacs NW, Strieter RM, Kunkel SL. Multiple roles for IL-12 in a model of acute septic peritonitis. J Immunol. 1999;162:5437-43.

105. Collighan N, Giannoudis PV, Kourgeraki O, Perry SL, Guillou PJ, Bellamy MC. Interleukin 13 and inflammatory markers in human sepsis. Br J Surg. 2004;91:762-8.

106. Matsukawa A, Hogaboam CM, Lukacs NW, Lincoln PM, Evanoff HL, Strieter RM et al. Expression and contribution of endogenous IL-13 in an experimental model of sepsis. J Immunol. 2000;164:2738-44.

107. McKenzie AN, Culpepper JA, de Waal MR, Briere F, Punnonen J, Aversa G et al. Interleukin 13, a T-cell-derived cytokine that regulates human monocyte and B-cell function. Proc Natl Acad Sci U S A. 1993;90:3735-9.

108. Kimura A, Ono S, Hiraki S, Takahata R, Tsujimoto H, Miyazaki H et al. The postoperative serum interleukin-15 concentration correlates with organ dysfunction and the prognosis of septic patients following emergency gastrointestinal surgery. J Surg Res. 2012;175:e83-e88.

109. Hiromatsu T, Yajima T, Matsuguchi T, Nishimura H, Wajjwalku W, Arai T et al. Overexpression of interleukin-15 protects against Escherichia coli-induced shock accompanied by inhibition of tumor necrosis factor-alpha-induced apoptosis. J Infect Dis. 2003;187:1442-51.

110. Ahmed Ali M, Mikhael ES, Abdelkader A, Mansour L, El Essawy R, El Sayed R et al. Interleukin-17 as a predictor of sepsis in polytrauma patients: a prospective cohort study. Eur J Trauma Emerg Surg. 2018;44:621-6.

111. Schwarzenberger P, Huang W, Ye P, Oliver P, Manuel M, Zhang Z et al. Requirement of endogenous stem cell factor and granulocyte-colony-stimulating factor for IL-17-mediated granulopoiesis. J Immunol. 2000;164:4783-9.

112. Emmanuilidis K, Weighardt H, Matevossian E, Heidecke CD, Ulm K, Bartels H et al. Differential regulation of systemic IL-18 and IL-12 release during postoperative sepsis: high serum IL-18 as an early predictive indicator of lethal outcome. Shock. 2002;18:301-5.

113. Endo S, Inada K, Yamada Y, Wakabayashi G, Ishikura H, Tanaka T et al. Interleukin 18 (IL-18) levels in patients with sepsis. J Med. 2000;31:15-20.

114. Nakamura T, Kawagoe Y, Suzuki T, Shoji H, Ueda Y, Kobayashi N et al. Changes in plasma interleukin-18 by direct hemoperfusion with polymyxin B-immobilized fiber in patients with septic shock. Blood Purif. 2005;23:417-20.

115. Oberholzer A, Steckholzer U, Kurimoto M, Trentz O, Ertel W. Interleukin-18 plasma levels are increased in patients with sepsis compared to severely injured patients. Shock. 2001;16:411-4.

116. Wawrocki S, Druszczynska M, Kowalewicz-Kulbat M, Rudnicka W. Interleukin 18 (IL-18) as a target for immune intervention. Acta Biochim Pol. 2016;63:59-63.

117. Wong HR, Cvijanovich NZ, Hall M, Allen GL, Thomas NJ, Freishtat RJ et al. Interleukin-27 is a novel candidate diagnostic biomarker for bacterial infection in critically ill children. Crit Care. 2012;16:R213.

118. Wong HR, Lindsell CJ, Lahni P, Hart KW, Gibot S. Interleukin 27 as a sepsis diagnostic biomarker in critically ill adults. Shock. 2013;40:382-6.

119. Wong HR, Liu KD, Kangelaris KN, Lahni P, Calfee CS. Performance of interleukin-27 as a sepsis diagnostic biomarker in critically ill adults. J Crit Care. 2014;29:718-22.

120. Hanna WJ, Berrens Z, Langner T, Lahni P, Wong HR. Interleukin-27: a novel biomarker in predicting bacterial infection among the critically ill. Crit Care. 2015;19:378.

121. Wirtz S, Tubbe I, Galle PR, Schild HJ, Birkenbach M, Blumberg RS et al. Protection from lethal septic peritonitis by neutralizing the biological function of interleukin 27. J Exp Med. 2006;203:1875-81.

122. Wang YC, Weng GP, Liu JP, Li L, Cheng QH. Elevated serum IL-37 concentrations in patients with sepsis. Medicine (Baltimore). 2019;98:e14756.

123. Li S, Neff CP, Barber K, Hong J, Luo Y, Azam T et al. Extracellular forms of IL-37 inhibit innate inflammation in vitro and in vivo but require the IL-1 family decoy receptor IL-1R8. Proc Natl Acad Sci U S A. 2015;112:2497-502.

124. Korn T, Bettelli E, Oukka M, Kuchroo VK. IL-17 and Th17 Cells. Annu Rev Immunol. 2009;27:485-517.

125. Lee K, Huh JW, Lim CM, Koh Y, Hong SB. Clinical role of serum pre-B cell colony-enhancing factor in ventilated patients with sepsis and acute respiratory distress syndrome. Scand J Infect Dis. 2013;45:760-5.

126. Lee KA, Gong MN. Pre-B-cell colony-enhancing factor and its clinical correlates with acute lung injury and sepsis. Chest. 2011;140:382-90.

127. Ye SQ, Simon BA, Maloney JP, Zambelli-Weiner A, Gao L, Grant A et al. Pre-B-cell colony-enhancing factor as a potential novel biomarker in acute lung injury. Am J Respir Crit Care Med. 2005;171:361-70.

128. Karampela I, Christodoulatos GS, Kandri E, Antonakos G, Vogiatzakis E, Dimopoulos G et al. Circulating eNampt and resistin as a proinflammatory duet predicting independently mortality in critically ill patients with sepsis: A prospective observational study. Cytokine. 2019;119:62-70.

129. Sun Z, Lei H, Zhang Z. Pre-B cell colony enhancing factor (PBEF), a cytokine with multiple physiological functions. Cytokine Growth Factor Rev. 2013;24:433-42.

130. Wang Y, Liu Q, Liu T, Zheng Q, Xu X, Liu X et al. Early plasma monocyte chemoattractant protein 1 predicts the development of sepsis in trauma patients: A prospective observational study. Medicine (Baltimore). 2018;97:e0356.

131. Barre M, Behnes M, Hamed S, Pauly D, Lepiorz D, Lang S et al. Revisiting the prognostic value of monocyte chemotactic protein 1 and interleukin-6 in the sepsis-3 era. J Crit Care. 2018;43:21-8.

132. Walborn A, Hoppensteadt D, Syed D, Mosier M, Fareed J. Biomarker profile of sepsis-associated coagulopathy using biochip assay for inflammatory cytokines. Clin Appl Thromb Hemost. 2018;24:625-32.

133. Zhu T, Liao X, Feng T, Wu Q, Zhang J, Cao X et al. Plasma monocyte chemoattractant protein 1 as a predictive marker for sepsis prognosis: A prospective cohort study. Tohoku J Exp Med. 2017;241:139-47.

134. Bozza FA, Salluh JI, Japiassu AM, Soares M, Assis EF, Gomes RN et al. Cytokine profiles as markers of disease severity in sepsis: a multiplex analysis. Crit Care. 2007;11:R49.

135. Jansen PM, van Damme J, Put W, de Jong IW, Taylor FB, Jr., Hack CE. Monocyte chemotactic protein 1 is released during lethal and sublethal bacteremia in baboons. J Infect Dis. 1995;171:1640-2.

136. Tian R, Wang X, Pan T, Li R, Wang J, Liu Z et al. Plasma PTX3, MCP1 and Ang2 are early biomarkers to evaluate the severity of sepsis and septic shock. Scand J Immunol. 2019;e12823.

137. Deshmane SL, Kremlev S, Amini S, Sawaya BE. Monocyte chemoattractant protein-1 (MCP-1): an overview. J Interferon Cytokine Res. 2009;29:313-26.

138. Grimm G, Vila G, Bieglmayer C, Riedl M, Luger A, Clodi M. Changes in osteopontin and in biomarkers of bone turnover during human endotoxemia. Bone. 2010;47:388-91.

139. Roderburg C, Benz F, Cardenas DV, Lutz M, Hippe HJ, Luedde T et al. Persistently elevated osteopontin serum levels predict mortality in critically ill patients. Crit Care. 2015;19:271.

140. Vaschetto R, Nicola S, Olivieri C, Boggio E, Piccolella F, Mesturini R et al. Serum levels of osteopontin are increased in SIRS and sepsis. Intensive Care Med. 2008;34:2176-84.

141. Castello LM, Baldrighi M, Molinari L, Salmi L, Cantaluppi V, Vaschetto R et al. The role of osteopontin as a diagnostic and prognostic biomarker in sepsis and septic shock. Cells. 2019;8:174

142. Scatena M, Liaw L, Giachelli CM. Osteopontin: a multifunctional molecule regulating chronic inflammation and vascular disease. Arterioscler Thromb Vasc Biol. 2007;27:2302-9.

143. Ando K, Kato H, Kotani T, Ozaki M, Arimura Y, Yagi J. Plasma leukocyte cell-derived chemotaxin 2 is associated with the severity of systemic inflammation in patients with sepsis. Microbiol Immunol. 2012;56:708-18.

144. Chen J, Lu XJ, Yang HY, Shi YH. An interaction between a C-type lectin receptor and leukocyte cell-derived chemotaxin 2 of ayu, Plecoglossus altivelis. Fish Shellfish Immunol. 2010;28:245-8.

145. Vermont CL, Hazelzet JA, de Kleijn ED, van den Dobbelsteen GP, de Groot R. CC and CXC chemokine levels in children with meningococcal sepsis accurately predict mortality and disease severity. Crit Care. 2006;10:R33.

146. Rodriguez-Wilhelmi P, Montes R, Matsukawa A, Hurtado V, Montes M, Hermida J et al. Interleukin (IL)-8 and growth related oncogene-alpha in severe endotoxemia and the effects of a tumor necrosis factor-alpha/IL-1beta inhibitor on these chemokines. Exp Mol Pathol. 2002;73:220-9.

147. De Filippo K, Henderson RB, Laschinger M, Hogg N. Neutrophil chemokines KC and macrophage-inflammatory protein-2 are newly synthesized by tissue macrophages using distinct TLR signaling pathways. J Immunol. 2008;180:4308-15.

148. Bacher M, Meinhardt A, Lan HY, Mu W, Metz CN, Chesney JA et al. Migration inhibitory factor expression in experimentally induced endotoxemia. Am J Pathol. 1997;150:235-46.

149. Bozza FA, Gomes RN, Japiassu AM, Soares M, Castro-Faria-Neto HC, Bozza PT et al. Macrophage migration inhibitory factor levels correlate with fatal outcome in sepsis. Shock. 2004;22:309-13.

150. Brenner T, Hofer S, Rosenhagen C, Steppan J, Lichtenstern C, Weitz J et al. Macrophage migration inhibitory factor (MIF) and manganese superoxide dismutase (MnSOD) as early predictors for survival in patients with severe sepsis or septic shock. J Surg Res. 2010;164:e163-e171.

151. Chuang TY, Chang HT, Chung KP, Cheng HS, Liu CY, Liu YC et al. High levels of serum macrophage migration inhibitory factor and interleukin 10 are associated with a rapidly fatal outcome in patients with severe sepsis. Int J Infect Dis. 2014;20:13-7.

152. Grieb G, Simons D, Piatkowski A, Bernhagen J, Steffens G, Pallua N. Macrophage migration inhibitory factor-A potential diagnostic tool in severe burn injuries? Burns. 2010;36:335-42.

153. Kofoed K, Andersen O, Kronborg G, Tvede M, Petersen J, Eugen-Olsen J et al. Use of plasma C-reactive protein, procalcitonin, neutrophils, macrophage migration inhibitory factor, soluble urokinase-type plasminogen activator receptor, and soluble triggering receptor expressed on myeloid cells-1 in combination to diagnose infections: a prospective study. Crit Care. 2007;11:R38.

154. Froidevaux C, Roger T, Martin C, Glauser MP, Calandra T. Macrophage migration inhibitory factor and innate immune responses to bacterial infections. Crit Care Med. 2001;29:S13-S15.

155. Kasztelewicz B, Piotrowska E, Tolloczko J, Borszewska-Kornacka MK, Gregorek H, Dzierzanowska-Fangrat K. Assessment of interleukin-17A, C5a and RANTES for early diagnosis of neonatal sepsis - a preliminary study. Cent Eur J Immunol. 2016;41:376-82.

156. Moller AS, Bjerre A, Brusletto B, Joo GB, Brandtzaeg P, Kierulf P. Chemokine patterns in meningococcal disease. J Infect Dis. 2005;191:768-75.

157. Stojewska M, Wasek-Buko M, Jakub B, Wisniewska-Ulfig D, Goleniowska-Krol A, Szymanska A et al. Evaluation of serum chemokine RANTES concentration as a biomarker in the diagnosis of early-onset severe infections in neonates. Postepy Hig Med Dosw (Online). 2016;70:272-9.

158. VanOtteren GM, Strieter RM, Kunkel SL, Paine R, III, Greenberger MJ, Danforth JM et al. Compartmentalized expression of RANTES in a murine model of endotoxemia. J Immunol. 1995;154:1900-8.

159. Maillard L, Saito N, Hlawaty H, Friand V, Suffee N, Chmilewsky F et al. RANTES/CCL5 mediated-biological effects depend on the syndecan-4/PKCalpha signaling pathway. Biol Open. 2014;3:995-1004.

160. Hayney MS, Henriquez KM, Barnet JH, Ewers T, Champion HM, Flannery S et al. Serum IFN-gamma-induced protein 10 (IP-10) as a biomarker for severity of acute respiratory infection in healthy adults. J Clin Virol. 2017;90:32-7.

161. Ng PC, Li K, Chui KM, Leung TF, Wong RP, Chu WC et al. IP-10 is an early diagnostic marker for identification of late-onset bacterial infection in preterm infants. Pediatr Res. 2007;61:93-8.

162. Dufour JH, Dziejman M, Liu MT, Leung JH, Lane TE, Luster AD. IFN-gamma-inducible protein 10 (IP-10; CXCL10)-deficient mice reveal a role for IP-10 in effector T cell generation and trafficking. J Immunol. 2002;168:3195-204.

163. De Pablo R, Monserrat J, Reyes E, Diaz D, Rodriguez-Zapata M, la Hera Ad et al. Sepsis-induced acute respiratory distress syndrome with fatal outcome is associated to increased serum transforming growth factor beta-1 levels. Eur J Intern Med. 2012;23:358-62.

164. Knapp S, Thalhammer F, Locker GJ, Laczika K, Hollenstein U, Frass M et al. Prognostic value of MIP-1 alpha, TGF-beta 2, sELAM-1, and sVCAM-1 in patients with gram-positive sepsis. Clin Immunol Immunopathol. 1998;87:139-44.

165. Rendon-Ramirez EJ, Ortiz-Stern A, Martinez-Mejia C, Salinas-Carmona MC, Rendon A, Mata-Tijerina VL et al. TGF-beta blood levels distinguish between Influenza A (H1N1)pdm09 virus sepsis and sepsis due to other forms of community-acquired pneumonia. Viral Immunol. 2015;28:248-54.

166. Massague J. The transforming growth factor-beta family. Annu Rev Cell Biol. 1990;6:597-641.

167. Badr HS, El-Gendy FM, Helwa MA. Serum stromal-derived-factor-1 (CXCL12) and its alpha chemokine receptor (CXCR4) as biomarkers in neonatal sepsis. J Matern Fetal Neonatal Med. 2018;31:2209-15.

168. Franchini S, Marciano T, Sorlini C, Campochiaro C, Tresoldi M, Sabbadini MG et al. Serum CXCL12 levels on hospital admission predict mortality in patients with severe sepsis/septic shock. Am J Emerg Med. 2015;33:1802-4.

169. Tunc T, Cekmez F, Cetinkaya M, Kalayci T, Fidanci K, Saldir M et al. Diagnostic value of elevated CXCR4 and CXCL12 in neonatal sepsis. J Matern Fetal Neonatal Med. 2015;28:356-61.

170. Martin C, Burdon PC, Bridger G, Gutierrez-Ramos JC, Williams TJ, Rankin SM. Chemokines acting via CXCR2 and CXCR4 control the release of neutrophils from the bone marrow and their return following senescence. Immunity. 2003;19:583-93.

171. O'Grady NP, Tropea M, Preas HL, Reda D, Vandivier RW, Banks SM et al. Detection of macrophage inflammatory protein (MIP)-1alpha and MIP-1beta during experimental endotoxemia and human sepsis. J Infect Dis. 1999;179:136-41.

172. Tsujimoto H, Ono S, Majima T, Kawarabayashi N, Takayama E, Kinoshita M et al. Neutrophil elastase, MIP-2, and TLR-4 expression during human and experimental sepsis. Shock. 2005;23:39-44.

173. Venet F, Lepape A, Debard AL, Bienvenu J, Bohe J, Monneret G. The Th2 response as monitored by CRTH2 or CCR3 expression is severely decreased during septic shock. Clin Immunol. 2004;113:278-84.

174. Cosmi L, Annunziato F, Maggi E, Romagnani S, Manetti R. Chemoattractant receptors expressed on type 2 T cells and their role in disease. Int Arch Allergy Immunol. 2001;125:273-9.

175. Ding Z, Jia SH, Marshall JC, Downey GP, Waddell TK. Up-regulation of functional CXCR4 expression on human lymphocytes in sepsis. Crit Care Med. 2006;34:3011-7.

176. An JL, Ishida Y, Kimura A, Tsokos M, Kondo T. Immunohistochemical detection of CCR2 and CX3CR1 in sepsis-induced lung injury. Forensic Sci Int. 2009;192:e21-e25.

177. Pachot A, Cazalis MA, Venet F, Turrel F, Faudot C, Voirin N et al. Decreased expression of the fractalkine receptor CX3CR1 on circulating monocytes as new feature of sepsis-induced immunosuppression. J Immunol. 2008;180:6421-9.

178. Rennert K, Heisig K, Groeger M, Wallert M, Funke H, Lorkowski S et al. Recruitment of CD16(+) monocytes to endothelial cells in response to LPS-treatment and concomitant TNF release is regulated by CX3CR1 and interfered by soluble fractalkine. Cytokine. 2016;83:41-52.

179. Gao L, Yang B, Zhang H, Ou Q, Lin Y, Zhang M et al. DcR3, a new biomarker for sepsis, correlates with infection severity and procalcitonin. Oncotarget. 2018;9:10934-44.

180. Hou YQ, Xu P, Zhang M, Han D, Peng L, Liang DY et al. Serum decoy receptor 3, a potential new biomarker for sepsis. Clin Chim Acta. 2012;413:744-8.

181. Kim S, Mi L, Zhang L. Specific elevation of DcR3 in sera of sepsis patients and its potential role as a clinically important biomarker of sepsis. Diagn Microbiol Infect Dis. 2012;73:312-7.

182. Zhao JJ, Lou XL, Chen HW, Zhu FT, Hou YQ. Diagnostic value of decoy receptor 3 combined with procalcitonin and soluble urokinase-type plasminogen activator receptor for sepsis. Cell Mol Biol Lett. 2018;23:22.

183. Kim S, Fotiadu A, Kotoula V. Increased expression of soluble decoy receptor 3 in acutely inflamed intestinal epithelia. Clin Immunol. 2005;115:286-94.

184. Chu CM, Kao KC, Huang SH, Chuang DY, Wu HP. Diagnostic value of apoptosis biomarkers in severe sepsis-A pilot study. Cell Mol Biol (Noisy -le-grand). 2016;62:32-7.

185. De Freitas I, Fernandez-Somoza M, Essenfeld-Sekler E, Cardier JE. Serum levels of the apoptosis-associated molecules, tumor necrosis factor-alpha/tumor necrosis factor type-I receptor and Fas/FasL, in sepsis. Chest. 2004;125:2238-46.

186. Doughty L, Clark RS, Kaplan SS, Sasser H, Carcillo J. sFas and sFas ligand and pediatric sepsis-induced multiple organ failure syndrome. Pediatr Res. 2002;52:922-7.

187. Huttunen R, Syrjanen J, Vuento R, Laine J, Hurme M, Aittoniemi J. Apoptosis markers soluble Fas (sFas), Fas Ligand (FasL) and sFas/FasL ratio in patients with bacteremia: a prospective cohort study. J Infect. 2012;64:276-81.

188. Mikic D, Vasilijic S, CuCuz M, Colic M. Clinical significance of soluble Fas plasma levels in patients with sepsis. Vojnosanit Pregl. 2015;72:608-13.

189. Paunel-Gorgulu A, Flohe S, Scholz M, Windolf J, Logters T. Increased serum soluble Fas after major trauma is associated with delayed neutrophil apoptosis and development of sepsis. Crit Care. 2011;15:R20.

190. Cheng J, Zhou T, Liu C, Shapiro JP, Brauer MJ, Kiefer MC et al. Protection from Fas-mediated apoptosis by a soluble form of the Fas molecule. Science. 1994;263:1759-62.

191. Alves BE, Montalvao SA, Aranha FJ, Lorand-Metze I, De Souza CA, Annichino-Bizzacchi JM et al. Time-course of sFlt-1 and VEGF-A release in neutropenic patients with sepsis and septic shock: a prospective study. J Transl Med. 2011;9:23.

192. Ebihara I, Hirayama K, Kaneko S, Nagai M, Ogawa Y, Fujita S et al. Vascular endothelial growth factor and soluble fms-like tyrosine kinase-1 in septic shock patients treated with direct hemoperfusion with a polymyxin B-immobilized fiber column. Ther Apher Dial. 2008;12:285-91.

193. Shapiro NI, Schuetz P, Yano K, Sorasaki M, Parikh SM, Jones AE et al. The association of endothelial cell signaling, severity of illness, and organ dysfunction in sepsis. Crit Care. 2010;14:R182.

194. Tsao PN, Chan FT, Wei SC, Hsieh WS, Chou HC, Su YN et al. Soluble vascular endothelial growth factor receptor-1 protects mice in sepsis. Crit Care Med. 2007;35:1955-60.

195. Wu FT, Stefanini MO, Mac Gabhann F, Kontos CD, Annex BH, Popel AS. A systems biology perspective on sVEGFR1: its biological function, pathogenic role and therapeutic use. J Cell Mol Med. 2010;14:528-52.

196. Feng L, Zhou X, Su LX, Feng D, Jia YH, Xie LX. Clinical significance of soluble hemoglobin scavenger receptor CD163 (sCD163) in sepsis, a prospective study. PLoS One. 2012;7:e38400.

197. Gaini S, Koldkjaer OG, Pedersen SS, Pedersen C, Moestrup SK, Moller HJ. Soluble haemoglobin scavenger receptor (sCD163) in patients with suspected community-acquired infections. APMIS. 2006;114:103-11.

198. Kjaergaard AG, Rodgaard-Hansen S, Dige A, Krog J, Moller HJ, Tonnesen E. Monocyte expression and soluble levels of the haemoglobin receptor (CD163/sCD163) and the mannose receptor (MR/sMR) in septic and critically ill non-septic ICU patients. PLoS One. 2014;9:e92331.

199. Sapa A, Rak A, Wybieralska M, Machon J, Krzywonos-Zawadzka A, Zawadzki K et al. Diagnostic usefulness of sCD163, procalcitonin and neopterin for sepsis risk assessment in critically ill patients. Adv Clin Exp Med. 2017;26:101-8.

200. Su L, Feng L, Liu C, Jiang Z, Li M, Xiao K et al. Diagnostic value of urine sCD163 levels for sepsis and relevant acute kidney injury: a prospective study. BMC Nephrol. 2012;13:123.

201. Su L, Feng L, Song Q, Kang H, Zhang X, Liang Z et al. Diagnostic value of dynamics serum sCD163, sTREM-1, PCT, and CRP in differentiating sepsis, severity assessment, and prognostic prediction. Mediators Inflamm. 2013;2013:969875.

202. Moller HJ, Moestrup SK, Weis N, Wejse C, Nielsen H, Pedersen SS et al. Macrophage serum markers in pneumococcal bacteremia: Prediction of survival by soluble CD163. Crit Care Med. 2006;34:2561-6.

203. Marie Relster M, Gaini S, Moller HJ, Johansen IS, Pedersen C. The macrophage activation marker sMR as a diagnostic and prognostic marker in patients with acute infectious disease with or without sepsis. Scand J Clin Lab Invest. 2018;78:180-6.

204. Rodgaard-Hansen S, Rafique A, Weis N, Wejse C, Nielsen H, Pedersen SS et al. Increased concentrations of the soluble mannose receptor in serum from patients with pneumococcal bacteraemia, and prediction of survival. Infect Dis (Lond). 2015;47:203-8.

205. Martinez-Pomares L, Mahoney JA, Kaposzta R, Linehan SA, Stahl PD, Gordon S. A functional soluble form of the murine mannose receptor is produced by macrophages in vitro and is present in mouse serum. J Biol Chem. 1998;273:23376-80.

206. Guitton C, Gerard N, Sebille V, Bretonniere C, Zambon O, Villers D et al. Early rise in circulating endothelial protein C receptor correlates with poor outcome in severe sepsis. Intensive Care Med. 2011;37:950-6.

207. Kager LM, Schouten M, Wiersinga WJ, de Boer JD, Lattenist LC, Roelofs JJ et al. Overexpression of the endothelial protein C receptor is detrimental during pneumonia-derived gram-negative sepsis (Melioidosis). PLoS Negl Trop Dis. 2013;7:e2306.

208. Kendirli T, Ince E, Ciftci E, Dogru U, Egin Y, Akar N. Soluble endothelial protein C receptor level in children with sepsis. Pediatr Hematol Oncol. 2009;26:432-8.

209. Vassiliou AG, Kotanidou A, Mastora Z, Maniatis NA, Albani P, Jahaj E et al. Elevated soluble endothelial protein C receptor levels at ICU admission are associated with sepsis development. Minerva Anestesiol. 2015;81:125-34.

210. Liaw PC, Neuenschwander PF, Smirnov MD, Esmon CT. Mechanisms by which soluble endothelial cell protein C receptor modulates protein C and activated protein C function. J Biol Chem. 2000;275:5447-52.

211. Bopp C, Hofer S, Weitz J, Bierhaus A, Nawroth PP, Martin E et al. sRAGE is elevated in septic patients and associated with patients outcome. J Surg Res. 2008;147:79-83.

212. Brodska H, Malickova K, Valenta J, Fabio A, Drabek T. Soluble receptor for advanced glycation end products predicts 28-day mortality in critically ill patients with sepsis. Scand J Clin Lab Invest. 2013;73:650-60.

213. Matsumoto H, Matsumoto N, Ogura H, Shimazaki J, Yamakawa K, Yamamoto K et al. The clinical significance of circulating soluble RAGE in patients with severe sepsis. J Trauma Acute Care Surg. 2015;78:1086-93.

214. Hamasaki MY, Barbeiro HV, de Souza HP, Machado MC, da Silva FP. sRAGE in septic shock: a potential biomarker of mortality. Rev Bras Ter Intensiva. 2014;26:392-6.

215. Hofer S, Uhle F, Fleming T, Hell C, Schmoch T, Bruckner T et al. RAGE-mediated inflammation in patients with septic shock. J Surg Res. 2016;202:315-27.

216. Jabaudon M, Futier E, Roszyk L, Chalus E, Guerin R, Petit A et al. Soluble form of the receptor for advanced glycation end products is a marker of acute lung injury but not of severe sepsis in critically ill patients. Crit Care Med. 2011;39:480-8.

217. Narvaez-Rivera RM, Rendon A, Salinas-Carmona MC, Rosas-Taraco AG. Soluble RAGE as a severity marker in community acquired pneumonia associated sepsis. BMC Infect Dis. 2012;12:15.

218. Yonekura H, Yamamoto Y, Sakurai S, Petrova RG, Abedin MJ, Li H et al. Novel splice variants of the receptor for advanced glycation end-products expressed in human vascular endothelial

cells and pericytes, and their putative roles in diabetes-induced vascular injury. Biochem J. 2003;370:1097-109.

219. Ertel W, Scholl FA, Gallati H, Bonaccio M, Schildberg FW, Trentz O. Increased release of soluble tumor necrosis factor receptors into blood during clinical sepsis. Arch Surg. 1994;129:1330-6.

220. Gordon AC, Lagan AL, Aganna E, Cheung L, Peters CJ, McDermott MF et al. TNF and TNFR polymorphisms in severe sepsis and septic shock: a prospective multicentre study. Genes Immun. 2004;5:631-40.

221. Schroder J, Stuber F, Gallati H, Schade FU, Kremer B. Pattern of soluble TNF receptors I and II in sepsis. Infection. 1995;23:143-8.

222. Van Zee KJ, Kohno T, Fischer E, Rock CS, Moldawer LL, Lowry SF. Tumor necrosis factor soluble receptors circulate during experimental and clinical inflammation and can protect against excessive tumor necrosis factor alpha in vitro and in vivo. Proc Natl Acad Sci U S A. 1992;89:4845-9.

223. Adly AA, Ismail EA, Andrawes NG, El-Saadany MA. Circulating soluble triggering receptor expressed on myeloid cells-1 (sTREM-1) as diagnostic and prognostic marker in neonatal sepsis. Cytokine. 2014;65:184-91.

224. Charles PE, Noel R, Massin F, Guy J, Bollaert PE, Quenot JP et al. Significance of soluble triggering receptor expressed on myeloid cells-1 elevation in patients admitted to the intensive care unit with sepsis. BMC Infect Dis. 2016;16:559.

225. Gibot S, Massin F, Le RP, Bene MC, Faure GC, Bollaert PE et al. Surface and soluble triggering receptor expressed on myeloid cells-1: expression patterns in murine sepsis. Crit Care Med. 2005;33:1787-93.

226. Halim B, Ozlem T, Melek C, Munip HY, Cevval U, Gonul DH et al. Diagnostic and prognostic value of procalcitonin and sTREM-1 levels in sepsis. Turk J Med Sci. 2015;45:578-86.

227. Horst SA, Linner A, Beineke A, Lehne S, Holtje C, Hecht A et al. Prognostic value and therapeutic potential of TREM-1 in Streptococcus pyogenes- induced sepsis. J Innate Immun. 2013;5:581-90.

228. Jeong SJ, Song YG, Kim CO, Kim HW, Ku NS, Han SH et al. Measurement of plasma sTREM-1 in patients with severe sepsis receiving early goal-directed therapy and evaluation of its usefulness. Shock. 2012;37:574-8.

229. Li Z, Wang H, Liu J, Chen B, Li G. Serum soluble triggering receptor expressed on myeloid cells-1 and procalcitonin can reflect sepsis severity and predict prognosis: a prospective cohort study. Mediators Inflamm. 2014;2014:641039.

230. Li Z, Zhang E, Hu Y, Liu Y, Chen B. High serum sTREM-1 correlates with myocardial dysfunction and predicts prognosis in septic patients. Am J Med Sci. 2016;351:555-62.

231. Ravetti CG, Moura AD, Vieira EL, Pedroso ER, Teixeira AL. sTREM-1 predicts intensive care unit and 28-day mortality in cancer patients with severe sepsis and septic shock. J Crit Care. 2015;30:440-13.

232. Su L, Han B, Liu C, Liang L, Jiang Z, Deng J et al. Value of soluble TREM-1, procalcitonin, and Creactive protein serum levels as biomarkers for detecting bacteremia among sepsis patients

with new fever in intensive care units: a prospective cohort study. BMC Infect Dis. 2012;12:157.

233. Jedynak M, Siemiatkowski A, Milewski R, Mroczko B, Szmitkowski M. Diagnostic effectiveness of soluble triggering receptor expressed on myeloid cells-1 in sepsis, severe sepsis and septic shock. Arch Med Sci. 2019;15:713-21.

234. Gibot S, Cravoisy A, Kolopp-Sarda MN, Bene MC, Faure G, Bollaert PE et al. Time-course of sTREM (soluble triggering receptor expressed on myeloid cells)-1, procalcitonin, and C-reactive protein plasma concentrations during sepsis. Crit Care Med. 2005;33:792-6.

235. Molkanen T, Ruotsalainen E, Thorball CW, Jarvinen A. Elevated soluble urokinase plasminogen activator receptor (suPAR) predicts mortality in Staphylococcus aureus bacteremia. Eur J Clin Microbiol Infect Dis. 2011;30:1417-24.

236. Casagranda I, Vendramin C, Callegari T, Vidali M, Calabresi A, Ferrandu G et al. Usefulness of suPAR in the risk stratification of patients with sepsis admitted to the emergency department. Intern Emerg Med. 2015;10:725-30.

237. Donadello K, Scolletta S, Taccone FS, Covajes C, Santonocito C, Cortes DO et al. Soluble urokinase-type plasminogen activator receptor as a prognostic biomarker in critically ill patients. J Crit Care. 2014;29:144-9.

238. El-Mekkawy MS, Saleh NY, Sonbol AA. Soluble urokinase plasminogen activator receptor: A new biomarker in the pediatric intensive care unit. Indian J Pediatr. 2016;83:661-9.

239. Giamarellos-Bourboulis EJ, Norrby-Teglund A, Mylona V, Savva A, Tsangaris I, Dimopoulou I et al. Risk assessment in sepsis: a new prognostication rule by APACHE II score and serum soluble urokinase plasminogen activator receptor. Crit Care. 2012;16:R149.

240. Hoenigl M, Raggam RB, Wagner J, Valentin T, Leitner E, Seeber K et al. Diagnostic accuracy of soluble urokinase plasminogen activator receptor (suPAR) for prediction of bacteremia in patients with systemic inflammatory response syndrome. Clin Biochem. 2013;46:225-9.

241. Huttunen R, Syrjanen J, Vuento R, Hurme M, Huhtala H, Laine J et al. Plasma level of soluble urokinase-type plasminogen activator receptor as a predictor of disease severity and case fatality in patients with bacteraemia: a prospective cohort study. J Intern Med. 2011;270:32-40.

242. Khater WS, Salah-Eldeen NN, Khater MS, Saleh AN. Role of suPAR and lactic acid in diagnosing sepsis and predicting mortality in elderly patients. Eur J Microbiol Immunol (Bp). 2016;6:178-85.

243. Liu X, Shen Y, Li Z, Fei A, Wang H, Ge Q et al. Prognostic significance of APACHE II score and plasma suPAR in Chinese patients with sepsis: a prospective observational study. BMC Anesthesiol. 2016;16:46.

244. Okulu E, Arsan S, Akin IM, Ates C, Alan S, Kilic A et al. Serum levels of soluble urokinase plasminogen activator receptor in infants with late-onset sepsis. J Clin Lab Anal. 2015;29:347-52.

245. Savva A, Raftogiannis M, Baziaka F, Routsi C, Antonopoulou A, Koutoukas P et al. Soluble urokinase plasminogen activator receptor (suPAR) for assessment of disease severity in ventilator-associated pneumonia and sepsis. J Infect. 2011;63:344-50.

246. Siahanidou T, Margeli A, Tsirogianni C, Charoni S, Giannaki M, Vavourakis E et al. Clinical value of plasma soluble urokinase-type plasminogen activator receptor levels in term neonates with infection or sepsis: a prospective study. Mediators Inflamm. 2014;2014:375702.

247. Suberviola B, Castellanos-Ortega A, Ruiz Ruiz A, Lopez-Hoyos M, Santibanez M. Hospital mortality prognostication in sepsis using the new biomarkers suPAR and proADM in a single determination on ICU admission. Intensive Care Med. 2013;39:1945-52.

248. Sunnetcioglu A, Sunnetcioglu M, Adiyaman F, Binici I, Soyoral L. Could soluble urokinase plasminogen receptor (suPAR) be used as a diagnostic biomarker for ventilator-associated pneumonia? Clin Respir J. 2017;11:925-30.

249. Uusitalo-Seppala R, Huttunen R, Tarkka M, Aittoniemi J, Koskinen P, Leino A et al. Soluble urokinase-type plasminogen activator receptor in patients with suspected infection in the emergency room: a prospective cohort study. J Intern Med. 2012;272:247-56.

250. Wittenhagen P, Kronborg G, Weis N, Nielsen H, Obel N, Pedersen SS et al. The plasma level of soluble urokinase receptor is elevated in patients with Streptococcus pneumoniae bacteraemia and predicts mortality. Clin Microbiol Infect. 2004;10:409-15.

251. Zeng M, Chang M, Zheng H, Li B, Chen Y, He W et al. Clinical value of soluble urokinase-type plasminogen activator receptor in the diagnosis, prognosis, and therapeutic guidance of sepsis. Am J Emerg Med. 2016;34:375-80.

252. van Oort PM, Bos LD, Povoa P, Ramirez P, Torres A, Artigas A et al. Soluble urokinase plasminogen activator receptor for the prediction of ventilator-associated pneumonia. ERJ Open Res. 2019;5:00212-2018

253. Nusshag C, Rupp C, Schmitt F, Krautkramer E, Speer C, Kalble F et al. Cell cycle biomarkers and soluble urokinase-type plasminogen activator receptor for the prediction of sepsis-induced acute kidney injury requiring renal replacement therapy: A prospective, exploratory study. Crit Care Med. 2019;47:e999–e1007

254. Backes Y, van der Sluijs KF, Mackie DP, Tacke F, Koch A, Tenhunen JJ et al. Usefulness of suPAR as a biological marker in patients with systemic inflammation or infection: a systematic review. Intensive Care Med. 2012;38:1418-28.

255. Drouin SM, Kildsgaard J, Haviland J, Zabner J, Jia HP, McCray PB, Jr. et al. Expression of the complement anaphylatoxin C3a and C5a receptors on bronchial epithelial and smooth muscle cells in models of sepsis and asthma. J Immunol. 2001;166:2025-32.

256. Delogu G, Casula MA, Mancini P, Tellan G, Signore L. Serum neopterin and soluble interleukin-2 receptor for prediction of a shock state in gram-negative sepsis. J Crit Care. 1995;10:64-71.

257. Nelson BH, Willerford DM. Biology of the interleukin-2 receptor. Adv Immunol. 1998;70:1-81.

258. Cekmez F, Fidanci MK, Ayar G, Saldir M, Karaoglu A, Gunduz RC et al. Diagnostic value of Upar, IL-33, and ST2 levels in childhood sepsis. Clin Lab. 2016;62:751-5.

259. Brunner M, Krenn C, Roth G, Moser B, Dworschak M, Jensen-Jarolim E et al. Increased levels of soluble ST2 protein and IgG1 production in patients with sepsis and trauma. Intensive Care Med. 2004;30:1468-73.

260. Hur M, Kim H, Kim HJ, Yang HS, Magrini L, Marino R et al. Soluble ST2 has a prognostic role in patients with suspected sepsis. Ann Lab Med. 2015;35:570-7.

261. Kim H, Hur M, Moon HW, Yun YM, Di Somma S. Multi-marker approach using procalcitonin, presepsin, galectin-3, and soluble suppression of tumorigenicity 2 for the prediction of mortality in sepsis. Ann Intensive Care. 2017;7:27.

262. Yang HS, Hur M, Kim H, Magrini L, Marino R, Di Somma S. Soluble suppression of tumorigenicity 2 and echocardiography in sepsis. Ann Lab Med. 2016;36:590-4.

263. Schmitz J, Owyang A, Oldham E, Song Y, Murphy E, McClanahan TK et al. IL-33, an interleukin-1-like cytokine that signals via the IL-1 receptor-related protein ST2 and induces T helper type 2-associated cytokines. Immunity. 2005;23:479-90.

264. Brunialti MK, Martins PS, Barbosa de Carvalho H, Machado FR, Barbosa LM, Salomao R. TLR2, TLR4, CD14, CD11B, and CD11C expressions on monocytes surface and cytokine production in patients with sepsis, severe sepsis, and septic shock. Shock. 2006;25:351-7.

265. Tansho-Nagakawa S, Ubagai T, Kikuchi-Ueda T, Koshio O, Koshibu Y, Kikuchi H et al. Analysis of membrane antigens on neutrophils from patients with sepsis. J Infect Chemother. 2012;18:646-51.

266. Williams DL, Ha T, Li C, Kalbfleisch JH, Schweitzer J, Vogt W et al. Modulation of tissue Toll-like receptor 2 and 4 during the early phases of polymicrobial sepsis correlates with mortality. Crit Care Med. 2003;31:1808-18.

267. Salomao R, Martins PS, Brunialti MK, Fernandes ML, Martos LS, Mendes ME et al. TLR signaling pathway in patients with sepsis. Shock. 2008;30 Suppl 1:73-7.

268. Guo RF, Riedemann NC, Bernacki KD, Sarma VJ, Laudes IJ, Reuben JS et al. Neutrophil C5a receptor and the outcome in a rat model of sepsis. FASEB J. 2003;17:1889-91.

269. Huber-Lang M, Sarma JV, Rittirsch D, Schreiber H, Weiss M, Flierl M et al. Changes in the novel orphan, C5a receptor (C5L2), during experimental sepsis and sepsis in humans. J Immunol. 2005;174:1104-10.

270. Unnewehr H, Rittirsch D, Sarma JV, Zetoune F, Flierl MA, Perl M et al. Changes and regulation of the C5a receptor on neutrophils during septic shock in humans. J Immunol. 2013;190:4215-25.

271. Xu R, Lin F, Bao C, Huang H, Ji C, Wang S et al. Complement 5a receptor-mediated neutrophil dysfunction is associated with a poor outcome in sepsis. Cell Mol Immunol. 2016;13:103-9.

272. Guo RF, Riedemann NC, Ward PA. Role of C5a-C5aR interaction in sepsis. Shock. 2004;21:1-7.

273. Kjaergaard AG, Nielsen JS, Tonnesen E, Krog J. Expression of NK cell and monocyte receptors in critically ill patients--potential biomarkers of sepsis. Scand J Immunol. 2015;81:249-58.

274. Marras F, Bozzano F, De Maria A. Involvement of activating NK cell receptors and their modulation in pathogen immunity. J Biomed Biotechnol. 2011;2011:152430.

275. Nolan A, Weiden M, Kelly A, Hoshino Y, Hoshino S, Mehta N et al. CD40 and CD80/86 act synergistically to regulate inflammation and mortality in polymicrobial sepsis. Am J Respir Crit Care Med. 2008;177:301-8.

276. Arad G, Levy R, Nasie I, Hillman D, Rotfogel Z, Barash U et al. Binding of superantigen toxins into the CD28 homodimer interface is essential for induction of cytokine genes that mediate lethal shock. PLoS Biol. 2011;9:e1001149.

277. de Jong E, de Lange DW, Beishuizen A, van de Ven PM, Girbes AR, Huisman A. Neutrophil CD64 expression as a longitudinal biomarker for severe disease and acute infection in critically ill patients. Int J Lab Hematol. 2016;38:576-84.

278. Du J, Li L, Dou Y, Li P, Chen R, Liu H. Diagnostic utility of neutrophil CD64 as a marker for early-onset sepsis in preterm neonates. PLoS One. 2014;9:e102647.

279. El Shimi MS, Abou Shady NM, Hamed GM, Shedeed NS. Significance of neutrophilic CD64 as an early marker for detection of neonatal sepsis and prediction of disease outcome. J Matern Fetal Neonatal Med. 2017;30:1709-14.

280. Elawady S, Botros SK, Sorour AE, Ghany EA, Elbatran G, Ali R. Neutrophil CD64 as a diagnostic marker of sepsis in neonates. J Investig Med. 2014;62:644-9.

281. Fang DH, Fan CH, Li J, An Q, Yao H, Ji Q et al. Ratios of CD64 expressed on neutrophils, monocytes, and lymphocytes may be a novel method for diagnosis of neonatal sepsis. J Infect Dev Ctries. 2015;9:175-81.

282. Farias MG, de Lucena NP, Dal BS, de Castro SM. Neutrophil CD64 expression as an important diagnostic marker of infection and sepsis in hospital patients. J Immunol Methods. 2014;414:65-8.

283. Jamsa J, Ala-Kokko T, Huotari V, Ohtonen P, Savolainen ER, Syrjala H. Neutrophil CD64, C-reactive protein, and procalcitonin in the identification of sepsis in the. J Crit Care. 2018;43:139-42.

284. Jamsa J, Huotari V, Savolainen ER, Syrjala H, Ala-Kokko T. Kinetics of leukocyte CD11b and CD64 expression in severe sepsis and non-infectious critical care patients. Acta Anaesthesiol Scand. 2015;59:881-91.

285. Jukic T, Ihan A, Stubljar D. Dynamics of inflammation biomarkers C-reactive protein, leukocytes, neutrophils, and CD64 on neutrophils before and after major surgical procedures to recognize potential postoperative infection. Scand J Clin Lab Invest. 2015;75:500-7.

286. Kipfmueller F, Schneider J, Prusseit J, Dimitriou I, Zur B, Franz AR et al. Role of neutrophil CD64 index as a screening marker for late-onset sepsis in very low birth weight infants. PLoS One. 2015;10:e0124634.

287. Livaditi O, Kotanidou A, Psarra A, Dimopoulou I, Sotiropoulou C, Augustatou K et al. Neutrophil CD64 expression and serum IL-8: sensitive early markers of severity and outcome in sepsis. Cytokine. 2006;36:283-90.

288. Lynema S, Marmer D, Hall ES, Meinzen-Derr J, Kingma PS. Neutrophil CD64 as a diagnostic marker of sepsis: impact on neonatal care. Am J Perinatol. 2015;32:331-6.

289. Motta M, Zini A, Regazzoli A, Garzoli E, Chirico G, Caimi L et al. Diagnostic accuracy and prognostic value of the CD64 index in very low birth weight neonates as a marker of early-onset sepsis. Scand J Infect Dis. 2014;46:433-9.

290. Muzlovic I, Ihan A, Stubljar D. CD64 index on neutrophils can diagnose sepsis and predict 30-day survival in subjects after ventilator-associated pneumonia. J Infect Dev Ctries. 2016;10:260-8.

291. Papadimitriou-Olivgeris M, Lekka K, Zisimopoulos K, Spiliopoulou I, Logothetis D, Theodorou G et al. Role of CD64 expression on neutrophils in the diagnosis of sepsis and the prediction of mortality in adult critically ill patients. Diagn Microbiol Infect Dis. 2015;82:234-9.

292. Pradhan R, Jain P, Paria A, Saha A, Sahoo J, Sen A et al. Ratio of neutrophilic CD64 and monocytic HLA-DR: A novel parameter in diagnosis and prognostication of neonatal sepsis. Cytometry B Clin Cytom. 2016;90:295-302.

293. Djebara S, Biston P, Fosse E, Daper A, Joris M, Boudjeltia KZ et al. Time course of CD64, a leukocyte activation marker, during cardiopulmonary bypass surgery. Shock. 2017;47:158-64.

294. Sheneef A, Mohamed T, Boraey NF, Mohammed MA. Neutrophil CD11b, CD64 and lipocalin-2: Early diagnostic markers of neonatal sepsis. Egypt J Immunol. 2017;24:29-36.

295. Stubljar D, Skvarc M. Expression of CD64 on neutrophils can be used to predict the severity of bloodstream infection before broad range 16S rRNA PCR. Folia Microbiol (Praha). 2015;60:111-8.

296. Tan TL, Ahmad NS, Nasuruddin DN, Ithnin A, Tajul AK, Zaini IZ et al. CD64 and Group II secretory phospholipase A2 (sPLA2-IIA) as biomarkers for distinguishing adult sepsis and bacterial infections in the emergency department. PLoS One. 2016;11:e0152065.

297. Yang AP, Liu J, Yue LH, Wang HQ, Yang WJ, Yang GH. Neutrophil CD64 combined with PCT, CRP and WBC improves the sensitivity for the early diagnosis of neonatal sepsis. Clin Chem Lab Med. 2016;54:345-51.

298. Dimoula A, Pradier O, Kassengera Z, Dalcomune D, Turkan H, Vincent JL. Serial determinations of neutrophil CD64 expression for the diagnosis and monitoring of sepsis in critically ill patients. Clin Infect Dis. 2014;58:820-9.

299. Ajmani S, Singh H, Chaturvedi S, Mishra R, Rai MK, Jain A et al. Utility of neutrophil CD64 and serum TREM-1 in distinguishing bacterial infection from disease flare in SLE and ANCA-associated vasculitis. Clin Rheumatol. 2019;38:997-1005.

300. Barth E, Fischer G, Schneider EM, Wollmeyer J, Georgieff M, Weiss M. Differences in the expression of CD64 and mCD14 on polymorphonuclear cells and on monocytes in patients with septic shock. Cytokine. 2001;14:299-302.

301. Demaret J, Villars-Mechin A, Lepape A, Plassais J, Vallin H, Malcus C et al. Elevated plasmatic level of soluble IL-7 receptor is associated with increased mortality in septic shock patients. Intensive Care Med. 2014;40:1089-96.

302. Lundstrom W, Highfill S, Walsh ST, Beq S, Morse E, Kockum I et al. Soluble IL7Ralpha potentiates IL-7 bioactivity and promotes autoimmunity. Proc Natl Acad Sci U S A. 2013;110:E1761-E1770.

303. Ali FT, Ali MA, Elnakeeb MM, Bendary HN. Presepsin is an early monitoring biomarker for predicting clinical outcome in patients with sepsis. Clin Chim Acta. 2016;460:93-101.

304. Amer HA, Ghareeb H, Lotfy NM, El-Azizi NO, Mahmoud AM. Presepsin a diagnostic marker for sepsis in intensive care unit patients. Egypt J Immunol. 2016;23:109-18.

305. Brodska H, Valenta J, Pelinkova K, Stach Z, Sachl R, Balik M et al. Diagnostic and prognostic value of presepsin vs. established biomarkers in critically ill patients with sepsis or systemic inflammatory response syndrome. Clin Chem Lab Med. 2018;56:658-68.

306. Carpio R, Zapata J, Spanuth E, Hess G. Utility of presepsin (sCD14-ST) as a diagnostic and prognostic marker of sepsis in the emergency department. Clin Chim Acta. 2015;450:169-75.

307. Endo S, Suzuki Y, Takahashi G, Shozushima T, Ishikura H, Murai A et al. Usefulness of presepsin in the diagnosis of sepsis in a multicenter prospective study. J Infect Chemother. 2012;18:891-7.

308. Enguix-Armada A, Escobar-Conesa R, Garcia-De La Torre A, De La Torre-Prados MV. Usefulness of several biomarkers in the management of septic patients: C-reactive protein, procalcitonin, presepsin and mid-regional pro-adrenomedullin. Clin Chem Lab Med. 2016;54:163-8.

309. Godnic M, Stubljar D, Skvarc M, Jukic T. Diagnostic and prognostic value of sCD14-ST--presepsin for patients admitted to hospital intensive care unit (ICU). Wien Klin Wochenschr. 2015;127:521-7.

310. Hoshino K, Irie Y, Mizunuma M, Kawano K, Kitamura T, Ishikura H. Incidence of elevated procalcitonin and presepsin levels after severe trauma: a pilot cohort study. Anaesth Intensive Care. 2017;45:600-4.

311. Iskandar A, Arthamin MZ, Indriana K, Anshory M, Hur M, Di Somma S. Comparison between presepsin and procalcitonin in early diagnosis of neonatal sepsis. J Matern Fetal Neonatal Med. 2019;32:3903-8.

312. Miyosawa Y, Akazawa Y, Kamiya M, Nakamura C, Takeuchi Y, Kusakari M et al. Presepsin as a predictor of positive blood culture in suspected neonatal sepsis. Pediatr Int. 2018;60:157-61.

313. Klouche K, Cristol JP, Devin J, Gilles V, Kuster N, Larcher R et al. Diagnostic and prognostic value of soluble CD14 subtype (Presepsin) for sepsis and community-acquired pneumonia in ICU patients. Ann Intensive Care. 2016;6:59.

314. Koakutsu T, Sato T, Aizawa T, Itoi E, Kushimoto S. Postoperative changes in presepsin level and values predictive of surgical site infection after spinal surgery: A single-center, prospective observational study. Spine (Phila Pa 1976). 2018;43:578-84.

315. Koizumi Y, Shimizu K, Shigeta M, Okuno T, Minamiguchi H, Kito K et al. Plasma presepsin level is an early diagnostic marker of severe febrile neutropenia in hematologic malignancy patients. BMC Infect Dis. 2017;17:27.

316. Leli C, Ferranti M, Marrano U, Al Dhahab ZS, Bozza S, Cenci E et al. Diagnostic accuracy of presepsin (sCD14-ST) and procalcitonin for prediction of bacteraemia and bacterial DNAaemia in patients with suspected sepsis. J Med Microbiol. 2016;65:713-9.

317. Masson S, Caironi P, Fanizza C, Thomae R, Bernasconi R, Noto A et al. Circulating presepsin (soluble CD14 subtype) as a marker of host response in patients with severe sepsis or septic shock: data from the multicenter, randomized ALBIOS trial. Intensive Care Med. 2015;41:12-20.

318. Montaldo P, Rosso R, Santantonio A, Chello G, Giliberti P. Presepsin for the detection of early-onset sepsis in preterm newborns. Pediatr Res. 2017;81:329-34.

319. Ozdemir AA, Elgormus Y. Diagnostic value of presepsin in detection of early-onset neonatal sepsis. Am J Perinatol. 2017;34:550-6.

320. Song X, Song Y, Yuan Y, Zhang P, Zhang X. Prognostic value of presepsin for outcomes and complications in enterocutaneous fistula complicated by abdominal sepsis. Int J Surg. 2016;33 Pt A:96-101.

321. Takahashi G, Shibata S, Fukui Y, Okamura Y, Inoue Y. Diagnostic accuracy of procalcitonin and presepsin for infectious disease in patients with acute kidney injury. Diagn Microbiol Infect Dis. 2016;86:205-10.

322. Topcuoglu S, Arslanbuga C, Gursoy T, Aktas A, Karatekin G, Uluhan R et al. Role of presepsin in the diagnosis of late-onset neonatal sepsis in preterm infants. J Matern Fetal Neonatal Med. 2016;29:1834-9.

323. Yu H, Qi Z, Hang C, Fang Y, Shao R, Li C. Evaluating the value of dynamic procalcitonin and presepsin measurements for patients with severe sepsis. Am J Emerg Med. 2017;35:835-41.

324. Liu B, Chen YX, Yin Q, Zhao YZ, Li CS. Diagnostic value and prognostic evaluation of Presepsin for sepsis in an emergency department. Crit Care. 2013;17:R244.

325. Venugopalan DP, Pillai G, Krishnan S. Diagnostic value and prognostic use of presepsin versus procalcitonin in sepsis. Cureus. 2019;11:e5151.

326. Hassan EA, Abdel Rehim AS, Ahmed AO, Abdullahtif H, Attia A. Clinical value of presepsin in comparison to hsCRP as a monitoring and early prognostic marker for sepsis in critically ill patients. Medicina (Kaunas). 2019;55:36.

327. Wright SD, Ramos RA, Tobias PS, Ulevitch RJ, Mathison JC. CD14, a receptor for complexes of lipopolysaccharide (LPS) and LPS binding protein. Science. 1990;249:1431-3.

328. Jalava-Karvinen P, Hohenthal U, Laitinen I, Kotilainen P, Rajamaki A, Nikoskelainen J et al. Simultaneous quantitative analysis of Fc gamma RI (CD64) and CR1 (CD35) on neutrophils in distinguishing between bacterial infections, viral infections, and inflammatory diseases. Clin Immunol. 2009;133:314-23.

329. Mokuda S, Doi O, Takasugi K. Simultaneous quantitative analysis of the expression of CD64 and CD35 on neutrophils as markers to differentiate between bacterial and viral infections in patients with rheumatoid arthritis. Mod Rheumatol. 2012;22:750-7.

330. Nuutila J, Hohenthal U, Laitinen I, Kotilainen P, Rajamaki A, Nikoskelainen J et al. Quantitative analysis of complement receptors, CR1 (CD35) and CR3 (CD11b), on neutrophils improves distinction between bacterial and viral infections in febrile patients: comparison with standard clinical laboratory data. J Immunol Methods. 2006;315:191-201.

331. Roozendaal R, Carroll MC. Complement receptors CD21 and CD35 in humoral immunity. Immunol Rev. 2007;219:157-66.

332. Lang Y, Jiang Y, Gao M, Wang W, Wang N, Wang K et al. Interleukin-1 receptor 2: A new biomarker for sepsis diagnosis and Gram-negative/Gram-positive bacterial differentiation. Shock. 2017;47:119-24.

333. Peters VA, Joesting JJ, Freund GG. IL-1 receptor 2 (IL-1R2) and its role in immune regulation. Brain Behav Immun. 2013;32:1-8.

334. Liu Q, An L, Qi Z, Zhao Y, Li C. Increased expression of programmed cell death-1 in regulatory T cells of patients with severe sepsis and septic shock: An observational clinical study. Scand J Immunol. 2017;86:408-17.

335. Liang SC, Latchman YE, Buhlmann JE, Tomczak MF, Horwitz BH, Freeman GJ et al. Regulation of PD-1, PD-L1, and PD-L2 expression during normal and autoimmune responses. Eur J Immunol. 2003;33:2706-16.

336. Sugimoto K, Galle C, Preiser JC, Creteur J, Vincent JL, Pradier O. Monocyte CD40 expression in severe sepsis. Shock. 2003;19:24-7.

337. Santilli F, Basili S, Ferroni P, Davi G. CD40/CD40L system and vascular disease. Intern Emerg Med. 2007;2:256-68.

338. Spriggs MK, Fanslow WC, Armitage RJ, Belmont J. The biology of the human ligand for CD40. J Clin Immunol. 1993;13:373-80.

339. Giebeler A, Brandenburg LO, Kaldenbach M, Erschfeld S, Wasmuth H, Wruck C et al. Lack of hepatic c-Met and gp130 expression is associated with an impaired antibacterial response and higher lethality after bile duct ligation. Lab Invest. 2012;92:1726-37.

340. Marsik C, Halama T, Cardona F, Schlifke I, Mittermayer F, Jilma B. Endotoxemia enhances expression of the signaling receptor (GP130) on protein and molecular level. Clin Immunol. 2005;114:293-8.

341. Orliac ML, Peroni RN, Abramoff T, Neuman I, Podesta EJ, Adler-Graschinsky E. Increases in vanilloid TRPV1 receptor protein and CGRP content during endotoxemia in rats. Eur J Pharmacol. 2007;566:145-52.

342. Bodkin JV, Fernandes ES. TRPV1 and SP: key elements for sepsis outcome? Br J Pharmacol. 2013;170:1279-92.

343. Guerin E, Orabona M, Raquil MA, Giraudeau B, Bellier R, Gibot S et al. Circulating immature granulocytes with T-cell killing functions predict sepsis deterioration. Crit Care Med. 2014;42:2007-18.

344. Kaneko T, Stearns-Kurosawa DJ, Taylor F, Jr., Twigg M, Osaki K, Kinasewitz GT et al. Reduced neutrophil CD10 expression in nonhuman primates and humans after in vivo challenge with E. coli or lipopolysaccharide. Shock. 2003;20:130-7.

345. Martens A, Eppink GJ, Woittiez AJ, Eidhof H, de Leij LF. Neutrophil function capacity to express CD10 is decreased in patients with septic shock. Crit Care Med. 1999;27:549-53.

346. Adib M, Ostadi V, Navaei F, Saheb FF, Oreizi F, Shokouhi R et al. Evaluation of CD11b expression on peripheral blood neutrophils for early detection of neonatal sepsis. Iran J Allergy Asthma Immunol. 2007;6:93-6.

347. Genel F, Atlihan F, Gulez N, Kazanci E, Vergin C, Terek DT et al. Evaluation of adhesion molecules CD64, CD11b and CD62L in neutrophils and monocytes of peripheral blood for early diagnosis of neonatal infection. World J Pediatr. 2012;8:72-5.

348. Nupponen I, Andersson S, Jarvenpaa AL, Kautiainen H, Repo H. Neutrophil CD11b expression and circulating interleukin-8 as diagnostic markers for early-onset neonatal sepsis. Pediatrics. 2001;108:E12.

349. Nuutila J, Jalava-Karvinen P, Hohenthal U, Laitinen I, Kotilainen P, Rajamaki A et al. CRP/CD11b ratio: a novel parameter for detecting gram-positive sepsis. Hum Immunol. 2009;70:237-43.

350. Russwurm S, Vickers J, Meier-Hellmann A, Spangenberg P, Bredle D, Reinhart K et al. Platelet and leukocyte activation correlate with the severity of septic organ dysfunction. Shock. 2002;17:263-8.

351. Weiss DJ, Welle M, Mortiz A, Walcheck B. Evaluation of leukocyte cell surface markers in dogs with septic and nonseptic inflammatory diseases. Am J Vet Res. 2004;65:59-63.

352. McMillan SJ, Sharma RS, McKenzie EJ, Richards HE, Zhang J, Prescott A et al. Siglec-E is a negative regulator of acute pulmonary neutrophil inflammation and suppresses CD11b beta2-integrin-dependent signaling. Blood. 2013;121:2084-94.

353. Williams MA, White SA, Miller JJ, Toner C, Withington S, Newland AC et al. Granulocyte-macrophage colony-stimulating factor induces activation and restores respiratory burst activity in monocytes from septic patients. J Infect Dis. 1998;177:107-15.

354. Lewis SM, Treacher DF, Edgeworth J, Mahalingam G, Brown CS, Mare TA et al. Expression of CD11c and EMR2 on neutrophils: potential diagnostic biomarkers for sepsis and systemic inflammation. Clin Exp Immunol. 2015;182:184-94.

355. Stacker SA, Springer TA. Leukocyte integrin P150,95 (CD11c/CD18) functions as an adhesion molecule binding to a counter-receptor on stimulated endothelium. J Immunol. 1991;146:648-55.

356. Muller Kobold AC, Zijlstra JG, Koene HR, de Haas M, Kallenberg CG, Tervaert JW. Levels of soluble Fc gammaRIII correlate with disease severity in sepsis. Clin Exp Immunol. 1998;114:220-7.

357. Shalova IN, Kajiji T, Lim JY, Gomez-Pina V, Fernandez-Ruiz I, Arnalich F et al. CD16 regulates TRIF-dependent TLR4 response in human monocytes and their subsets. J Immunol. 2012;188:3584-93.

358. Ebdrup L, Krog J, Granfeldt A, Tonnesen E, Hokland M. Dynamic expression of the signal regulatory protein alpha and CD18 on porcine PBMC during acute endotoxaemia. Scand J Immunol. 2008;68:430-7.

359. Kragstrup TW, Juul-Madsen K, Christiansen SH, Zhang X, Krog J, Vorup-Jensen T et al. Altered levels of soluble CD18 may associate immune mechanisms with outcome in sepsis. Clin Exp Immunol. 2017;190:258-67.

360. Jiang YN, Cai X, Zhou HM, Jin WD, Zhang M, Zhang Y et al. Diagnostic and prognostic roles of soluble CD22 in patients with Gram-negative bacterial sepsis. Hepatobiliary Pancreat Dis Int. 2015;14:523-9.

361. Cho E, Lee JH, Lim HJ, Oh SW, Jo SK, Cho WY et al. Soluble CD25 is increased in patients with sepsis-induced acute kidney injury. Nephrology (Carlton). 2014;19:318-24.

362. Garcia de Guadiana-Romualdo L, Berger M, Jimenez-Santos E, Rebollo-Acebes S, Jimenez-Sanchez R, Esteban-Torrella P et al. Pancreatic stone protein and soluble CD25 for infection and sepsis in an emergency department. Eur J Clin Invest. 2017;47:297-304.

363. Llewelyn MJ, Berger M, Gregory M, Ramaiah R, Taylor AL, Curdt I et al. Sepsis biomarkers in unselected patients on admission to intensive or high-dependency care. Crit Care. 2013;17:R60.

364. Peteiro-Cartelle FJ, Alvarez-Jorge A. Dynamic profiles of interleukin-6 and the soluble form of CD25 in burned patients. Burns. 1999;25:487-91.

365. Saito K, Wagatsuma T, Toyama H, Ejima Y, Hoshi K, Shibusawa M et al. Sepsis is characterized by the increases in percentages of circulating CD4+CD25+ regulatory T cells and plasma levels of soluble CD25. Tohoku J Exp Med. 2008;216:61-8.

366. van Ierssel SH, Van Craenenbroeck EM, Hoymans VY, Vrints CJ, Conraads VM, Jorens PG. Endothelium dependent vasomotion and in vitro markers of endothelial repair in patients with severe sepsis: an observational study. PLoS One. 2013;8:e69499.

367. Hur J, Yang HM, Yoon CH, Lee CS, Park KW, Kim JH et al. Identification of a novel role of T cells in postnatal vasculogenesis: characterization of endothelial progenitor cell colonies. Circulation. 2007;116:1671-82.

368. Huang H, Xu R, Lin F, Bao C, Wang S, Ji C et al. High circulating CD39(+) regulatory T cells predict poor survival for sepsis patients. Int J Infect Dis. 2015;30:57-63.

369. Takenaka MC, Robson S, Quintana FJ. Regulation of the T cell response by CD39. Trends Immunol. 2016;37:427-39.

370. Woth G, Tokes-Fuzesi M, Magyarlaki T, Kovacs GL, Vermes I, Muhl D. Activated platelet-derived microparticle numbers are elevated in patients with severe fungal (Candida albicans) sepsis. Ann Clin Biochem. 2012;49:554-60.

371. Gawaz M, Fateh-Moghadam S, Pilz G, Gurland HJ, Werdan K. Platelet activation and interaction with leucocytes in patients with sepsis or multiple organ failure. Eur J Clin Invest. 1995;25:843-51.

372. Katsuura M, Shimizu Y, Akiba K, Kanazawa C, Mitsui T, Sendo D et al. CD48 expression on leukocytes in infectious diseases: flow cytometric analysis of surface antigen. Acta Paediatr Jpn. 1998;40:580-5.

373. McArdel SL, Terhorst C, Sharpe AH. Roles of CD48 in regulating immunity and tolerance. Clin Immunol. 2016;164:10-20.

374. Sharpe AH, Freeman GJ. The B7-CD28 superfamily. Nat Rev Immunol. 2002;2:116-26.

375. Monneret G, Voirin N, Krawice-Radanne I, Bohe J, Lepape A, Rouas-Freiss N et al. Soluble human leukocyte antigen-G5 in septic shock: marked and persisting elevation as a predictor of survival. Crit Care Med. 2007;35:1942-7.

376. Urbonas V, Eidukaite A, Tamuliene I. The predictive value of soluble biomarkers (CD14 subtype, interleukin-2 receptor, human leucocyte antigen-G) and procalcitonin in the detection of bacteremia and sepsis in pediatric oncology patients with chemotherapy-induced febrile neutropenia. Cytokine. 2013;62:34-7.

377. LeMaoult J, Rouas-Freiss N, Carosella ED. Immuno-tolerogenic functions of HLA-G: relevance in transplantation and oncology. Autoimmun Rev. 2005;4:503-9.

378. Cajander S, Rasmussen G, Tina E, Magnuson A, Soderquist B, Kallman J et al. Dynamics of monocytic HLA-DR expression differs between bacterial etiologies during the course of bloodstream infection. PLoS One. 2018;13:e0192883.

379. Cazalis MA, Friggeri A, Cave L, Demaret J, Barbalat V, Cerrato E et al. Decreased HLA-DR antigen-associated invariant chain (CD74) mRNA expression predicts mortality after septic shock. Crit Care. 2013;17:R287.

380. Monneret G, Lepape A, Voirin N, Bohe J, Venet F, Debard AL et al. Persisting low monocyte human leukocyte antigen-DR expression predicts mortality in septic shock. Intensive Care Med. 2006;32:1175-83.

381. Ng PC, Li G, Chui KM, Chu WC, Li K, Wong RP et al. Quantitative measurement of monocyte HLA-DR expression in the identification of early-onset neonatal infection. Biol Neonate. 2006;89:75-81.

382. Vester H, Dargatz P, Huber-Wagner S, Biberthaler P, van Griensven M. HLA-DR expression on monocytes is decreased in polytraumatized patients. Eur J Med Res. 2015;20:84.

383. Schwulst SJ, Muenzer JT, Chang KC, Brahmbhatt TS, Coopersmith CM, Hotchkiss RS. Lymphocyte phenotyping to distinguish septic from nonseptic critical illness. J Am Coll Surg. 2008;206:335-42.

384. Ziegler SF, Ramsdell F, Alderson MR. The activation antigen CD69. Stem Cells. 1994;12:456-65.

385. Figueras-Aloy J, Gomez-Lopez L, Rodriguez-Miguelez JM, Salvia-Roiges MD, Jordan-Garcia I, Ferrer-Codina I et al. Serum soluble ICAM-1, VCAM-1, L-selectin, and P-selectin levels as

markers of infection and their relation to clinical severity in neonatal sepsis. Am J Perinatol. 2007;24:331-8.

386. Kourtis AP, Lee FK, Stoll BJ. Soluble L-selectin, a marker of immune activation, in neonatal infection. Clin Immunol. 2003;109:224-8.

387. Seidelin JB, Nielsen OH, Strom J. Soluble L-selectin levels predict survival in sepsis. Intensive Care Med. 2002;28:1613-8.

388. Tulleken JE, Fijen JW, van der Werf TS, Ligtenberg JJ, Zijlstra JG. Soluble L-selectin levels in experimental human endotoxemia. Crit Care Med. 2001;29:1652-3.

389. Simon SI, Burns AR, Taylor AD, Gopalan PK, Lynam EB, Sklar LA et al. L-selectin (CD62L) cross-linking signals neutrophil adhesive functions via the Mac-1 (CD11b/CD18) beta 2-integrin. J Immunol. 1995;155:1502-14.

390. Zhang L, Qiu C, Yang L, Zhang Z, Zhang Q, Wang B et al. GPR18 expression on PMNs as biomarker for outcome in patient with sepsis. Life Sci. 2019;217:49-56.

391. Kovach MA, Standiford TJ. The function of neutrophils in sepsis. Curr Opin Infect Dis. 2012;25:321-7.

392. Dhainaut JF, Shorr AF, Macias WL, Kollef MJ, Levi M, Reinhart K et al. Dynamic evolution of coagulopathy in the first day of severe sepsis: relationship with mortality and organ failure. Crit Care Med. 2005;33:341-8.

393. Iba T, Kidokoro A, Fukunaga M, Sugiyama K, Sawada T, Kato H. Association between the severity of sepsis and the changes in hemostatic molecular markers and vascular endothelial damage markers. Shock. 2005;23:25-9.

394. Pettila V, Pentti J, Pettila M, Takkunen O, Jousela I. Predictive value of antithrombin III and serum C-reactive protein concentration in critically ill patients with suspected sepsis. Crit Care Med. 2002;30:271-5.

395. Matsubara T, Yamakawa K, Umemura Y, Gando S, Ogura H, Shiraishi A et al. Significance of plasma fibrinogen level and antithrombin activity in sepsis: A multicenter cohort study using a cubic spline model. Thromb Res. 2019;181:17-23.

396. Wiedermann CJ. Clinical review: molecular mechanisms underlying the role of antithrombin in sepsis. Crit Care. 2006;10:209.

397. Hussain N, Hodson D, Marcus R, Baglin T, Luddington R. The biphasic transmittance waveform: an early marker of sepsis in patients with neutropenia. Thromb Haemost. 2008;100:146-8.

398. Zakariah AN, Cozzi SM, Van Nuffelen M, Clausi CM, Pradier O, Vincent JL. Combination of biphasic transmittance waveform with blood procalcitonin levels for diagnosis of sepsis in acutely ill patients. Crit Care Med. 2008;36:1507-12.

399. Toh CH, Ticknor LO, Downey C, Giles AR, Paton RC, Wenstone R. Early identification of sepsis and mortality risks through simple, rapid clot-waveform analysis. Implications of lipoprotein-complexed C reactive protein formation. Intensive Care Med. 2003;29:55-61.

400. Kinasewitz GT, Yan SB, Basson B, Comp P, Russell JA, Cariou A et al. Universal changes in biomarkers of coagulation and inflammation occur in patients with severe sepsis, regardless of causative micro-organism [ISRCTN74215569]. Crit Care. 2004;8:R82-R90.

401. Lee JW, Her SM, Kim JH, Lee KH, Eisenhut M, Park SJ et al. D-dimer as a marker of acute pyelonephritis in infants younger than 24 months with urinary tract infection. Pediatr Nephrol. 2018;33:631-7.

402. Rodelo JR, De la Rosa G, Valencia ML, Ospina S, Arango CM, Gomez CI et al. D-dimer is a significant prognostic factor in patients with suspected infection and sepsis. Am J Emerg Med. 2012;30:1991-9.

403. Koyama K, Madoiwa S, Nunomiya S, Koinuma T, Wada M, Sakata A et al. Combination of thrombin-antithrombin complex, plasminogen activator inhibitor-1, and protein C activity for early identification of severe coagulopathy in initial phase of sepsis: a prospective observational study. Crit Care. 2014;18:R13.

404. Psuja P, Zozulinska M, Turowiecka Z, Cieslikowski W, Vinazzer H, Zawilska K. Plasma markers of hypercoagulability in patients with serious infections and risk of septic shock. Clin Appl Thromb Hemost. 2002;8:225-30.

405. Prisco D. Markers of increased thrombin generation. Ric Clin Lab. 1990;20:217-25.

406. Deitcher SR, Eisenberg PR. Elevated concentrations of cross-linked fibrin degradation products in plasma. An early marker of gram-negative bacteremia. Chest. 1993;103:1107-12.

407. Toh JM, Ken-Dror G, Downey C, Abrams ST. The clinical utility of fibrin-related biomarkers in sepsis. Blood Coagul Fibrinolysis. 2013;24:839-43.

408. Sato N, Takahashi H, Shibata A. Fibrinogen/fibrin degradation products and D-dimer in clinical practice: interpretation of discrepant results. Am J Hematol. 1995;48:168-74.

409. Lorente L, Martin MM, Borreguero-Leon JM, Sole-Violan J, Ferreres J, Labarta L et al. Sustained high plasma plasminogen activator inhibitor-1 levels are associated with severity and mortality in septic patients. Thromb Res. 2014;134:182-6.

410. Madoiwa S, Nunomiya S, Ono T, Shintani Y, Ohmori T, Mimuro J et al. Plasminogen activator inhibitor 1 promotes a poor prognosis in sepsis-induced disseminated intravascular coagulation. Int J Hematol. 2006;84:398-405.

411. Pralong G, Calandra T, Glauser MP, Schellekens J, Verhoef J, Bachmann F et al. Plasminogen activator inhibitor 1: a new prognostic marker in septic shock. Thromb Haemost. 1989;61:459-62.

412. Zeerleder S, Schroeder V, Hack CE, Kohler HP, Wuillemin WA. TAFI and PAI-1 levels in human sepsis. Thromb Res. 2006;118:205-12.

413. Bouchard J, Malhotra R, Shah S, Kao YT, Vaida F, Gupta A et al. Levels of protein C and soluble thrombomodulin in critically ill patients with acute kidney injury: a multicenter prospective observational study. PLoS One. 2015;10:e0120770.

414. Fisher CJ, Jr., Yan SB. Protein C levels as a prognostic indicator of outcome in sepsis and related diseases. Crit Care Med. 2000;28:S49-S56.

415. Gutovitz S, Papa L, Jimenez E, Falk J, Wieman L, Sawyer S et al. Protein C as an early biomarker to distinguish pneumonia from sepsis. J Crit Care. 2011;26:330-12.

416. Heuer JG, Sharma GR, Gerlitz B, Zhang T, Bailey DL, Ding C et al. Evaluation of protein C and other biomarkers as predictors of mortality in a rat cecal ligation and puncture model of sepsis. Crit Care Med. 2004;32:1570-8.

417. Karamarkovic A, Radenkovic D, Milic N, Bumbasirevic V, Stefanovic B. Protein C as an early marker of severe septic complications in diffuse secondary peritonitis. World J Surg. 2005;29:759-65.

418. Kassam A, Chan AK, Dzolganovski B, Constantin J, Ramphal R, Grant R et al. No association between protein C levels and bacteremia in children with febrile neutropenia. J Pediatr Hematol Oncol. 2009;31:647-50.

419. Lauterbach R, Pawlik D, Radziszewska R, Wozniak J, Rytlewski K. Plasma antithrombin III and protein C levels in early recognition of late-onset sepsis in newborns. Eur J Pediatr. 2006;165:585-9.

420. Samransamruajkit R, Hiranrat T, Prapphal N, Sritippayawan S, Deerojanawong J, Poovorawan Y. Levels of protein C activity and clinical factors in early phase of pediatric septic shock may be associated with the risk of death. Shock. 2007;28:518-23.

421. Dettenmeier P, Swindell B, Stroud M, Arkins N, Howard A. Role of activated protein C in the pathophysiology of severe sepsis. Am J Crit Care. 2003;12:518-24.

422. Koyama K, Madoiwa S, Tanaka S, Koinuma T, Wada M, Sakata A et al. Evaluation of hemostatic biomarker abnormalities that precede platelet count decline in critically ill patients with sepsis. J Crit Care. 2013;28:556-63.

423. de Boer JP, Creasey AA, Chang A, Abbink JJ, Roem D, Eerenberg AJ et al. Alpha-2-macroglobulin functions as an inhibitor of fibrinolytic, clotting, and neutrophilic proteinases in sepsis: studies using a baboon model. Infect Immun. 1993;61:5035-43.

424. Juutilainen A, Hamalainen S, Pulkki K, Kuittinen T, Nousiainen T, Jantunen E et al. Biomarkers for bacteremia and severe sepsis in hematological patients with neutropenic fever: multivariate logistic regression analysis and factor analysis. Leuk Lymphoma. 2011;52:2349-55.

425. Wada H, Mori Y, Okabayashi K, Gabazza EC, Kushiya F, Watanabe M et al. High plasma fibrinogen level is associated with poor clinical outcome in DIC patients. Am J Hematol. 2003;72:1-7.

426. Adamzik M, Eggmann M, Frey UH, Gorlinger K, Brocker-Preuss M, Marggraf G et al. Comparison of thromboelastometry with procalcitonin, interleukin 6, and C-reactive protein as diagnostic tests for severe sepsis in critically ill adults. Crit Care. 2010;14:R178.

427. Schochl H, Solomon C, Schulz A, Voelckel W, Hanke A, van Griensven M et al. Thromboelastometry (TEM) findings in disseminated intravascular coagulation in a pig model of endotoxinemia. Mol Med. 2011;17:266-72.

428. Kocak U, Ezer U, Vidinlisan S. Serum fibronectin in neonatal sepsis: is it valuable in early diagnosis and outcome prediction? Acta Paediatr Jpn. 1997;39:428-32.

429. Ruiz Martin G, Prieto PJ, Veiga de Cabo J, Gomez LL, Barberan J, Gonzalez Landa JM et al. Plasma fibronectin as a marker of sepsis. Int J Infect Dis. 2004;8:236-43.

430. Proctor RA. Fibronectin: an enhancer of phagocyte function. Rev Infect Dis. 1987;9 Suppl 4:S412-S419.

431. Riordan FA, Bestwick K, Thomson AP, Sills JA, Hart CA. Plasma fibronectin levels in meningococcal disease. Eur J Pediatr. 1997;156:451-3.

432. To WS, Midwood KS. Plasma and cellular fibronectin: distinct and independent functions during tissue repair. Fibrogenesis Tissue Repair. 2011;4:21.

433. Aibar J, Castro P, Espinosa G, Fernandez S, Hernandez C, Rinaudo M et al. ADAMTS-13 in critically ill patients with septic syndromes and noninfectious systemic inflammatory response syndrome. Shock. 2015;43:556-62.

434. Azfar MF, Khan MF, Habib SS, Aseri ZA, Zubaidi AM, Aguila DO et al. Prognostic value of ADAMTS13 in patients with severe sepsis and septic shock. Clin Invest Med. 2017;40:E49-E58.

435. Lin JJ, Chan OW, Hsiao HJ, Wang Y, Hsia SH, Chiu CH. Decreased ADAMTS 13 activity is associated with disease severity and outcome in pediatric severe sepsis. Medicine (Baltimore). 2016;95:e3374.

436. Martin K, Borgel D, Lerolle N, Feys HB, Trinquart L, Vanhoorelbeke K et al. Decreased ADAMTS-13 (A disintegrin-like and metalloprotease with thrombospondin type 1 repeats) is associated with a poor prognosis in sepsis-induced organ failure. Crit Care Med. 2007;35:2375-82.

437. Mimuro J, Niimura M, Kashiwakura Y, Ishiwata A, Ono T, Ohmori T et al. Unbalanced expression of ADAMTS13 and von Willebrand factor in mouse endotoxinemia. Thromb Res. 2008;122:91-7.

438. Ono T, Mimuro J, Madoiwa S, Soejima K, Kashiwakura Y, Ishiwata A et al. Severe secondary deficiency of von Willebrand factor-cleaving protease (ADAMTS13) in patients with sepsis-

induced disseminated intravascular coagulation: its correlation with development of renal failure. Blood. 2006;107:528-34.

439. Peigne V, Azoulay E, Coquet I, Mariotte E, Darmon M, Legendre P et al. The prognostic value of ADAMTS13 (a disintegrin and metalloprotease with thrombospondin type 1 repeats, member 13) deficiency in septic shock patients involves interleukin-6 and is not dependent on disseminated intravascular coagulation. Crit Care. 2013;17:R273.

440. Zhang Q, Li CS. Risk stratification and prognostic evaluation of endothelial cell-specific molecule1, von Willebrand factor, and a disintegrin-like and metalloprotease with thrombospondin type 1 motif for sepsis in the emergency department: An observational study. Exp Ther Med. 2019;17:4527-35.

441. Furlan M, Robles R, Galbusera M, Remuzzi G, Kyrle PA, Brenner B et al. von Willebrand factor-cleaving protease in thrombotic thrombocytopenic purpura and the hemolytic-uremic syndrome. N Engl J Med. 1998;339:1578-84.

442. Novotny MJ, Turrentine MA, Johnson GS, Adams HR. Experimental endotoxemia increases plasma von Willebrand factor antigen concentrations in dogs with and without free-radical scavenger therapy. Circ Shock. 1987;23:205-13.

443. Rubin DB, Wiener-Kronish JP, Murray JF, Green DR, Turner J, Luce JM et al. Elevated von Willebrand factor antigen is an early plasma predictor of acute lung injury in nonpulmonary sepsis syndrome. J Clin Invest. 1990;86:474-80.

444. Ware LB, Eisner MD, Thompson BT, Parsons PE, Matthay MA. Significance of von Willebrand factor in septic and nonseptic patients with acute lung injury. Am J Respir Crit Care Med. 2004;170:766-72.

445. Federici AB. The factor VIII/von Willebrand factor complex: basic and clinical issues. Haematologica. 2003;88:EREP02.

446. Delabranche X, Boisrame-Helms J, Asfar P, Berger A, Mootien Y, Lavigne T et al. Microparticles are new biomarkers of septic shock-induced disseminated intravascular coagulopathy. Intensive Care Med. 2013;39:1695-703.

447. Soriano AO, Jy W, Chirinos JA, Valdivia MA, Velasquez HS, Jimenez JJ et al. Levels of endothelial and platelet microparticles and their interactions with leukocytes negatively correlate with organ dysfunction and predict mortality in severe sepsis. Crit Care Med. 2005;33:2540-6.

448. Boscolo A, Campello E, Bertini D, Spiezia L, Lucchetta V, Piasentini E et al. Levels of circulating microparticles in septic shock and sepsis-related complications: a case-control study. Minerva Anestesiol. 2019;85:625-34.

449. Meziani F, Delabranche X, Asfar P, Toti F. Bench-to-bedside review: circulating microparticles--a new player in sepsis? Crit Care. 2010;14:236.

450. Nakamura T, Ebihara I, Shoji H, Ushiyama C, Suzuki S, Koide H. Treatment with polymyxin B-immobilized fiber reduces platelet activation in septic shock patients: decrease in plasma levels of soluble P-selectin, platelet factor 4 and beta-thromboglobulin. Inflamm Res. 1999;48:171-5.

451. Kaplan KL, Owen J. Plasma levels of beta-thromboglobulin and platelet factor 4 as indices of platelet activation in vivo. Blood. 1981;57:199-202.

452. Mavrommatis AC, Theodoridis T, Economou M, Kotanidou A, El Ali M, Christopoulou-Kokkinou V et al. Activation of the fibrinolytic system and utilization of the coagulation inhibitors in sepsis: comparison with severe sepsis and septic shock. Intensive Care Med. 2001;27:1853-9.

453. Montes R, Paramo JA, Angles-Cano E, Rocha E. Development and clinical application of a new ELISA assay to determine plasmin-alpha2-antiplasmin complexes in plasma. Br J Haematol. 1996;92:979-85.

454. Bojic S, Kotur-Stevuljevic J, Kalezic N, Stevanovic P, Jelic-Ivanovic Z, Bilanovic D et al. Diagnostic value of matrix metalloproteinase-9 and tissue inhibitor of matrix metalloproteinase-1 in sepsis-associated acute kidney injury. Tohoku J Exp Med. 2015;237:103-9.

455. Hastbacka J, Linko R, Tervahartiala T, Varpula T, Hovilehto S, Parviainen I et al. Serum MMP-8 and TIMP-1 in critically ill patients with acute respiratory failure: TIMP-1 is associated with increased 90-day mortality. Anesth Analg. 2014;118:790-8.

456. Hoffmann U, Bertsch T, Dvortsak E, Liebetrau C, Lang S, Liebe V et al. Matrix-metalloproteinases and their inhibitors are elevated in severe sepsis: prognostic value of TIMP-1 in severe sepsis. Scand J Infect Dis. 2006;38:867-72.

457. Lorente L, Martin MM, Labarta L, Diaz C, Sole-Violan J, Blanquer J et al. Matrix metalloproteinase-9, -10, and tissue inhibitor of matrix metalloproteinases-1 blood levels as biomarkers of severity and mortality in sepsis. Crit Care. 2009;13:R158.

458. Song J, Hu D, He C, Wang T, Liu X, Ma L et al. Novel biomarkers for early prediction of sepsis-induced disseminated intravascular coagulation in a mouse cecal ligation and puncture model. J Inflamm (Lond). 2013;10:7.

459. Xue M, Sun Z, Shao M, Yin J, Deng Z, Zhang J et al. Diagnostic and prognostic utility of tissue factor for severe sepsis and sepsis-induced acute lung injury. J Transl Med. 2015;13:172.

460. Edgington TS, Mackman N, Brand K, Ruf W. The structural biology of expression and function of tissue factor. Thromb Haemost. 1991;66:67-79.

461. Lorenz R, Brauer M. Platelet factor 4 (PF4) in septicaemia. Infection. 1988;16:273-6.

462. Mor-Cohen R, Zucker M, Grissom C, Brown SM, Seligsohn U, Campbell RA et al. The reduced form of coagulation factor XI is associated with illness severity and coagulopathy in critically-ill septic patients. J Thromb Thrombolysis. 2019;47:186-91.

463. Giannakopoulos B, Gao L, Qi M, Wong JW, Yu DM, Vlachoyiannopoulos PG et al. Factor XI is a substrate for oxidoreductases: enhanced activation of reduced FXI and its role in antiphospholipid syndrome thrombosis. J Autoimmun. 2012;39:121-9.

464. Calfee CS, Gallagher D, Abbott J, Thompson BT, Matthay MA. Plasma angiopoietin-2 in clinical acute lung injury: prognostic and pathogenetic significance. Crit Care Med. 2012;40:1731-7.

465. Fang Y, Li C, Shao R, Yu H, Zhang Q, Zhao L. Prognostic significance of the angiopoietin-2/angiopoietin-1 and angiopoietin-1/Tie-2 ratios for early sepsis in an emergency department. Crit Care. 2015;19:367.

466. Fisher J, Douglas JJ, Linder A, Boyd JH, Walley KR, Russell JA. Elevated plasma angiopoietin-2 Levels are associated with fluid overload, organ dysfunction, and mortality in human septic shock. Crit Care Med. 2016;44:2018-27.

467. Kumpers P, van Meurs M, David S, Molema G, Bijzet J, Lukasz A et al. Time course of angiopoietin-2 release during experimental human endotoxemia and sepsis. Crit Care. 2009;13:R64.

468. Luz Fiusa MM, Costa-Lima C, de Souza GR, Vigorito AC, Penteado Aranha FJ, Lorand-Metze I et al. A high angiopoietin-2/angiopoietin-1 ratio is associated with a high risk of septic shock in patients with febrile neutropenia. Crit Care. 2013;17:R169.

469. Orfanos SE, Kotanidou A, Glynos C, Athanasiou C, Tsigkos S, Dimopoulou I et al. Angiopoietin-2 is increased in severe sepsis: correlation with inflammatory mediators. Crit Care Med. 2007;35:199-206.

470. Ricciuto DR, dos Santos CC, Hawkes M, Toltl LJ, Conroy AL, Rajwans N et al. Angiopoietin-1 and angiopoietin-2 as clinically informative prognostic biomarkers of morbidity and mortality in severe sepsis. Crit Care Med. 2011;39:702-10.

471. Wang K, Bhandari V, Giuliano JS, Jr., CS OH, Shattuck MD, Kirby M. Angiopoietin-1, angiopoietin-2 and bicarbonate as diagnostic biomarkers in children with severe sepsis. PLoS One. 2014;9:e108461.

472. Pierce RW, Shabanova V, Canarie M, Pinto M, da Silva YS, Bhandari V et al. Angiopoietin level trajectories in toddlers with severe sepsis and septic shock and their effect on capillary endothelium. Shock. 2019;51:298-305.

473. Fukuhara S, Sako K, Noda K, Zhang J, Minami M, Mochizuki N. Angiopoietin-1/Tie2 receptor signaling in vascular quiescence and angiogenesis. Histol Histopathol. 2010;25:387-96.

474. Scharpfenecker M, Fiedler U, Reiss Y, Augustin HG. The Tie-2 ligand angiopoietin-2 destabilizes quiescent endothelium through an internal autocrine loop mechanism. J Cell Sci. 2005;118:771-80.

475. Boldt J, Muller M, Kuhn D, Linke LC, Hempelmann G. Circulating adhesion molecules in the critically ill: a comparison between trauma and sepsis patients. Intensive Care Med. 1996;22:122-8.

476. Redl H, Dinges HP, Buurman WA, van der Linden CJ, Pober JS, Cotran RS et al. Expression of endothelial leukocyte adhesion molecule-1 in septic but not traumatic/hypovolemic shock in the baboon. Am J Pathol. 1991;139:461-6.

477. Becchi C, Pillozzi S, Fabbri LP, Al MM, Cacciapuoti C, Della BC et al. The increase of endothelial progenitor cells in the peripheral blood: a new parameter for detecting onset and severity of sepsis. Int J Immunopathol Pharmacol. 2008;21:697-705.

478. Rafat N, Hanusch C, Brinkkoetter PT, Schulte J, Brade J, Zijlstra JG et al. Increased circulating endothelial progenitor cells in septic patients: correlation with survival. Crit Care Med. 2007;35:1677-84.

479. Hill JM, Zalos G, Halcox JP, Schenke WH, Waclawiw MA, Quyyumi AA et al. Circulating endothelial progenitor cells, vascular function, and cardiovascular risk. N Engl J Med. 2003;348:593-600.

480. Amalakuhan B, Habib SA, Mangat M, Reyes LF, Rodriguez AH, Hinojosa CA et al. Endothelial adhesion molecules and multiple organ failure in patients with severe sepsis. Cytokine. 2016;88:267-73.

481. Edgar JD, Gabriel V, Gallimore JR, McMillan SA, Grant J. A prospective study of the sensitivity, specificity and diagnostic performance of soluble intercellular adhesion molecule 1, highly sensitive C-reactive protein, soluble E-selectin and serum amyloid A in the diagnosis of neonatal infection. BMC Pediatr. 2010;10:22.

482. Kayal S, Jais JP, Aguini N, Chaudiere J, Labrousse J. Elevated circulating E-selectin, intercellular adhesion molecule 1, and von Willebrand factor in patients with severe infection. Am J Respir Crit Care Med. 1998;157:776-84.

483. Laudes IJ, Guo RF, Riedemann NC, Speyer C, Craig R, Sarma JV et al. Disturbed homeostasis of lung intercellular adhesion molecule-1 and vascular cell adhesion molecule-1 during sepsis. Am J Pathol. 2004;164:1435-45.

484. Mahmoud S, Maklad S, Abo Elmagd EK. Soluble intercellular adhesion molecule-1 (slCAM-1) for early diagnosis of neonatal infections. Egypt J Immunol. 2009;16:83-94.

485. Sosa-Bustamante GP, Amador-Licona N, Barbosa-Sabanero G, Guizar-Mendoza JM, Lopez-Briones S, Mulgado-Aguas CI et al. Intercellular adhesion molecules and mortality for sepsis in infants younger than 1 year of life. Rev Invest Clin. 2011;63:601-6.

486. Weigand MA, Schmidt H, Pourmahmoud M, Zhao Q, Martin E, Bardenheuer HJ. Circulating intercellular adhesion molecule-1 as an early predictor of hepatic failure in patients with septic shock. Crit Care Med. 1999;27:2656-61.

487. Smith CW, Rothlein R, Hughes BJ, Mariscalco MM, Rudloff HE, Schmalstieg FC et al. Recognition of an endothelial determinant for CD 18-dependent human neutrophil adherence and transendothelial migration. J Clin Invest. 1988;82:1746-56.

488. Su CM, Cheng HH, Tsai TC, Hsiao SY, Tsai NW, Chang WN et al. Elevated serum vascular cell adhesion molecule-1 is associated with septic encephalopathy in adult community-onset severe sepsis patients. Biomed Res Int. 2014;2014:598762.

489. van Oosten M, van de Bilt E, de Vries HE, van Berkel TJ, Kuiper J. Vascular adhesion molecule-1 and intercellular adhesion molecule-1 expression on rat liver cells after lipopolysaccharide administration in vivo. Hepatology. 1995;22:1538-46.

490. Whalen MJ, Doughty LA, Carlos TM, Wisniewski SR, Kochanek PM, Carcillo JA. Intercellular adhesion molecule-1 and vascular cell adhesion molecule-1 are increased in the plasma of children with sepsis-induced multiple organ failure. Crit Care Med. 2000;28:2600-7.

491. Elices MJ, Osborn L, Takada Y, Crouse C, Luhowskyj S, Hemler ME et al. VCAM-1 on activated endothelium interacts with the leukocyte integrin VLA-4 at a site distinct from the VLA-4/fibronectin binding site. Cell. 1990;60:577-84.

492. Sallisalmi M, Tenhunen J, Yang R, Oksala N, Pettila V. Vascular adhesion protein-1 and syndecan-1 in septic shock. Acta Anaesthesiol Scand. 2012;56:316-22.

493. Pannecoeck R, Serruys D, Benmeridja L, Delanghe JR, van Geel N, Speeckaert R et al. Vascular adhesion protein-1: Role in human pathology and application as a biomarker. Crit Rev Clin Lab Sci. 2015;52:284-300.

494. Presterl E, Lassnigg A, Mueller-Uri P, Wenisch C, El-Menyawi I, Graninger W. High serum laminin concentrations in patients with Candida sepsis. Eur J Clin Invest. 1999;29:992-6.

495. Johansen ME, Johansson PI, Ostrowski SR, Bestle MH, Hein L, Jensen AL et al. Profound endothelial damage predicts impending organ failure and death in sepsis. Semin Thromb Hemost. 2015;41:16-25.

496. Puskarich MA, Cornelius DC, Tharp J, Nandi U, Jones AE. Plasma syndecan-1 levels identify a cohort of patients with severe sepsis at high risk for intubation after large-volume intravenous fluid resuscitation. J Crit Care. 2016;36:125-9.

497. Wei S, Gonzalez Rodriguez E, Chang R, Holcomb JB, Kao LS, Wade CE. Elevated syndecan-1 after trauma and risk of sepsis: A secondary analysis of patients from the Pragmatic, Randomized Optimal Platelet and Plasma Ratios (PROPPR) trial. J Am Coll Surg. 2018;227:587-95.

498. Smart L, Bosio E, Macdonald SPJ, Dull R, Fatovich DM, Neil C et al. Glycocalyx biomarker syndecan-1 is a stronger predictor of respiratory failure in patients with sepsis due to pneumonia, compared to endocan. J Crit Care. 2018;47:93-8.

499. Rehm M, Bruegger D, Christ F, Conzen P, Thiel M, Jacob M et al. Shedding of the endothelial glycocalyx in patients undergoing major vascular surgery with global and regional ischemia. Circulation. 2007;116:1896-906.

500. Masson S, Daveau M, Francois A, Bodenant C, Hiron M, Teniere P et al. Up-regulated expression of HGF in rat liver cells after experimental endotoxemia: a potential pathway for enhancement of liver regeneration. Growth Factors. 2001;18:237-50.

501. Nayeri F, Brudin L, Darelid J, Nilsson I, Fryden A, Soderstrom C et al. Hepatocyte growth factor may act as an early therapeutic predictor in pneumonia. Scand J Infect Dis. 2002;34:500-4.

502. Nayeri F, Nilsson I, Brudin L, Fryden A, Soderstrom C, Forsberg P. High serum hepatocyte growth factor levels in the acute stage of community-acquired infectious diseases. Scand J Infect Dis. 2002;34:127-30.

503. Hayashi S, Morishita R, Higaki J, Aoki M, Moriguchi A, Kida I et al. Autocrine-paracrine effects of overexpression of hepatocyte growth factor gene on growth of endothelial cells. Biochem Biophys Res Commun. 1996;220:539-45.

504. Redl H, Schlag G, Togel E, Assicot M, Bohuon C. Procalcitonin release patterns in a baboon model of trauma and sepsis: relationship to cytokines and neopterin. Crit Care Med. 2000;28:3659-63.

505. Ruokonen E, Ilkka L, Niskanen M, Takala J. Procalcitonin and neopterin as indicators of infection in critically ill patients. Acta Anaesthesiol Scand. 2002;46:398-404.

506. Andert SE, Griesmacher A, Zuckermann A, Muller MM. Neopterin release from human endothelial cells is triggered by interferon-gamma. Clin Exp Immunol. 1992;88:555-8.

507. Brueckmann M, Hoffmann U, Engelhardt C, Lang S, Fukudome K, Haase KK et al. Prognostic value of platelet-derived growth factor in patients with severe sepsis. Growth Factors. 2007;25:15-24.

508. Battegay EJ, Rupp J, Iruela-Arispe L, Sage EH, Pech M. PDGF-BB modulates endothelial proliferation and angiogenesis in vitro via PDGF beta-receptors. J Cell Biol. 1994;125:917-28.

509. van der Flier M, van Leeuwen HJ, van Kessel KP, Kimpen JL, Hoepelman AI, Geelen SP. Plasma vascular endothelial growth factor in severe sepsis. Shock. 2005;23:35-8.

510. Karlsson S, Pettila V, Tenhunen J, Lund V, Hovilehto S, Ruokonen E. Vascular endothelial growth factor in severe sepsis and septic shock. Anesth Analg. 2008;106:1820-6.

511. Kim CO, Huh AJ, Kim MS, Chin BS, Han SH, Choi SH et al. LPS-induced vascular endothelial growth factor expression in rat lung pericytes. Shock. 2008;30:92-7.

512. Yang KY, Liu KT, Chen YC, Chen CS, Lee YC, Perng RP et al. Plasma soluble vascular endothelial growth factor receptor-1 levels predict outcomes of pneumonia-related septic shock patients: a prospective observational study. Crit Care. 2011;15:R11.

513. Whitney JE, Silverman M, Norton JS, Bachur RG, Melendez E. Vascular endothelial growth factor and soluble vascular endothelial growth factor receptor as novel biomarkers for poor outcomes in children with severe sepsis and septic shock. Pediatr Emerg Care. 2018.

514. Gerber HP, McMurtrey A, Kowalski J, Yan M, Keyt BA, Dixit V et al. Vascular endothelial growth factor regulates endothelial cell survival through the phosphatidylinositol 3'-kinase/Akt signal transduction pathway. Requirement for Flk-1/KDR activation. J Biol Chem. 1998;273:30336-43.

515. Cummings CJ, Sessler CN, Beall LD, Fisher BJ, Best AM, Fowler AA, III. Soluble E-selectin levels in sepsis and critical illness. Correlation with infection and hemodynamic dysfunction. Am J Respir Crit Care Med. 1997;156:431-7.

516. De Pablo R, Monserrat J, Reyes E, Diaz D, Rodriguez-Zapata M, de la Hera A et al. Circulating sICAM-1 and sE-Selectin as biomarker of infection and prognosis in patients with systemic inflammatory response syndrome. Eur J Intern Med. 2013;24:132-8.

517. Drake TA, Cheng J, Chang A, Taylor FB, Jr. Expression of tissue factor, thrombomodulin, and E-selectin in baboons with lethal Escherichia coli sepsis. Am J Pathol. 1993;142:1458-70.

518. Su CM, Cheng HH, Hung CW, Hsiao SY, Tsai NW, Chang WN et al. The value of serial serum cell adhesion molecules in predicting acute kidney injury after severe sepsis in adults. Clin Chim Acta. 2016;457:86-91.

519. Chase SD, Magnani JL, Simon SI. E-selectin ligands as mechanosensitive receptors on neutrophils in health and disease. Ann Biomed Eng. 2012;40:849-59.

520. Morlion BJ, Torwesten E, Kuhn KS, Puchstein C, Furst P. Cysteinyl-leukotriene generation as a biomarker for survival in the critically ill. Crit Care Med. 2000;28:3655-8.

521. Dennis EA, Norris PC. Eicosanoid storm in infection and inflammation. Nat Rev Immunol. 2015;15:511-23.

522. Beran O, Herwald H, Dzupova O, Chalupa P, Holub M. Heparin-binding protein as a biomarker of circulatory failure during severe infections: a report of three cases. Scand J Infect Dis. 2010;42:634-6.

523. Linder A, Akesson P, Inghammar M, Treutiger CJ, Linner A, Sunden-Cullberg J. Elevated plasma levels of heparin-binding protein in intensive care unit patients with severe sepsis and septic shock. Crit Care. 2012;16:R90.

524. Linder A, Christensson B, Herwald H, Bjorck L, Akesson P. Heparin-binding protein: an early marker of circulatory failure in sepsis. Clin Infect Dis. 2009;49:1044-50.

525. Linder A, Arnold R, Boyd JH, Zindovic M, Zindovic I, Lange A et al. Heparin-binding protein measurement improves the prediction of severe infection with organ dysfunction in the emergency department. Crit Care Med. 2015;43:2378-86.

526. Liu XW, Ma T, Liu W, Cai Q, Wang L, Song HW et al. Sustained increase in angiopoietin-2, heparin-binding protein, and procalcitonin is associated with severe sepsis. J Crit Care. 2018;45:14-9.

527. Tverring J, Vaara ST, Fisher J, Poukkanen M, Pettila V, Linder A. Heparin-binding protein (HBP) improves prediction of sepsis-related acute kidney injury. Ann Intensive Care. 2017;7:105.

528. Tyden J, Herwald H, Hultin M, Wallden J, Johansson J. Heparin-binding protein as a biomarker of acute kidney injury in critical illness. Acta Anaesthesiol Scand. 2017;61:797-803.

529. Kahn F, Tverring J, Mellhammar L, Wetterberg N, Blackberg A, Studahl E et al. Heparin-binding protein as a prognostic biomarker of sepsis and disease severity at the emergency department. Shock. 2019;52:e135-e145

530. Zhou Y, Liu Z, Huang J, Li G, Li F, Cheng Y et al. Usefulness of the heparin-binding protein level to diagnose sepsis and septic shock according to Sepsis-3 compared with procalcitonin and C reactive protein: a prospective cohort study in China. BMJ Open. 2019;9:e026527.

531. Gautam N, Olofsson AM, Herwald H, Iversen LF, Lundgren-Akerlund E, Hedqvist P et al. Heparin-binding protein (HBP/CAP37): a missing link in neutrophil-evoked alteration of vascular permeability. Nat Med. 2001;7:1123-7.

532. Lee W, Yoo H, Ku SK, Kim SW, Bae JS. Raftlin: a new biomarker in human sepsis. Inflammation. 2014;37:706-11.

533. Bae JS, Yang L, Rezaie AR. Lipid raft localization regulates the cleavage specificity of protease activated receptor 1 in endothelial cells. J Thromb Haemost. 2008;6:954-61.

534. Luchtemberg MN, Petronilho F, Constantino L, Gelain DP, Andrades M, Ritter C et al. Xanthine oxidase activity in patients with sepsis. Clin Biochem. 2008;41:1186-90.

535. Galley HF, Davies MJ, Webster NR. Xanthine oxidase activity and free radical generation in patients with sepsis syndrome. Crit Care Med. 1996;24:1649-53.

536. Lin SM, Wang YM, Lin HC, Lee KY, Huang CD, Liu CY et al. Serum thrombomodulin level relates to the clinical course of disseminated intravascular coagulation, multiorgan dysfunction syndrome, and mortality in patients with sepsis. Crit Care Med. 2008;36:683-9.

537. Mihajlovic DM, Lendak DF, Draskovic BG, Mikic AS, Mitic GP, Cebovic TN et al. Thrombomodulin is a strong predictor of multiorgan dysfunction syndrome in patients with sepsis. Clin Appl Thromb Hemost. 2015;21:469-74.

538. Conway EM. Thrombomodulin and its role in inflammation. Semin Immunopathol. 2012;34:107-25.

539. Hsiao SY, Kung CT, Tsai NW, Su CM, Huang CC, Lai YR et al. Concentration and value of endocan on outcome in adult patients after severe sepsis. Clin Chim Acta. 2018;483:275-80.

540. Mihajlovic DM, Lendak DF, Brkic SV, Draskovic BG, Mitic GP, Novakov Mikic AS et al. Endocan is useful biomarker of survival and severity in sepsis. Microvasc Res. 2014;93:92-7.

541. Pauly D, Hamed S, Behnes M, Lepiorz D, Lang S, Akin I et al. Endothelial cell-specific molecule-1/endocan: Diagnostic and prognostic value in patients suffering from severe sepsis and septic shock. J Crit Care. 2016;31:68-75.

542. Perrotti A, Chenevier-Gobeaux C, Ecarnot F, Barrucand B, Lassalle P, Dorigo E et al. Relevance of endothelial cell-specific molecule 1 (endocan) plasma levels for predicting pulmonary infection after cardiac surgery in chronic kidney disease patients: The Endolung Pilot Study. Cardiorenal Med. 2017;8:1-8.

543. Scherpereel A, Depontieu F, Grigoriu B, Cavestri B, Tsicopoulos A, Gentina T et al. Endocan, a new endothelial marker in human sepsis. Crit Care Med. 2006;34:532-7.

544. Tissier S, Lancel S, Marechal X, Mordon S, Depontieu F, Scherpereel A et al. Calpain inhibitors improve myocardial dysfunction and inflammation induced by endotoxin in rats. Shock. 2004;21:352-7.

545. Sarrazin S, Adam E, Lyon M, Depontieu F, Motte V, Landolfi C et al. Endocan or endothelial cell specific molecule-1 (ESM-1): a potential novel endothelial cell marker and a new target for cancer therapy. Biochim Biophys Acta. 2006;1765:25-37.

546. De Freitas Caires N, Legendre B, Parmentier E, Scherpereel A, Tsicopoulos A, Mathieu D et al. Identification of a 14 kDa endocan fragment generated by cathepsin G, a novel circulating biomarker in patients with sepsis. J Pharm Biomed Anal. 2013;78-79:45-51.

547. Lundberg OH, Bergenzaun L, Ryden J, Rosenqvist M, Melander O, Chew MS. Adrenomedullin and endothelin-1 are associated with myocardial injury and death in septic shock patients. Crit Care. 2016;20:178.

548. Balcan B, Olgun S, Akbas T, Eryuksel E, Karakurt S. Level of adrenomedullin in cases with adrenal defficiency and its relation to mortality in patients with sepsis. Tuberk Toraks. 2016;64:191-7.

549. Caironi P, Latini R, Struck J, Hartmann O, Bergmann A, Maggio G et al. Circulating biologically active adrenomedullin (bio-ADM) predicts hemodynamic support requirement and mortality during sepsis. Chest. 2017;152:312-20.

550. Chen YX, Li CS. Prognostic value of adrenomedullin in septic patients in the ED. Am J Emerg Med. 2013;31:1017-21.

551. Christ-Crain M, Morgenthaler NG, Struck J, Harbarth S, Bergmann A, Muller B. Mid-regional pro-adrenomedullin as a prognostic marker in sepsis: an observational study. Crit Care. 2005;9:R816-R824.

552. Demirkaya M, Tugcu D, Akcay A, Aydogan G, Akici F, Salcioglu Z et al. Adrenomedullin--A new marker in febrile neutropenia: Comparison with CRP and procalcitonin. Pediatr Hematol Oncol. 2015;32:482-9.

553. Jiang W, Jiang HF, Cai DY, Pan CS, Qi YF, Pang YZ et al. Relationship between contents of adrenomedullin and distributions of neutral endopeptidase in blood and tissues of rats in septic shock. Regul Pept. 2004;118:199-208.

554. Simon TP, Martin L, Doemming S, Humbs A, Bruells C, Kopp R et al. Plasma adrenomedullin in critically ill patients with sepsis after major surgery: A pilot study. J Crit Care. 2017;38:68-72.

555. Kim H, Hur M, Struck J, Bergmann A, Di Somma S. Circulating biologically active adrenomedullin predicts organ failure and mortality in sepsis. Ann Lab Med. 2019;39:454-63.

556. Nishio K, Akai Y, Murao Y, Doi N, Ueda S, Tabuse H et al. Increased plasma concentrations of adrenomedullin correlate with relaxation of vascular tone in patients with septic shock. Crit Care Med. 1997;25:953-7.

557. Angeletti S, Spoto S, Fogolari M, Cortigiani M, Fioravanti M, De Florio L et al. Diagnostic and prognostic role of procalcitonin (PCT) and MR-pro-Adrenomedullin (MR-proADM) in bacterial infections. APMIS. 2015;123:740-8.

558. Guignant C, Voirin N, Venet F, Poitevin F, Malcus C, Bohe J et al. Assessment of pro-vasopressin and pro-adrenomedullin as predictors of 28-day mortality in septic shock patients. Intensive Care Med. 2009;35:1859-67.

559. Al Shuaibi M, Bahu RR, Chaftari AM, Al Wohoush I, Shomali W, Jiang Y et al. Pro-adrenomedullin as a novel biomarker for predicting infections and response to antimicrobials in febrile patients with hematologic malignancies. Clin Infect Dis. 2013;56:943-50.

560. Angeletti S, Battistoni F, Fioravanti M, Bernardini S, Dicuonzo G. Procalcitonin and mid-regional pro-adrenomedullin test combination in sepsis diagnosis. Clin Chem Lab Med. 2013;51:1059-67.

561. Benito J, Luaces-Cubells C, Mintegi S, Astobiza E, Martinez-Indart L, Valls-Lafont A et al. Lack of value of midregional pro-adrenomedullin and C-terminal pro-endothelin-1 for prediction of severe bacterial infections in infants with fever without a source. Eur J Pediatr. 2013;172:1441-9.

562. Christ-Crain M, Morgenthaler NG, Stolz D, Muller C, Bingisser R, Harbarth S et al. Pro-adrenomedullin to predict severity and outcome in community-acquired pneumonia [ISRCTN04176397]. Crit Care. 2006;10:R96.

563. Travaglino F, De Berardinis B, Magrini L, Bongiovanni C, Candelli M, Silveri NG et al. Utility of procalcitonin (PCT) and mid regional pro-adrenomedullin (MR-proADM) in risk stratification of

critically ill febrile patients in Emergency Department (ED). A comparison with APACHE II score. BMC Infect Dis. 2012;12:184.

564. Viaggi B, Poole D, Tujjar O, Marchiani S, Ognibene A, Finazzi S. Mid regional pro-adrenomedullin for the prediction of organ failure in infection. Results from a single centre study. PLoS One. 2018;13:e0201491.

565. Wang RL, Kang FX. Prediction about severity and outcome of sepsis by pro-atrial natriuretic peptide and pro-adrenomedullin. Chin J Traumatol. 2010;13:152-7.

566. Ni J, Sun Y, Qu H, Wang A, Cao Y, Li X. Prognostic value of serum proadrenomedullin in catheter-related bloodstream infection in the intensive care unit: A prospective observational study. Medicine (Baltimore). 2018;97:e12821.

567. Saeed K, Wilson DC, Bloos F, Schuetz P, van der Does Y, Melander O et al. The early identification of disease progression in patients with suspected infection presenting to the emergency department: a multi-centre derivation and validation study. Crit Care. 2019;23:40.

568. Bahrami S, Pelinka L, Khadem A, Maitzen S, Hawa G, van Griensven M et al. Circulating NT-proCNP predicts sepsis in multiple-traumatized patients without traumatic brain injury. Crit Care Med. 2010;38:161-6.

569. DeClue AE, Osterbur K, Bigio A, Sharp CR. Evaluation of serum NT-pCNP as a diagnostic and prognostic biomarker for sepsis in dogs. J Vet Intern Med. 2011;25:453-9.

570. Hama N, Itoh H, Shirakami G, Suga S, Komatsu Y, Yoshimasa T et al. Detection of C-type natriuretic peptide in human circulation and marked increase of plasma CNP level in septic shock patients. Biochem Biophys Res Commun. 1994;198:1177-82.

571. Koch A, Voigt S, Sanson E, Duckers H, Horn A, Zimmermann HW et al. Prognostic value of circulating amino-terminal pro-C-type natriuretic peptide in critically ill patients. Crit Care. 2011;15:R45.

572. Ehler J, Saller T, Wittstock M, Rommer PS, Chappell D, Zwissler B et al. Diagnostic value of NT-proCNP compared to NSE and S100B in cerebrospinal fluid and plasma of patients with sepsis-associated encephalopathy. Neurosci Lett. 2019;692:167-73.

573. Chauhan SD, Nilsson H, Ahluwalia A, Hobbs AJ. Release of C-type natriuretic peptide accounts for the biological activity of endothelium-derived hyperpolarizing factor. Proc Natl Acad Sci U S A. 2003;100:1426-31.

574. Beer S, Weighardt H, Emmanuilidis K, Harzenetter MD, Matevossian E, Heidecke CD et al. Systemic neuropeptide levels as predictive indicators for lethal outcome in patients with postoperative sepsis. Crit Care Med. 2002;30:1794-8.

575. Lorente L, Martin MM, Almeida T, Hernandez M, Ferreres J, Sole-Violan J et al. Association between serum substance P levels and mortality in patients with severe sepsis. J Crit Care. 2015;30:924-8.

576. Zamir O, Hasselgren PO, Higashiguchi T, Frederick JA, Fischer JE. Effect of sepsis or cytokine administration on release of gut peptides. Am J Surg. 1992;163:181-4.

577. O'Connor TM, O'Connell J, O'Brien DI, Goode T, Bredin CP, Shanahan F. The role of substance P in inflammatory disease. J Cell Physiol. 2004;201:167-80.

578. Galley HF, Le Cras AE, Yassen K, Grant IS, Webster NR. Circulating tetrahydrobiopterin concentrations in patients with septic shock. Br J Anaesth. 2001;86:578-80.

579. Hattori Y, Nakanishi N, Kasai K, Murakami Y, Shimoda S. Tetrahydrobiopterin and GTP cyclohydrolase I in a rat model of endotoxic shock: relation to nitric oxide synthesis. Exp Physiol. 1996;81:665-71.

580. He X, Su F, Velissaris D, Salgado DR, de Souza BD, Lorent S et al. Administration of tetrahydrobiopterin improves the microcirculation and outcome in an ovine model of septic shock. Crit Care Med. 2012;40:2833-40.

581. Brandtzaeg P, Oktedalen O, Kierulf P, Opstad PK. Elevated VIP and endotoxin plasma levels in human gram-negative septic shock. Regul Pept. 1989;24:37-44.

582. Jacob P, Mueller MH, Hahn J, Wolk I, Mayer P, Nagele U et al. Alterations of neuropeptides in the human gut during peritonitis. Langenbecks Arch Surg. 2007;392:267-71.

583. Revhaug A, Lygren I, Lundgren TI, Andersen OK, Burhol PG, Giercksky KE. Increased plasma levels of vasoactive intestinal polypeptide in pigs during endotoxinaemia. Eur Surg Res. 1985;17:75-82.

584. Henning RJ, Sawmiller DR. Vasoactive intestinal peptide: cardiovascular effects. Cardiovasc Res. 2001;49:27-37.

585. Faury G, Wynnychenko TM, Cand F, Leone M, Jacob MP, Verdetti J et al. Decreased circulating elastin peptide levels in humans with sepsis. Pathol Biol (Paris). 2005;53:443-7.

586. Faury G, Ristori MT, Verdetti J, Jacob MP, Robert L. Effect of elastin peptides on vascular tone. J Vasc Res. 1995;32:112-9.

587. de Werra I, Jaccard C, Corradin SB, Chiolero R, Yersin B, Gallati H et al. Cytokines, nitrite/nitrate, soluble tumor necrosis factor receptors, and procalcitonin concentrations: comparisons in patients with septic shock, cardiogenic shock, and bacterial pneumonia. Crit Care Med. 1997;25:607-13.

588. Krafte-Jacobs B, Brilli R, Szabo C, Denenberg A, Moore L, Salzman AL. Circulating methemoglobin and nitrite/nitrate concentrations as indicators of nitric oxide overproduction in critically ill children with septic shock. Crit Care Med. 1997;25:1588-93.

589. van Amsterdam JG, van den Berg C, Zuidema J, te Biesebeek JD, Rokos H. Effect of septicaemia on the plasma levels of biopterin and nitric oxide metabolites in rats and rabbits. Biochem Pharmacol. 1996;52:1447-51.

590. Demirbilek S, Ersoy MO, Demirbilek S, Karaman A, Akin M, Bayraktar M et al. Effects of polyenylphosphatidylcholine on cytokines, nitrite/nitrate levels, antioxidant activity and lipid peroxidation in rats with sepsis. Intensive Care Med. 2004;30:1974-8.

591. Arnalich F, Sanchez JF, Martinez M, Jimenez M, Lopez J, Vazquez JJ et al. Changes in plasma concentrations of vasoactive neuropeptides in patients with sepsis and septic shock. Life Sci. 1995;56:75-81.

592. Arden WA, Fiscus RR, Wang X, Yang L, Maley R, Nielsen M et al. Elevations in circulating calcitonin gene-related peptide correlate with hemodynamic deterioration during endotoxic shock in pigs. Circ Shock. 1994;42:147-53.

593. Ay I, Tuncer M. Mechanism of CGRP-induced vasodilation in the rat isolated perfused kidney. Pharmacology. 2004;71:209-15.

594. Asmis LM, Asmis R, Sulzer I, Furlan M, Lammle B. Contact system activation in human sepsis - 47kD HK, a marker of sepsis severity? Swiss Med Wkly. 2008;138:142-9.

595. Aydemir O, Ozcan B, Yucel H, Bas AY, Demirel N. Asymmetric dimethylarginine and L-arginine levels in neonatal sepsis and septic shock. J Matern Fetal Neonatal Med. 2015;28:977-82.

596. Koch A, Weiskirchen R, Bruensing J, Duckers H, Buendgens L, Kunze J et al. Regulation and prognostic relevance of symmetric dimethylarginine serum concentrations in critical illness and sepsis. Mediators Inflamm. 2013;2013:413826.

597. Lappalainen M, Hamalainen S, Juutilainen A, Koivula I, Pulkki K, Jantunen E. Asymmetric dimethylarginine in the assessment of febrile neutropenia in hematological patients. Scand J Clin Lab Invest. 2017;77:130-4.

598. Juonala M, Viikari JS, Alfthan G, Marniemi J, Kahonen M, Taittonen L et al. Brachial artery flow-mediated dilation and asymmetrical dimethylarginine in the cardiovascular risk in young Finns study. Circulation. 2007;116:1367-73.

599. Tanaka M, Yanagihara I, Takahashi H, Hamaguchi M, Nakahira K, Sakata I. The mRNA expression of fatty acid amide hydrolase in human whole blood correlates with sepsis. J Endotoxin Res. 2007;13:35-8.

600. Fezza F, De Simone C, Amadio D, Maccarrone M. Fatty acid amide hydrolase: a gate-keeper of the endocannabinoid system. Subcell Biochem. 2008;49:101-32.

601. Yoo H, Ku SK, Kim SW, Bae JS. Early diagnosis of sepsis using serum hemoglobin subunit Beta. Inflammation. 2015;38:394-9.

602. Gladwin MT, Ognibene FP, Pannell LK, Nichols JS, Pease-Fye ME, Shelhamer JH et al. Relative role of heme nitrosylation and beta-cysteine 93 nitrosation in the transport and metabolism of nitric oxide by hemoglobin in the human circulation. Proc Natl Acad Sci U S A. 2000;97:9943-8.

603. Rosenberg RB, Broner CW, O'Dorisio MS. Modulation of cyclic guanosine monophosphate production during Escherichia coli septic shock. Biochem Med Metab Biol. 1994;51:149-55.

604. Broner CW, O'Dorisio MS, Rosenberg RB, O'Dorisio TM. Cyclic nucleotides and vasoactive intestinal peptide production in a rabbit model of Escherichia coli septicemia. Am J Med Sci. 1995;309:267-77.

605. Wang X, Jones SB, Zhou Z, Han C, Fiscus RR. Calcitonin gene-related peptide (CGRP) and neuropeptide Y (NPY) levels are elevated in plasma and decreased in vena cava during endotoxin shock in the rat. Circ Shock. 1992;36:21-30.

606. Kennedy B, Shen GH, Ziegler MG. Neuropeptide Y-mediated pressor responses following high-frequency stimulation of the rat sympathetic nervous system. J Pharmacol Exp Ther. 1997;281:291-6.

607. Alqahtani MF, Smith CM, Weiss SL, Dawson S, Ralay RH, Wainwright MS. Evaluation of new diagnostic biomarkers in pediatric sepsis: Matrix metalloproteinase-9, tissue inhibitor of metalloproteinase-1, mid-regional pro-atrial natriuretic peptide, and adipocyte fatty-acid binding protein. PLoS One. 2016;11:e0153645.

608. Claessens YE, Schmidt J, Batard E, Grabar S, Jegou D, Hausfater P et al. Can C-reactive protein, procalcitonin and mid-regional pro-atrial natriuretic peptide measurements guide choice of in-

patient or out-patient care in acute pyelonephritis? Biomarkers In Sepsis (BIS) multicentre study. Clin Microbiol Infect. 2010;16:753-60.

609. Hartemink KJ, Groeneveld AB, de Groot MC, Strack Van Schijndel RJ, van Kamp G, Thijs LG. alpha-atrial natriuretic peptide, cyclic guanosine monophosphate, and endothelin in plasma as markers of myocardial depression in human septic shock. Crit Care Med. 2001;29:80-7.

610. Lipinska-Gediga M, Mierzchala M, Durek G. Pro-atrial natriuretic peptide (pro-ANP) level in patients with severe sepsis and septic shock: prognostic and diagnostic significance. Infection. 2012;40:303-9.

611. Morgenthaler NG, Struck J, Christ-Crain M, Bergmann A, Muller B. Pro-atrial natriuretic peptide is a prognostic marker in sepsis, similar to the APACHE II score: an observational study. Crit Care. 2005;9:R37-R45.

612. Gille J, Schmidt J, Kremer T, Sablotzki A. Evaluation of MR-proANP and copeptin for sepsis diagnosis after burn injury. J Crit Care. 2019;52:149-55.

613. Berendes E, Van Aken H, Raufhake C, Schmidt C, Assmann G, Walter M. Differential secretion of atrial and brain natriuretic peptide in critically ill patients. Anesth Analg. 2001;93:676-82.

614. Charpentier J, Luyt CE, Fulla Y, Vinsonneau C, Cariou A, Grabar S et al. Brain natriuretic peptide: A marker of myocardial dysfunction and prognosis during severe sepsis. Crit Care Med. 2004;32:660-5.

615. Chen Y, Li C. Prognostic significance of brain natriuretic peptide obtained in the ED in patients with SIRS or sepsis. Am J Emerg Med. 2009;27:701-6.

616. Garcia Villalba E, Bernal Morell E, Egea MP, Marin I, Alcaraz Garcia A, Munoz A et al. The N-terminal pro brain natriuretic peptide is the best predictor of mortality during hospitalization in patients with low risk of sepsis-related organ failure. Med Clin (Barc). 2017;149:189-95.

617. Hoffmann U, Brueckmann M, Bertsch T, Wiessner M, Liebetrau C, Lang S et al. Increased plasma levels of NT-proANP and NT-proBNP as markers of cardiac dysfunction in septic patients. Clin Lab. 2005;51:373-9.

618. Hur M, Kim H, Lee S, Cristofano F, Magrini L, Marino R et al. Diagnostic and prognostic utilities of multimarkers approach using procalcitonin, B-type natriuretic peptide, and neutrophil gelatinase-associated lipocalin in critically ill patients with suspected sepsis. BMC Infect Dis. 2014;14:224.

619. Kandil E, Burack J, Sawas A, Bibawy H, Schwartzman A, Zenilman ME et al. B-type natriuretic peptide: a biomarker for the diagnosis and risk stratification of patients with septic shock. Arch Surg. 2008;143:242-6.

620. Khoury J, Arow M, Elias A, Makhoul BF, Berger G, Kaplan M et al. The prognostic value of brain natriuretic peptide (BNP) in non-cardiac patients with sepsis, ultra-long follow-up. J Crit Care. 2017;42:117-22.

621. Klouche K, Pommet S, Amigues L, Bargnoux AS, Dupuy AM, Machado S et al. Plasma brain natriuretic peptide and troponin levels in severe sepsis and septic shock: relationships with systolic myocardial dysfunction and intensive care unit mortality. J Intensive Care Med. 2014;29:229-37.

622. McLean AS, Huang SJ, Hyams S, Poh G, Nalos M, Pandit R et al. Prognostic values of B-type natriuretic peptide in severe sepsis and septic shock. Crit Care Med. 2007;35:1019-26.

623. Papanikolaou J, Makris D, Mpaka M, Palli E, Zygoulis P, Zakynthinos E. New insights into the mechanisms involved in B-type natriuretic peptide elevation and its prognostic value in septic patients. Crit Care. 2014;18:R94.

624. Post F, Weilemann LS, Messow CM, Sinning C, Munzel T. B-type natriuretic peptide as a marker for sepsis-induced myocardial depression in intensive care patients. Crit Care Med. 2008;36:3030-7.

625. Rivers EP, McCord J, Otero R, Jacobsen G, Loomba M. Clinical utility of B-type natriuretic peptide in early severe sepsis and septic shock. J Intensive Care Med. 2007;22:363-73.

626. Ueda S, Nishio K, Akai Y, Fukushima H, Ueyama T, Kawai Y et al. Prognostic value of increased plasma levels of brain natriuretic peptide in patients with septic shock. Shock. 2006;26:134-9.

627. Varpula M, Pulkki K, Karlsson S, Ruokonen E, Pettila V. Predictive value of N-terminal pro-brain natriuretic peptide in severe sepsis and septic shock. Crit Care Med. 2007;35:1277-83.

628. Wang H, Li Z, Yin M, Chen XM, Ding SF, Li C et al. Combination of acute physiology and chronic health evaluation II score, early lactate area, and N-terminal prohormone of brain natriuretic peptide levels as a predictor of mortality in geriatric patients with septic shock. J Crit Care. 2015;30:304-9.

629. Zhang XH, Dong Y, Chen YD, Zhou P, Wang JD, Wen FQ. Serum N-terminal pro-brain natriuretic peptide level is a significant prognostic factor in patients with severe sepsis among Southwest Chinese population. Eur Rev Med Pharmacol Sci. 2013;17:517-21.

630. Casey L, Krieger B, Kohler J, Rice C, Oparil S, Szidon P. Decreased serum angiotensin converting enzyme in adult respiratory distress syndrome associated with sepsis: a preliminary report. Crit Care Med. 1981;9:651-4.

631. Deitz DM, Swartz KR, Wright M, Murphy E, Connell RS, Harrison MW. Effects of E. coli endotoxin on rat plasma angiotensin converting enzyme activity in vitro and in vivo. Circ Shock. 1987;21:23-9.

632. Meijvis SC, Cornips MC, Endeman H, Ruven HJ, Danser AH, Biesma DH et al. Prognostic value of serum angiotensin-converting enzyme activity for outcome of community-acquired pneumonia. Clin Chem Lab Med. 2011;49:1525-32.

633. Rice CL, Kohler JP, Casey L, Szidon JP, Daise M, Moss GS. Angiotensin-converting enzyme (ACE) in sepsis. Circ Shock. 1983;11:59-63.

634. Tsantes A, Tsangaris I, Kopterides P, Nikolopoulos G, Kalamara E, Antonakos G et al. Angiotensin converting enzyme (ACE) insertion/deletion (I/D) polymorphism and circulating ACE levels are not associated with outcome in critically ill septic patients. Clin Chem Lab Med. 2011;50:293-9.

635. Villar J, Flores C, Perez-Mendez L, Maca-Meyer N, Espinosa E, Blanco J et al. Angiotensin-converting enzyme insertion/deletion polymorphism is not associated with susceptibility and outcome in sepsis and acute respiratory distress syndrome. Intensive Care Med. 2008;34:488-95.

636. Walther S, Berg S, Jansson I, Lennquist S. Activity of serum angiotensin converting enzyme in septic pigs treated with intrapulmonary corticosteroid. Eur J Surg. 1994;160:3-7.

637. Brauner JS, Rohde LE, Clausell N. Circulating endothelin-1 and tumor necrosis factor-alpha: early predictors of mortality in patients with septic shock. Intensive Care Med. 2000;26:305-13.

638. Kaffarnik MF, Ahmadi N, Lock JF, Wuensch T, Pratschke J, Stockmann M et al. Correlation between plasma endothelin-1 levels and severity of septic liver failure quantified by maximal liver function capacity (LiMAx test). A prospective study. PLoS One. 2017;12:e0178237.

639. Nakamura T, Kasai K, Sekiguchi Y, Banba N, Takahashi K, Emoto T et al. Elevation of plasma endothelin concentrations during endotoxin shock in dogs. Eur J Pharmacol. 1991;205:277-82.

640. Piechota M, Banach M, Irzmanski R, Barylski M, Piechota-Urbanska M, Kowalski J et al. Plasma endothelin-1 levels in septic patients. J Intensive Care Med. 2007;22:232-9.

641. Schuetz P, Christ-Crain M, Morgenthaler NG, Struck J, Bergmann A, Muller B. Circulating precursor levels of endothelin-1 and adrenomedullin, two endothelium-derived, counteracting substances, in sepsis. Endothelium. 2007;14:345-51.

642. Sakai S, Miyauchi T, Sakurai T, Kasuya Y, Ihara M, Yamaguchi I et al. Endogenous endothelin-1 participates in the maintenance of cardiac function in rats with congestive heart failure. Marked increase in endothelin-1 production in the failing heart. Circulation. 1996;93:1214-22.

643. Ware LB, Koyama T, Zhao Z, Janz DR, Wickersham N, Bernard GR et al. Biomarkers of lung epithelial injury and inflammation distinguish severe sepsis patients with acute respiratory distress syndrome. Crit Care. 2013;17:R253.

644. Broeckaert F, Bernard A. Clara cell secretory protein (CC16): characteristics and perspectives as lung peripheral biomarker. Clin Exp Allergy. 2000;30:469-75.

645. Hsu CH, Reyes LF, Orihuela CJ, Buitrago R, Anzueto A, Soni NJ et al. Chromogranin A levels and mortality in patients with severe sepsis. Biomarkers. 2015;20:171-6.

646. Rosjo H, Nygard S, Kaukonen KM, Karlsson S, Stridsberg M, Ruokonen E et al. Prognostic value of chromogranin A in severe sepsis: data from the FINNSEPSIS study. Intensive Care Med. 2012;38:820-9.

647. Zhang D, Lavaux T, Voegeli AC, Lavigne T, Castelain V, Meyer N et al. Prognostic value of chromogranin A at admission in critically ill patients: a cohort study in a medical intensive care unit. Clin Chem. 2008;54:1497-503.

648. Hsu AA, Fenton K, Weinstein S, Carpenter J, Dalton H, Bell MJ. Neurological injury markers in children with septic shock. Pediatr Crit Care Med. 2008;9:245-51.

649. Hol EM, Pekny M. Glial fibrillary acidic protein (GFAP) and the astrocyte intermediate filament system in diseases of the central nervous system. Curr Opin Cell Biol. 2015;32:121-30.

650. Anderson BJ, Reilly JP, Shashaty MGS, Palakshappa JA, Wysoczanski A, Dunn TG et al. Admission plasma levels of the neuronal injury marker neuron-specific enolase are associated with mortality and delirium in sepsis. J Crit Care. 2016;36:18-23.

651. Nguyen DN, Spapen H, Su F, Schiettecatte J, Shi L, Hachimi-Idrissi S et al. Elevated serum levels of S-100beta protein and neuron-specific enolase are associated with brain injury in patients with severe sepsis and septic shock. Crit Care Med. 2006;34:1967-74.

652. Yao B, Zhang LN, Ai YH, Liu ZY, Huang L. Serum S100beta is a better biomarker than neuron-specific enolase for sepsis-associated encephalopathy and determining its prognosis: a prospective and observational study. Neurochem Res. 2014;39:1263-9.

653. Fogel W, Krieger D, Veith M, Adams HP, Hund E, Storch-Hagenlocher B et al. Serum neuron-specific enolase as early predictor of outcome after cardiac arrest. Crit Care Med. 1997;25:1133-8.

654. Ehler J, Petzold A, Wittstock M, Kolbaske S, Gloger M, Henschel J et al. The prognostic value of neurofilament levels in patients with sepsis-associated encephalopathy - A prospective, pilot observational study. PLoS One. 2019;14:e0211184.

655. Skillback T, Farahmand B, Bartlett JW, Rosen C, Mattsson N, Nagga K et al. CSF neurofilament light differs in neurodegenerative diseases and predicts severity and survival. Neurology. 2014;83:1945-53.

656. Endo S, Sato N, Nakae H, Yamada Y, Makabe H, Abe H et al. Surfactant protein A and D (SP-A, AP-D) levels in patients with septic ARDS. Res Commun Mol Pathol Pharmacol. 2002;111:245-51.

657. Lewis JF, Veldhuizen R, Possmayer F, Sibbald W, Whitsett J, Qanbar R et al. Altered alveolar surfactant is an early marker of acute lung injury in septic adult sheep. Am J Respir Crit Care Med. 1994;150:123-30.

658. Kuroki Y, Takahashi H, Chiba H, Akino T. Surfactant proteins A and D: disease markers. Biochim Biophys Acta. 1998;1408:334-45.

659. Liu XW, Ma T, Cai Q, Wang L, Song HW, Liu Z. Elevation of serum PARK7 and IL-8 levels is associated with acute lung injury in patients with severe sepsis/septic shock. J Intensive Care Med. 2019;34:662-8.

660. Wilson MA. The role of cysteine oxidation in DJ-1 function and dysfunction. Antioxid Redox Signal. 2011;15:111-22.

661. Cheng H, Fan WZ, Wang SC, Liu ZH, Zang HL, Wang LZ et al. N-terminal pro-brain natriuretic peptide and cardiac troponin I for the prognostic utility in elderly patients with severe sepsis or septic shock in intensive care unit: A retrospective study. J Crit Care. 2015;30:654-14.

662. Choon-ngarm T, Partpisanu P. Serum cardiac troponin-T as a prognostic marker in septic shock. J Med Assoc Thai. 2008;91:1818-21.

663. de Groot B, Verdoorn RC, Lameijer J, van der Velden J. High-sensitivity cardiac troponin T is an independent predictor of inhospital mortality in emergency department patients with suspected infection: a prospective observational derivation study. Emerg Med J. 2014;31:882-8.

664. Fenton KE, Sable CA, Bell MJ, Patel KM, Berger JT. Increases in serum levels of troponin I are associated with cardiac dysfunction and disease severity in pediatric patients with septic shock. Pediatr Crit Care Med. 2004;5:533-8.

665. Jeong HS, Lee TH, Bang CH, Kim JH, Hong SJ. Risk factors and outcomes of sepsis-induced myocardial dysfunction and stress-induced cardiomyopathy in sepsis or septic shock: A comparative retrospective study. Medicine (Baltimore). 2018;97:e0263.

666. John J, Woodward DB, Wang Y, Yan SB, Fisher D, Kinasewitz GT et al. Troponin-I as a prognosticator of mortality in severe sepsis patients. J Crit Care. 2010;25:270-5.

667. Kang EW, Na HJ, Hong SM, Shin SK, Kang SW, Choi KH et al. Prognostic value of elevated cardiac troponin I in ESRD patients with sepsis. Nephrol Dial Transplant. 2009;24:1568-73.

668. Landesberg G, Jaffe AS, Gilon D, Levin PD, Goodman S, Abu-Baih A et al. Troponin elevation in severe sepsis and septic shock: the role of left ventricular diastolic dysfunction and right ventricular dilatation*. Crit Care Med. 2014;42:790-800.

669. Masson S, Caironi P, Fanizza C, Carrer S, Caricato A, Fassini P et al. Sequential N-terminal pro-B-type natriuretic peptide and high-sensitivity cardiac troponin measurements during albumin replacement in patients with severe sepsis or septic shock. Crit Care Med. 2016;44:707-16.

670. Mehta NJ, Khan IA, Gupta V, Jani K, Gowda RM, Smith PR. Cardiac troponin I predicts myocardial dysfunction and adverse outcome in septic shock. Int J Cardiol. 2004;95:13-7.

671. Oliveira NS, Silva VR, Castelo JS, Elias-Neto J, Pereira FE, Carvalho WB. Serum level of cardiac troponin I in pediatric patients with sepsis or septic shock. Pediatr Crit Care Med. 2008;9:414-7.

672. Vallabhajosyula S, Sakhuja A, Geske JB, Kumar M, Poterucha JT, Kashyap R et al. Role of admission troponin-T and serial troponin-T testing in predicting outcomes in severe sepsis and septic shock. J Am Heart Assoc. 2017;6.

673. Babuin L, Jaffe AS. Troponin: the biomarker of choice for the detection of cardiac injury. CMAJ. 2005;173:1191-202.

674. Dai X, Zeng Z, Fu C, Zhang S, Cai Y, Chen Z. Diagnostic value of neutrophil gelatinase-associated lipocalin, cystatin C, and soluble triggering receptor expressed on myeloid cells-1 in critically ill patients with sepsis-associated acute kidney injury. Crit Care. 2015;19:223.

675. Hong DY, Kim JW, Paik JH, Jung HM, Baek KJ, Park SO et al. Value of plasma neutrophil gelatinase-associated lipocalin in predicting the mortality of patients with sepsis at the emergency department. Clin Chim Acta. 2016;452:177-81.

676. Martensson J, Bell M, Oldner A, Xu S, Venge P, Martling CR. Neutrophil gelatinase-associated lipocalin in adult septic patients with and without acute kidney injury. Intensive Care Med. 2010;36:1333-40.

677. Martensson J, Bell M, Xu S, Bottai M, Ravn B, Venge P et al. Association of plasma neutrophil gelatinase-associated lipocalin (NGAL) with sepsis and acute kidney dysfunction. Biomarkers. 2013;18:349-56.

678. Pynn JM, Parravicini E, Saiman L, Bateman DA, Barasch JM, Lorenz JM. Urinary neutrophil gelatinase-associated lipocalin: potential biomarker for late-onset sepsis. Pediatr Res. 2015;78:76-81.

679. Shao Y, Fan Y, Xie Y, Yin L, Zhang Y, Deng L et al. Effect of continuous renal replacement therapy on kidney injury molecule-1 and neutrophil gelatinase-associated lipocalin in patients with septic acute kidney injury. Exp Ther Med. 2017;13:3594-602.

680. Shapiro NI, Trzeciak S, Hollander JE, Birkhahn R, Otero R, Osborn TM et al. The diagnostic accuracy of plasma neutrophil gelatinase-associated lipocalin in the prediction of acute kidney injury in emergency department patients with suspected sepsis. Ann Emerg Med. 2010;56:52-9.

681. Srisawat N, Murugan R, Lee M, Kong L, Carter M, Angus DC et al. Plasma neutrophil gelatinase-associated lipocalin predicts recovery from acute kidney injury following community-acquired pneumonia. Kidney Int. 2011;80:545-52.

682. Wang B, Chen G, Li J, Zeng Y, Wu Y, Yan X. Neutrophil gelatinase-associated lipocalin predicts myocardial dysfunction and mortality in severe sepsis and septic shock. Int J Cardiol. 2017;227:589-94.

683. Wang B, Chen G, Zhang J, Xue J, Cao Y, Wu Y. Increased neutrophil gelatinase-associated lipocalin is associated with mortality and multiple organ dysfunction syndrome in severe sepsis and septic shock. Shock. 2015;44:234-8.

684. Wang H, Zhang M, Mao H, Cheng Z, Zhang Q, Jiang C et al. Serum neutrophil gelatinase-associated lipocalin and proinflammatory cytokines in pigs with septic versus non-septic acute kidney injury. Int Urol Nephrol. 2015;47:413-20.

685. Wang M, Zhang Q, Zhao X, Dong G, Li C. Diagnostic and prognostic value of neutrophil gelatinase-associated lipocalin, matrix metalloproteinase-9, and tissue inhibitor of matrix metalloproteinases-1 for sepsis in the Emergency Department: an observational study. Crit Care. 2014;18:634.

686. Park HS, Kim JW, Lee KR, Hong DY, Park SO, Kim SY et al. Urinary neutrophil gelatinase-associated lipocalin as a biomarker of acute kidney injury in sepsis patients in the emergency department. Clin Chim Acta. 2019;495:552-5.

687. Mishra J, Ma Q, Prada A, Mitsnefes M, Zahedi K, Yang J et al. Identification of neutrophil gelatinase-associated lipocalin as a novel early urinary biomarker for ischemic renal injury. J Am Soc Nephrol. 2003;14:2534-43.

688. Chen YX, Li CS. The prognostic and risk-stratified value of heart-type fatty acid-binding protein in septic patients in the emergency department. J Crit Care. 2014;29:512-6.

689. Jo YH, Kim K, Lee JH, Rhee JE, Lee JH, Kang KW et al. Heart-type fatty acid-binding protein as a prognostic factor in patients with severe sepsis and septic shock. Am J Emerg Med. 2012;30:1749-55.

690. Zhang ZC, Dai HW, Yu YH, Yang JD, Hu CB. Usefulness of heart-type fatty acid-binding protein in patients with severe sepsis. J Crit Care. 2012;27:415-8.

691. Fournier NC, Richard MA. Role of fatty acid-binding protein in cardiac fatty acid oxidation. Mol Cell Biochem. 1990;98:149-59.

692. Koo DJ, Zhou M, Chaudry IH, Wang P. Plasma alpha-glutathione S-transferase: a sensitive indicator of hepatocellular damage during polymicrobial sepsis. Arch Surg. 2000;135:198-203.

693. Walshe CM, Odejayi F, Ng S, Marsh B. Urinary glutathione S-transferase as an early marker for renal dysfunction in patients admitted to intensive care with sepsis. Crit Care Resusc. 2009;11:204-9.

694. Harrison DJ, Kharbanda R, Cunningham DS, McLellan LI, Hayes JD. Distribution of glutathione S-transferase isoenzymes in human kidney: basis for possible markers of renal injury. J Clin Pathol. 1989;42:624-8.

695. Rosjo H, Masson S, Caironi P, Stridsberg M, Magnoli M, Christensen G et al. Prognostic value of secretoneurin in patients with severe sepsis and septic shock: Data from the Albumin Italian Outcome Sepsis Study. Crit Care Med. 2018;46:e404-e410.

696. Rosjo H, Stridsberg M, Ottesen AH, Nygard S, Christensen G, Pettila V et al. Prognostic value of secretoneurin in critically ill patients with infections. Crit Care Med. 2016;44:1882-90.

697. Ottesen AH, Louch WE, Carlson CR, Landsverk OJB, Kurola J, Johansen RF et al. Secretoneurin is a novel prognostic cardiovascular biomarker associated with cardiomyocyte calcium handling. J Am Coll Cardiol. 2015;65:339-51.

698. Igonin AA, Armstrong VW, Shipkova M, Kukes VG, Oellerich M. The monoethylglycinexylidide (MEGX) test as a marker of hepatic dysfunction in septic patients with pneumonia. Clin Chem Lab Med. 2000;38:1125-8.

699. McKindley DS, Chichester C, Raymond R. Effect of endotoxin shock on the clearance of lidocaine and indocyanine green in the perfused rat liver. Shock. 1999;12:468-72.

700. Bargetzi MJ, Aoyama T, Gonzalez FJ, Meyer UA. Lidocaine metabolism in human liver microsomes by cytochrome P450IIIA4. Clin Pharmacol Ther. 1989;46:521-7.

701. Rahmel T, Schafer ST, Frey UH, Adamzik M, Peters J. Increased circulating microRNA-122 is a biomarker for discrimination and risk stratification in patients defined by sepsis-3 criteria. PLoS One. 2018;13:e0197637.

702. Wang HJ, Deng J, Wang JY, Zhang PJ, Xin Z, Xiao K et al. Serum miR-122 levels are related to coagulation disorders in sepsis patients. Clin Chem Lab Med. 2014;52:927-33.

703. Zhang W, Jia J, Liu Z, Si D, Ma L, Zhang G. Circulating microRNAs as biomarkers for Sepsis secondary to pneumonia diagnosed via Sepsis 3.0. BMC Pulm Med. 2019;19:93.

704. Jopling C. Liver-specific microRNA-122: Biogenesis and function. RNA Biol. 2012;9:137-42.

705. Xu Y, Shao B. Circulating long noncoding RNA ZNFX1 antisense RNA negatively correlates with disease risk, severity, inflammatory markers, and predicts poor prognosis in sepsis patients. Medicine (Baltimore). 2019;98:e14558.

706. Gui F, Peng H, Liu Y. Elevated circulating lnc-ANRIL/miR-125a axis level predicts higher risk, more severe disease condition, and worse prognosis of sepsis. J Clin Lab Anal. 2019;33:e22917.

707. Yu Z, Rayile A, Zhang X, Li Y, Zhao Q. Ulinastatin protects against lipopolysaccharide-induced cardiac microvascular endothelial cell dysfunction via downregulation of lncRNA MALAT1 and EZH2 in sepsis. Int J Mol Med. 2017;39:1269-76.

708. Larsson A, Lipcsey M, Sjolin J, Hansson LO, Eriksson MB. Slight increase of serum S-100B during porcine endotoxemic shock may indicate blood-brain barrier damage. Anesth Analg. 2005;101:1465-9.

709. Piazza O, Russo E, Cotena S, Esposito G, Tufano R. Elevated S100B levels do not correlate with the severity of encephalopathy during sepsis. Br J Anaesth. 2007;99:518-21.

710. Erikson K, Ala-Kokko TI, Koskenkari J, Liisanantti JH, Kamakura R, Herzig KH et al. Elevated serum S-100beta in patients with septic shock is associated with delirium. Acta Anaesthesiol Scand. 2019;63:69-73.

711. Blyth BJ, Farhavar A, Gee C, Hawthorn B, He H, Nayak A et al. Validation of serum markers for blood-brain barrier disruption in traumatic brain injury. J Neurotrauma. 2009;26:1497-507.

712. Drey M, Behnes M, Kob R, Lepiorz D, Hettwer S, Bollheimer C et al. C-terminal agrin fragment (CAF) reflects renal function in patients suffering from severe sepsis or septic shock. Clin Lab. 2015;61:69-76.

713. Devetzis V, Daryadel A, Roumeliotis S, Theodoridis M, Wagner CA, Hettwer S et al. C-terminal fragment of agrin (CAF): A novel marker for progression of kidney disease in type 2 diabetics. PLoS One. 2015;10:e0143524.

714. Kim H, Hur M, Lee S, Marino R, Magrini L, Cardelli P et al. Proenkephalin, neutrophil gelatinase-associated lipocalin, and estimated glomerular filtration rates in patients with sepsis. Ann Lab Med. 2017;37:388-97.

715. Marino R, Struck J, Hartmann O, Maisel AS, Rehfeldt M, Magrini L et al. Diagnostic and short-term prognostic utility of plasma pro-enkephalin (pro-ENK) for acute kidney injury in patients admitted with sepsis in the emergency department. J Nephrol. 2015;28:717-24.

716. Denning GM, Ackermann LW, Barna TJ, Armstrong JG, Stoll LL, Weintraub NL et al. Proenkephalin expression and enkephalin release are widely observed in non-neuronal tissues. Peptides. 2008;29:83-92.

717. Schaalan M, Mohamed W. Predictive ability of circulating osteoprotegerin as a novel biomarker for early detection of acute kidney injury induced by sepsis. Eur Cytokine Netw. 2017;28:52-62.

718. Tu Y, Wang H, Sun R, Ni Y, Ma L, Xv F et al. Urinary netrin-1 and KIM-1 as early biomarkers for septic acute kidney injury. Ren Fail. 2014;36:1559-63.

719. Ichimura T, Asseldonk EJ, Humphreys BD, Gunaratnam L, Duffield JS, Bonventre JV. Kidney injury molecule-1 is a phosphatidylserine receptor that confers a phagocytic phenotype on epithelial cells. J Clin Invest. 2008;118:1657-68.

720. Poeze M, Luiking YC, Breedveld P, Manders S, Deutz NE. Decreased plasma glutamate in early phases of septic shock with acute liver dysfunction is an independent predictor of survival. Clin Nutr. 2008;27:523-30.

721. Kantrow SP, Taylor DE, Carraway MS, Piantadosi CA. Oxidative metabolism in rat hepatocytes and mitochondria during sepsis. Arch Biochem Biophys. 1997;345:278-88.

722. Mussap M, Noto A, Fravega M, Fanos V. Urine neutrophil gelatinase-associated lipocalin (uNGAL) and netrin-1: are they effectively improving the clinical management of sepsis-induced acute kidney injury (AKI)? J Matern Fetal Neonatal Med. 2011;24 Suppl 2:15-7.

723. Reeves WB, Kwon O, Ramesh G. Netrin-1 and kidney injury. II. Netrin-1 is an early biomarker of acute kidney injury. Am J Physiol Renal Physiol. 2008;294:F731-F738.

724. Jitpean S, Pettersson A, Hoglund OV, Holst BS, Olsson U, Hagman R. Increased concentrations of Serum amyloid A in dogs with sepsis caused by pyometra. BMC Vet Res. 2014;10:273.

725. Arnon S, Litmanovitz I, Regev RH, Bauer S, Shainkin-Kestenbaum R, Dolfin T. Serum amyloid A: an early and accurate marker of neonatal early-onset sepsis. J Perinatol. 2007;27:297-302.

726. Arnon S, Litmanovitz I, Regev R, Bauer S, Lis M, Shainkin-Kestenbaum R et al. Serum amyloid A protein is a useful inflammatory marker during late-onset sepsis in preterm infants. Biol Neonate. 2005;87:105-10.

727. Cetinkaya M, Ozkan H, Koksal N, Celebi S, Hacimustafaoglu M. Comparison of serum amyloid A concentrations with those of C-reactive protein and procalcitonin in diagnosis and follow-up of neonatal sepsis in premature infants. J Perinatol. 2009;29:225-31.

728. Cicarelli DD, Vieira JE, Bensenor FE. Comparison of C-reactive protein and serum amyloid a protein in septic shock patients. Mediators Inflamm. 2008;2008:631414.

729. Huttunen T, Teppo AM, Lupisan S, Ruutu P, Nohynek H. Correlation between the severity of infectious diseases in children and the ratio of serum amyloid A protein and C-reactive protein. Scand J Infect Dis. 2003;35:488-90.

730. Lannergard A, Larsson A, Kragsbjerg P, Friman G. Correlations between serum amyloid A protein and C-reactive protein in infectious diseases. Scand J Clin Lab Invest. 2003;63:267-72.

731. Lannergard A, Viberg A, Cars O, Karlsson MO, Sandstrom M, Larsson A. The time course of body temperature, serum amyloid A protein, C-reactive protein and interleukin-6 in patients with bacterial infection during the initial 3 days of antibiotic therapy. Scand J Infect Dis. 2009;41:663-71.

732. Orro T, Sankari S, Pudas T, Oksanen A, Soveri T. Acute phase response in reindeer after challenge with Escherichia coli endotoxin. Comp Immunol Microbiol Infect Dis. 2004;27:413-22.

733. Ucar B, Yildiz B, Aksit MA, Yarar C, Colak O, Akbay Y et al. Serum amyloid A, procalcitonin, tumor necrosis factor-alpha, and interleukin-1beta levels in neonatal late-onset sepsis. Mediators Inflamm. 2008;2008:737141.

734. Yu MH, Chen MH, Han F, Li Q, Sun RH, Tu YX. Prognostic value of the biomarkers serum amyloid A and nitric oxide in patients with sepsis. Int Immunopharmacol. 2018;62:287-92.

735. Uhlar CM, Whitehead AS. Serum amyloid A, the major vertebrate acute-phase reactant. Eur J Biochem. 1999;265:501-23.

736. Ye RD, Sun L. Emerging functions of serum amyloid A in inflammation. J Leukoc Biol. 2015;98:923-9.

737. Endo S, Inada K, Nakae H, Takakuwa T, Yamada Y, Suzuki T et al. Plasma levels of type II phospholipase A2 and cytokines in patients with sepsis. Res Commun Mol Pathol Pharmacol. 1995;90:413-21.

738. Huang Z, Jiang H, Cui X, Liang G, Chen Y, Wang T et al. Elevated serum levels of lipoproteinassociated phospholipase A2 predict mortality rates in patients with sepsis. Mol Med Rep. 2018;17:1791-8.

739. Rintala E, Pulkki K, Mertsola J, Nevalainen T, Nikoskelainen J. Endotoxin, interleukin-6 and phospholipase-A2 as markers of sepsis in patients with hematological malignancies. Scand J Infect Dis. 1995;27:39-43.

740. Tan TL, Goh YY. The role of group IIA secretory phospholipase A2 (sPLA2-IIA) as a biomarker for the diagnosis of sepsis and bacterial infection in adults-A systematic review. PLoS One. 2017;12:e0180554.

741. Uusitalo-Seppala R, Peuravuori H, Koskinen P, Vahlberg T, Rintala EM. Role of plasma bactericidal/permeability-increasing protein, group IIA phospholipase A(2), C-reactive protein, and white blood cell count in the early detection of severe sepsis in the emergency department. Scand J Infect Dis. 2012;44:697-704.

742. Yokota Y, Ikeda M, Higashino K, Nakano K, Fujii N, Arita H et al. Enhanced tissue expression and elevated circulating level of phospholipase A(2) receptor during murine endotoxic shock. Arch Biochem Biophys. 2000;379:7-17.

743. Weinrauch Y, Abad C, Liang NS, Lowry SF, Weiss J. Mobilization of potent plasma bactericidal activity during systemic bacterial challenge. Role of group IIA phospholipase A2. J Clin Invest. 1998;102:633-8.

744. Ayar G, Atmaca YM, Alisik M, Erel O. Effects of paraoxonase, arylesterase, ceruloplasmin, catalase, and myeloperoxidase activities on prognosis in pediatric patients with sepsis. Clin Biochem. 2017;50:414-7.

745. Suri M, Sharma VK, Thirupuram S. Evaluation of ceruloplasmin in neonatal septicemia. Indian Pediatr. 1991;28:489-93.

746. Chiarla C, Giovannini I, Siegel JH. Patterns of correlation of plasma ceruloplasmin in sepsis. J Surg Res. 2008;144:107-10.

747. Gradel KO, Thomsen RW, Lundbye-Christensen S, Nielsen H, Schonheyder HC. Baseline C-reactive protein level as a predictor of mortality in bacteraemia patients: a population-based cohort study. Clin Microbiol Infect. 2011;17:627-32.

748. Rabello LSCF, Povoa P, Lapa E Silva JR, Azevedo LCP, da Silva Ramos FJ, Lisboa T et al. Patterns of C-reactive protein ratio predicts outcomes in healthcare-associated pneumonia in critically ill patients with cancer. J Crit Care. 2017;42:231-7.

749. Stoma I, Karpov I, Uss A, Rummo O, Milanovich N, Iskrov I. Diagnostic value of sepsis biomarkers in hematopoietic stem cell transplant recipients in a condition of high prevalence of gram-negative pathogens. Hematol Oncol Stem Cell Ther. 2017;10:15-21.

750. Brenner T, Uhle F, Fleming T, Wieland M, Schmoch T, Schmitt F et al. Soluble TREM-1 as a diagnostic and prognostic biomarker in patients with septic shock: an observational clinical study. Biomarkers. 2017;22:63-9.

751. Quinten VM, van Meurs M, Ter Maaten JC, Ligtenberg JJ. Trends in vital signs and routine biomarkers in patients with sepsis during resuscitation in the emergency department: a prospective observational pilot study. BMJ Open. 2016;6:e009718.

752. Ryu JA, Yang JH, Lee D, Park CM, Suh GY, Jeon K et al. Clinical usefulness of procalcitonin and C-reactive protein as outcome predictors in critically ill patients with severe sepsis and septic shock. PLoS One. 2015;10:e0138150.

753. Pereira JM, Teixeira-Pinto A, Basilio C, Sousa-Dias C, Mergulhao P, Paiva JA. Can we predict pneumococcal bacteremia in patients with severe community-acquired pneumonia? J Crit Care. 2013;28:970-4.

754. Oliveira CF, Botoni FA, Oliveira CR, Silva CB, Pereira HA, Serufo JC et al. Procalcitonin versus C-reactive protein for guiding antibiotic therapy in sepsis: a randomized trial. Crit Care Med. 2013;41:2336-43.

755. Cetinkaya M, Ozkan H, Koksal N, Akaci O, Ozgur T. Comparison of the efficacy of serum amyloid A, C-reactive protein, and procalcitonin in the diagnosis and follow-up of necrotizing enterocolitis in premature infants. J Pediatr Surg. 2011;46:1482-9.

756. Iapichino G, Marzorati S, Umbrello M, Baccalini R, Barassi A, Cainarca M et al. Daily monitoring of biomarkers of sepsis in complicated long-term ICU-patients: can it support treatment decisions? Minerva Anestesiol. 2010;76:814-23.

757. Ruiz-Alvarez MJ, Garcia-Valdecasas S, De Pablo R, Sanchez Garcia M, Coca C, Groeneveld TW et al. Diagnostic efficacy and prognostic value of serum procalcitonin concentration in patients with suspected sepsis. J Intensive Care Med. 2009;24:63-71.

758. Castelli GP, Pognani C, Cita M, Stuani A, Sgarbi L, Paladini R. Procalcitonin, C-reactive protein, white blood cells and SOFA score in ICU: diagnosis and monitoring of sepsis. Minerva Anestesiol. 2006;72:69-80.

759. Laborada G, Rego M, Jain A, Guliano M, Stavola J, Ballabh P et al. Diagnostic value of cytokines and C-reactive protein in the first 24 hours of neonatal sepsis. Am J Perinatol. 2003;20:491-501.

760. Ng PC, Cheng SH, Chui KM, Fok TF, Wong MY, Wong W et al. Diagnosis of late onset neonatal sepsis with cytokines, adhesion molecule, and C-reactive protein in preterm very low birthweight infants. Arch Dis Child Fetal Neonatal Ed. 1997;77:F221-F227.

761. Ratzinger F, Haslacher H, Perkmann T, Schmetterer KG, Poeppl W, Mitteregger D et al. Sepsis biomarkers in neutropaenic systemic inflammatory response syndrome patients on standard care wards. Eur J Clin Invest. 2015;45:815-23.

762. Behnes M, Bertsch T, Lepiorz D, Lang S, Trinkmann F, Brueckmann M et al. Diagnostic and prognostic utility of soluble CD 14 subtype (presepsin) for severe sepsis and septic shock during the first week of intensive care treatment. Crit Care. 2014;18:507.

763. Julian-Jimenez A, Timon ZJ, Laserna Mendieta EJ, Parejo MR, Flores CM, Gallardo SP. [Ability of procalcitonin to predict bacteremia in patients with community acquired pneumonia]. Med Clin (Barc). 2014;142:285-92.

764. Assarsson J, Korner U, Lundholm K. Evaluation of procalcitonin as a marker to predict antibiotic response in adult patients with acute appendicitis: a prospective observational study. Surg Infect (Larchmt). 2014;15:601-5.

765. Somech R, Zakuth V, Assia A, Jurgenson U, Spirer Z. Procalcitonin correlates with C-reactive protein as an acute-phase reactant in pediatric patients. Isr Med Assoc J. 2000;2:147-50.

766. McGrane S, Girard TD, Thompson JL, Shintani AK, Woodworth A, Ely EW et al. Procalcitonin and C-reactive protein levels at admission as predictors of duration of acute brain dysfunction in critically ill patients. Crit Care. 2011;15:R78.

767. Jordan I, Corniero P, Balaguer M, Ortiz J, Vila D, Velasco J et al. Adrenomedullin is a useful biomarker for the prognosis of critically ill septic children. Biomark Med. 2014;8:1065-72.

768. Bolia R, Srivastava A, Marak R, Yachha SK, Poddar U. Role of procalcitonin and C-reactive protein as biomarkers of infection in children with liver disease. J Pediatr Gastroenterol Nutr. 2016;63:406-11.

769. Oberhoffer M, Vogelsang H, Russwurm S, Hartung T, Reinhart K. Outcome prediction by traditional and new markers of inflammation in patients with sepsis. Clin Chem Lab Med. 1999;37:363-8.

770. Kocazeybek B, Kucukoglu S, Oner YA. Procalcitonin and C-reactive protein in infective endocarditis: correlation with etiology and prognosis. Chemotherapy. 2003;49:76-84.

771. Reith HB, Mittelkotter U, Debus ES, Kussner C, Thiede A. Procalcitonin in early detection of postoperative complications. Dig Surg. 1998;15:260-5.

772. Lamping F, Jack T, Rubsamen N, Sasse M, Beerbaum P, Mikolajczyk RT et al. Development and validation of a diagnostic model for early differentiation of sepsis and non-infectious SIRS in critically ill children - a data-driven approach using machine-learning algorithms. BMC Pediatr. 2018;18:112.

773. Davido B, Makhloufi S, Matt M, Calin R, Senard O, Perronne C et al. Changes in eosinophil count during bacterial infection: revisiting an old marker to assess the efficacy of antimicrobial therapy. Int J Infect Dis. 2017;61:62-6.

774. Rios-Toro JJ, Marquez-Coello M, Garcia-Alvarez JM, Martin-Aspas A, Rivera-Fernandez R, Saez de BA et al. Soluble membrane receptors, interleukin 6, procalcitonin and C reactive protein as prognostic markers in patients with severe sepsis and septic shock. PLoS One. 2017;12:e0175254.

775. Suberviola B, Castellanos-Ortega A, Gonzalez-Castro A, Garcia-Astudillo LA, Fernandez-Miret B. [Prognostic value of procalcitonin, C-reactive protein and leukocytes in septic shock]. Med Intensiva. 2012;36:177-84.

776. Szederjesi J, Almasy E, Lazar A, Hutanu A, Badea I, Georgescu A. An evaluation of serum procalcitonin and C-reactive protein levels as diagnostic and prognostic biomarkers of severe sepsis. J Crit Care Med (Targu Mures). 2015;1:147-53.

777. Lelubre C, Anselin S, Zouaoui Boudjeltia K, Biston P, Piagnerelli M. Interpretation of C-reactive protein concentrations in critically ill patients. Biomed Res Int. 2013;2013:124021.

778. Garcia PC, Longhi F, Branco RG, Piva JP, Lacks D, Tasker RC. Ferritin levels in children with severe sepsis and septic shock. Acta Paediatr. 2007;96:1829-31.

779. Branco RG, Garcia PC. Ferritin and C-reactive protein as markers of systemic inflammation in sepsis. Pediatr Crit Care Med. 2017;18:194-6.

780. Gornik O, Gornik I, Kolednjak IZ, Lauc G. Change of transferrin sialylation differs between mild sepsis and severe sepsis and septic shock. Intern Med. 2011;50:861-9.

781. Piagnerelli M, Boudjeltia KZ, Nuyens V, De Backer D, Su F, Wang Z et al. Rapid alterations in transferrin sialylation during sepsis. Shock. 2005;24:48-52.

782. Barroso-Sousa R, Lobo RR, Mendonca PR, Memoria RR, Spiller F, Cunha FQ et al. Decreased levels of alpha-1-acid glycoprotein are related to the mortality of septic patients in the emergency department. Clinics (Sao Paulo). 2013;68:1134-9.

783. Brinkman-van der Linden EC, van Ommen EC, van DW. Glycosylation of alpha 1-acid glycoprotein in septic shock: changes in degree of branching and in expression of sialyl Lewis(x) groups. Glycoconj J. 1996;13:27-31.

784. Scotte M, Daveau M, Hiron M, Delers F, Lemeland JF, Teniere P et al. Interleukin-6 (IL-6) and acute-phase proteins in rats with biliary sepsis. Eur Cytokine Netw. 1991;2:177-82.

785. Xiao K, Su L, Yan P, Han B, Li J, Wang H et al. alpha-1-Acid glycoprotein as a biomarker for the early diagnosis and monitoring the prognosis of sepsis. J Crit Care. 2015;30:744-51.

786. Hsiao SY, Lai YR, Kung CT, Tsai NW, Su CM, Huang CC et al. alpha-1-Acid glycoprotein concentration as an outcome predictor in adult patients with sepsis. Biomed Res Int. 2019;2019:3174896.

787. Hochepied T, Berger FG, Baumann H, Libert C. Alpha(1)-acid glycoprotein: an acute phase protein with inflammatory and immunomodulating properties. Cytokine Growth Factor Rev. 2003;14:25-34.

788. Li H, Rose MJ, Tran L, Zhang J, Miranda LP, James CA et al. Development of a method for the sensitive and quantitative determination of hepcidin in human serum using LC-MS/MS. J Pharmacol Toxicol Methods. 2009;59:171-80.

789. Wu TW, Tabangin M, Kusano R, Ma Y, Ridsdale R, Akinbi H. The utility of serum hepcidin as a biomarker for late-onset neonatal sepsis. J Pediatr. 2013;162:67-71.

790. Nemeth E, Valore EV, Territo M, Schiller G, Lichtenstein A, Ganz T. Hepcidin, a putative mediator of anemia of inflammation, is a type II acute-phase protein. Blood. 2003;101:2461-3.

791. Ng PC, Ang IL, Chiu RW, Li K, Lam HS, Wong RP et al. Host-response biomarkers for diagnosis of late-onset septicemia and necrotizing enterocolitis in preterm infants. J Clin Invest. 2010;120:2989-3000.

792. Khovidhunkit W, Kim MS, Memon RA, Shigenaga JK, Moser AH, Feingold KR et al. Effects of infection and inflammation on lipid and lipoprotein metabolism: mechanisms and consequences to the host. J Lipid Res. 2004;45:1169-96.

793. Suri M, Thirupuram S, Sharma VK. Diagnostic and prognostic utility of C-reactive protein, alpha-1-antitrypsin and alpha-2-macroglobulin in neonatal sepsis: a comparative account. Indian Pediatr. 1991;28:1159-64.

794. Andrejko KM, Chen J, Deutschman CS. Intrahepatic STAT-3 activation and acute phase gene expression predict outcome after CLP sepsis in the rat. Am J Physiol. 1998;275:G1423-G1429.

795. Kelly BJ, Lautenbach E, Nachamkin I, Coffin SE, Gerber JS, Fuchs BD et al. Combined biomarkers discriminate a low likelihood of bacterial infection among surgical intensive care unit patients with suspected sepsis. Diagn Microbiol Infect Dis. 2016;85:109-15.

796. Kelly BJ, Lautenbach E, Nachamkin I, Coffin SE, Gerber JS, Fuchs BD et al. Combined biomarkers predict acute mortality among critically ill patients with suspected sepsis. Crit Care Med. 2018;46:1106-13.

797. Borth W. Alpha 2-macroglobulin, a multifunctional binding protein with targeting characteristics. FASEB J. 1992;6:3345-53.

798. Fu P, Wang AM, He LY, Song JM, Xue JC, Wang CQ. Elevated serum ApoE levels are associated with bacterial infections in pediatric patients. J Microbiol Immunol Infect. 2014;47:122-9.

799. Wang C, Wang Y, Wang A, Fu P, Yang Y. The diagnostic value of apolipoprotein E in pediatric patients with invasive bacterial infections. Clin Biochem. 2012;45:215-8.

800. Li L, Thompson PA, Kitchens RL. Infection induces a positive acute phase apolipoprotein E response from a negative acute phase gene: role of hepatic LDL receptors. J Lipid Res. 2008;49:1782-93.

801. Berner R, Furll B, Stelter F, Drose J, Muller HP, Schutt C. Elevated levels of lipopolysaccharide-binding protein and soluble CD14 in plasma in neonatal early-onset sepsis. Clin Diagn Lab Immunol. 2002;9:440-5.

802. Garcia de Guadiana RL, Albaladejo Oton MD, Rebello Acebes S, Esteban TP, Hernando HA, Jimenez SE et al. Diagnostic accuracy of lipopolysaccharide-binding protein for sepsis in patients with suspected infection in the emergency department. Ann Clin Biochem. 2018;55:143-8.

803. Groselj-Grenc M, Ihan A, Pavcnik-Arnol M, Kopitar AN, Gmeiner-Stopar T, Derganc M. Neutrophil and monocyte CD64 indexes, lipopolysaccharide-binding protein, procalcitonin and C-reactive protein in sepsis of critically ill neonates and children. Intensive Care Med. 2009;35:1950-8.

804. Kitanovski L, Jazbec J, Hojker S, Derganc M. Diagnostic accuracy of lipopolysaccharide-binding protein for predicting bacteremia/clinical sepsis in children with febrile neutropenia: comparison with interleukin-6, procalcitonin, and C-reactive protein. Support Care Cancer. 2014;22:269-77.

805. Oude Nijhuis CSM, Vellenga E, Daenen SMGJ, van der Graaf WTA, Gietema JA, Groen HJM et al. Lipopolysaccharide-binding protein: a possible diagnostic marker for Gram-negative bacteremia in neutropenic cancer patients. Intensive Care Med. 2003;29:2157-61.

806. Ratzinger F, Schuardt M, Eichbichler K, Tsirkinidou I, Bauer M, Haslacher H et al. Utility of sepsis biomarkers and the infection probability score to discriminate sepsis and systemic inflammatory response syndrome in standard care patients. PLoS One. 2013;8:e82946.

807. ten Oever J, Tromp M, Bleeker-Rovers CP, Joosten LA, Netea MG, Pickkers P et al. Combination of biomarkers for the discrimination between bacterial and viral lower respiratory tract infections. J Infect. 2012;65:490-5.

808. Ubenauf KM, Krueger M, Henneke P, Berner R. Lipopolysaccharide binding protein is a potential marker for invasive bacterial infections in children. Pediatr Infect Dis J. 2007;26:159-62.

809. Villar J, Perez-Mendez L, Espinosa E, Flores C, Blanco J, Muriel A et al. Serum lipopolysaccharide binding protein levels predict severity of lung injury and mortality in patients with severe sepsis. PLoS One. 2009;4:e6818.

810. Sakr Y, Burgett U, Nacul FE, Reinhart K, Brunkhorst F. Lipopolysaccharide binding protein in a surgical intensive care unit: a marker of sepsis? Crit Care Med. 2008;36:2014-22.

811. Hattori T, Nishiyama H, Kato H, Ikegami S, Nagayama M, Asami S et al. Clinical value of procalcitonin for patients with suspected bloodstream infection. Am J Clin Pathol. 2014;141:43-51.

812. Shukeri WFWM, Ralib AM, Abdulah NZ, Mat-Nor MB. Sepsis mortality score for the prediction of mortality in septic patients. J Crit Care. 2018;43:163-8.

813. Ljungstrom L, Pernestig AK, Jacobsson G, Andersson R, Usener B, Tilevik D. Diagnostic accuracy of procalcitonin, neutrophil-lymphocyte count ratio, C-reactive protein, and lactate in patients with suspected bacterial sepsis. PLoS One. 2017;12:e0181704.

814. Wu RX, Chiu CC, Lin TC, Yang YS, Lee Y, Lin JC et al. Procalcitonin as a diagnostic biomarker for septic shock and bloodstream infection in burn patients from the Formosa Fun Coast dust explosion. J Microbiol Immunol Infect. 2017;50:872-8.

815. Heredia-Rodriguez M, Bustamante-Munguira J, Lorenzo M, Gomez-Sanchez E, Alvarez FJ, Fierro I et al. Procalcitonin and white blood cells, combined predictors of infection in cardiac surgery patients. J Surg Res. 2017;212:187-94.

816. Cabral L, Afreixo V, Santos F, Almeida L, Paiva JA. Procalcitonin for the early diagnosis of sepsis in burn patients: A retrospective study. Burns. 2017;43:1427-34.

817. Pan YP, Fang YP, Xu YH, Wang ZX, Shen JL. The diagnostic value of procalcitonin versus other biomarkers in prediction of bloodstream infection. Clin Lab. 2017;63:277-85.

818. Bloos F, Trips E, Nierhaus A, Briegel J, Heyland DK, Jaschinski U et al. Effect of sodium selenite administration and procalcitonin-guided therapy on mortality in patients with severe sepsis or septic shock: A randomized clinical trial. JAMA Intern Med. 2016;176:1266-76.

819. Trasy D, Tanczos K, Nemeth M, Hankovszky P, Lovas A, Mikor A et al. Early procalcitonin kinetics and appropriateness of empirical antimicrobial therapy in critically ill patients: A prospective observational study. J Crit Care. 2016;34:50-5.

820. Sharma P, Patel K, Baria K, Lakhia K, Malhotra A, Shah K et al. Procalcitonin level for prediction of postoperative infection in cardiac surgery. Asian Cardiovasc Thorac Ann. 2016;24:344-9.

821. de Jong E, van Oers JA, Beishuizen A, Vos P, Vermeijden WJ, Haas LE et al. Efficacy and safety of procalcitonin guidance in reducing the duration of antibiotic treatment in critically ill patients: a randomised, controlled, open-label trial. Lancet Infect Dis. 2016;16:819-27.

822. Hansen MB, Rasmussen LS, Garred P, Bidstrup D, Madsen MB, Hyldegaard O. Pentraxin-3 as a marker of disease severity and risk of death in patients with necrotizing soft tissue infections: a nationwide, prospective, observational study. Crit Care. 2016;20:40.

823. Demissei BG, Cleland JG, O'Connor CM, Metra M, Ponikowski P, Teerlink JR et al. Procalcitonin-based indication of bacterial infection identifies high risk acute heart failure patients. Int J Cardiol. 2016;204:164-71.

824. Laukemann S, Kasper N, Kulkarni P, Steiner D, Rast AC, Kutz A et al. Can we reduce negative blood cultures with clinical scores and blood markers? Results from an observational cohort study. Medicine (Baltimore). 2015;94:e2264.

825. Oussalah A, Ferrand J, Filhine-Tresarrieu P, Aissa N, Aimone-Gastin I, Namour F et al. Diagnostic accuracy of procalcitonin for predicting blood culture results in patients with suspected bloodstream infection: An observational study of 35,343 consecutive patients (A STROBE-Compliant Article). Medicine (Baltimore). 2015;94:e1774.

826. Najafi A, Khodadadian A, Sanatkar M, Shariat Moharari R, Etezadi F, Ahmadi A et al. The comparison of procalcitonin guidance administer antibiotics with empiric antibiotic therapy in critically ill patients admitted in intensive care unit. Acta Med Iran. 2015;53:562-7.

827. Mandell IM, Aghamohammadi S, Deakers T, Khemani RG. Procalcitonin to detect suspected bacterial infections in the PICU. Pediatr Crit Care Med. 2016;17:e4-12.

828. Dai X, Fu C, Wang C, Cai Y, Zhang S, Guo W et al. The impact of tracheotomy on levels of procalcitonin in patients without sepsis: a prospective study. Clinics (Sao Paulo). 2015;70:612-7.

829. Liu Z, Chen J, Liu Y, Si X, Jiang Z, Zhang X et al. A simple bioscore improves diagnostic accuracy of sepsis after surgery. J Surg Res. 2016;200:290-7.

830. Guo SY, Zhou Y, Hu QF, Yao J, Wang H. Procalcitonin is a marker of gram-negative bacteremia in patients with sepsis. Am J Med Sci. 2015;349:499-504.

831. Chakravarthy M, Kavaraganahalli D, Pargaonkar S, Hosur R, Harivelam C, Bharadwaj A et al. Elevated postoperative serum procalcitonin is not indicative of bacterial infection in cardiac surgical patients. Ann Card Anaesth. 2015;18:210-4.

832. Ishikura H, Nakamura Y, Kawano Y, Tanaka J, Mizunuma M, Ohta D et al. Intravenous immunoglobulin improves sepsis-induced coagulopathy: A retrospective, single-center observational study. J Crit Care. 2015;30:579-83.

833. Lee WS, Kang DW, Back JH, Kim HL, Chung JH, Shin BC. Cutoff value of serum procalcitonin as a diagnostic biomarker of infection in end-stage renal disease patients. Korean J Intern Med. 2015;30:198-204.

834. Anand D, Das S, Bhargava S, Srivastava LM, Garg A, Tyagi N et al. Procalcitonin as a rapid diagnostic biomarker to differentiate between culture-negative bacterial sepsis and systemic inflammatory response syndrome: a prospective, observational, cohort study. J Crit Care. 2015;30:218-12.

835. Shehabi Y, Sterba M, Garrett PM, Rachakonda KS, Stephens D, Harrigan P et al. Procalcitonin algorithm in critically ill adults with undifferentiated infection or suspected sepsis: A randomized controlled trial. Am J Respir Crit Care Med. 2014;190:1102-10.

836. Maseda E, Suarez-de-la-Rica A, Anillo V, Tamayo E, Garcia-Bernedo CA, Ramasco F et al. Procalcitonin-guided therapy may reduce length of antibiotic treatment in intensive care unit patients with secondary peritonitis: A multicenter retrospective study. J Crit Care. 2015;30:537-42.

837. Park DW, Kwak DS, Park YY, Chang Y, Huh JW, Lim CM et al. Impact of serial measurements of lysophosphatidylcholine on 28-day mortality prediction in patients admitted to the intensive care unit with severe sepsis or septic shock. J Crit Care. 2014;29:882-11.

838. Mahajan P, Grzybowski M, Chen X, Kannikeswaran N, Stanley R, Singal B et al. Procalcitonin as a marker of serious bacterial infections in febrile children younger than 3 years old. Acad Emerg Med. 2014;21:171-9.

839. Seoane L, Pertega S, Galeiras R, Astola I, Bouza T. Procalcitonin in the burn unit and the diagnosis of infection. Burns. 2014;40:223-9.

840. Masson S, Caironi P, Spanuth E, Thomae R, Panigada M, Sangiorgi G et al. Presepsin (soluble CD14 subtype) and procalcitonin levels for mortality prediction in sepsis: data from the Albumin Italian Outcome Sepsis trial. Crit Care. 2014;18:R6.

841. Jung B, Molinari N, Nasri M, Hajjej Z, Chanques G, Jean-Pierre H et al. Procalcitonin biomarker kinetics fails to predict treatment response in perioperative abdominal infection with septic shock. Crit Care. 2013;17:R255.

842. Dumea R, Siriopol D, Hogas S, Mititiuc I, Covic A. Procalcitonin: diagnostic value in systemic infections in chronic kidney disease or renal transplant patients. Int Urol Nephrol. 2014;46:461-8.

843. Schuetz P, Maurer P, Punjabi V, Desai A, Amin DN, Gluck E. Procalcitonin decrease over 72 hours in US critical care units predicts fatal outcome in sepsis patients. Crit Care. 2013;17:R115.

844. Ha YE, Kang CI, Wi YM, Chung DR, Kang ES, Lee NY et al. Diagnostic usefulness of procalcitonin as a marker of bacteremia in patients with acute pyelonephritis. Scand J Clin Lab Invest. 2013;73:444-8.

845. Deliberato RO, Marra AR, Sanches PR, Martino MD, Ferreira CE, Pasternak J et al. Clinical and economic impact of procalcitonin to shorten antimicrobial therapy in septic patients with proven bacterial infection in an intensive care setting. Diagn Microbiol Infect Dis. 2013;76:266-71.

846. Zhao Y, Li C, Jia Y. Evaluation of the Mortality in Emergency Department Sepsis score combined with procalcitonin in septic patients. Am J Emerg Med. 2013;31:1086-91.

847. Park JH, Wee JH, Choi SP, Park KN. Serum procalcitonin level for the prediction of severity in women with acute pyelonephritis in the ED: value of procalcitonin in acute pyelonephritis. Am J Emerg Med. 2013;31:1092-7.

848. Hicks CW, Engineer RS, Benoit JL, Dasarathy S, Christenson RH, Peacock WF. Procalcitonin as a biomarker for early sepsis in the emergency department. Eur J Emerg Med. 2014;21:112-7.

849. Azevedo JR, Torres OJ, Czeczko NG, Tuon FF, Nassif PA, Souza GD. Procalcitonin as a prognostic biomarker of severe sepsis and septic shock. Rev Col Bras Cir. 2012;39:456-61.

850. Schlapbach LJ, Graf R, Woerner A, Fontana M, Zimmermann-Baer U, Glauser D et al. Pancreatic stone protein as a novel marker for neonatal sepsis. Intensive Care Med. 2013;39:754-63.

851. Kasem AJ, Bulloch B, Henry M, Shah K, Dalton H. Procalcitonin as a marker of bacteremia in children with fever and a central venous catheter presenting to the emergency department. Pediatr Emerg Care. 2012;28:1017-21.

852. Jensen JU, Hein L, Lundgren B, Bestle MH, Mohr TT, Andersen MH et al. Procalcitonin-guided interventions against infections to increase early appropriate antibiotics and improve survival in the intensive care unit: a randomized trial. Crit Care Med. 2011;39:2048-58.

853. Stocker M, Hop WC, van Rossum AM. Neonatal Procalcitonin Intervention Study (NeoPInS): Effect of Procalcitonin-guided decision making on duration of antibiotic therapy in suspected neonatal early-onset sepsis: A multi-centre randomized superiority and non-inferiority Intervention Study. BMC Pediatr. 2010;10:89.

854. Picariello C, Lazzeri C, Valente S, Chiostri M, Attana P, Gensini GF. Kinetics of procalcitonin in cardiogenic shock and in septic shock. Preliminary data. Acute Card Care. 2010;12:96-101.

855. Muller F, Christ-Crain M, Bregenzer T, Krause M, Zimmerli W, Mueller B et al. Procalcitonin levels predict bacteremia in patients with community-acquired pneumonia: a prospective cohort trial. Chest. 2010;138:121-9.

856. Stocker M, Fontana M, El Helou S, Wegscheider K, Berger TM. Use of procalcitonin-guided decision-making to shorten antibiotic therapy in suspected neonatal early-onset sepsis: prospective randomized intervention trial. Neonatology. 2010;97:165-74.

857. Sand M, Trullen XV, Bechara FG, Pala XF, Sand D, Landgrafe G et al. A prospective bicenter study investigating the diagnostic value of procalcitonin in patients with acute appendicitis. Eur Surg Res. 2009;43:291-7.

858. Hochreiter M, Kohler T, Schweiger AM, Keck FS, Bein B, von Spiegel T et al. Procalcitonin to guide duration of antibiotic therapy in intensive care patients: a randomized prospective controlled trial. Crit Care. 2009;13:R83.

859. Kuse ER, Langefeld I, Jaeger K, Kulpmann WR. Procalcitonin-a new diagnostic tool in complications following liver transplantation. Intensive Care Med. 2000;26 Suppl 2:S187-S192.

860. Hochreiter M, Kohler T, Schweiger AM, Keck FS, Bein B, von Spiegel T et al. [Antibiotic treatment of surgical intensive care patients: procalcitonin to guide duration of therapy]. Anaesthesist. 2008;57:571-7.

861. Svoboda P, Kantorova I, Scheer P, Radvanova J, Radvan M. Can procalcitonin help us in timing of re-intervention in septic patients after multiple trauma or major surgery? Hepatogastroenterology. 2007;54:359-63.

862. Chiesa C, Pellegrini G, Panero A, Osborn JF, Signore F, Assumma M et al. C-reactive protein, interleukin-6, and procalcitonin in the immediate postnatal period: influence of illness severity, risk status, antenatal and perinatal complications, and infection. Clin Chem. 2003;49:60-8.

863. Shimetani N, Ohba Y, Shimetani K, Mashiko T, Matsuyama N, Ohtani H et al. [Assay for determination of the serum procalcitonin level: biochemical and clinical evaluation]. Rinsho Byori. 2001;49:56-60.

864. Boeken U, Feindt P, Petzold T, Klein M, Micek M, Seyfert UT et al. Diagnostic value of procalcitonin: the influence of cardiopulmonary bypass, aprotinin, SIRS, and sepsis. Thorac Cardiovasc Surg. 1998;46:348-51.

865. Gerard Y, Hober D, Assicot M, Alfandari S, Ajana F, Bourez JM et al. Procalcitonin as a marker of bacterial sepsis in patients infected with HIV-1. J Infect. 1997;35:41-6.

866. Cabral L, Afreixo V, Meireles R, Vaz M, Frade JG, Chaves C et al. Evaluation of procalcitonin accuracy for the distinction between gram-negative and gram-positive bacterial sepsis in burn patients. J Burn Care Res. 2019;40:112-9.

867. Nakamura Y, Hoshino K, Kiyomi F, Kawano Y, Mizunuma M, Tanaka J et al. Comparison of accuracy of presepsin and procalcitonin concentrations in diagnosing sepsis in patients with and without acute kidney injury. Clin Chim Acta. 2019;490:200-6.

868. Gutierrez-Gutierrez B, Morales I, Perez-Galera S, Fernandez-Riejos P, Retamar P, de Cueto M et al. Predictive value of the kinetics of procalcitonin and C-reactive protein for early clinical stability in patients with bloodstream infections due to Gram-negative bacteria. Diagn Microbiol Infect Dis. 2019;93:63-8.

869. Luo X, Yang X, Li J, Zou G, Lin Y, Qing G et al. The procalcitonin/albumin ratio as an early diagnostic predictor in discriminating urosepsis from patients with febrile urinary tract infection. Medicine (Baltimore). 2018;97:e11078.

870. Kim SJ, Hwang SO, Kim YW, Lee JH, Cha KC. Procalcitonin as a diagnostic marker for sepsis/septic shock in the emergency department; a study based on Sepsis-3 definition. Am J Emerg Med. 2019;37:272-6.

871. Markanday A. Acute phase reactants in infections: Evidence-based review and a guide for clinicians. Open Forum Infect Dis. 2015;2:ofv098.

872. Hamed S, Behnes M, Pauly D, Lepiorz D, Barre M, Becher T et al. Diagnostic value of Pentraxin-3 in patients with sepsis and septic shock in accordance with latest sepsis-3 definitions. BMC Infect Dis. 2017;17:554.

873. al-Ramadi BK, Ellis M, Pasqualini F, Mantovani A. Selective induction of pentraxin 3, a soluble innate immune pattern recognition receptor, in infectious episodes in patients with haematological malignancy. Clin Immunol. 2004;112:221-4.

874. Bastrup-Birk S, Skjoedt MO, Munthe-Fog L, Strom JJ, Ma YJ, Garred P. Pentraxin-3 serum levels are associated with disease severity and mortality in patients with systemic inflammatory response syndrome. PLoS One. 2013;8:e73119.

875. Caironi P, Masson S, Mauri T, Bottazzi B, Leone R, Magnoli M et al. Pentraxin 3 in patients with severe sepsis or shock: the ALBIOS trial. Eur J Clin Invest. 2017;47:73-83.

876. Cuello F, Shankar-Hari M, Mayr U, Yin X, Marshall M, Suna G et al. Redox state of pentraxin 3 as a novel biomarker for resolution of inflammation and survival in sepsis. Mol Cell Proteomics. 2014;13:2545-57.

877. Hu C, Zhou Y, Liu C, Kang Y. Pentraxin-3, procalcitonin and lactate as prognostic markers in patients with sepsis and septic shock. Oncotarget. 2018;9:5125-36.

878. Jie H, Li Y, Pu X, Ye J. Pentraxin 3, a predicator for 28-day mortality in patients with septic shock. Am J Med Sci. 2017;353:242-6.

879. Kim SB, Lee KH, Lee JU, Ann HW, Ahn JY, Jeon YD et al. Long pentraxin 3 as a predictive marker of mortality in severe septic patients who received successful early goal-directed therapy. Yonsei Med J. 2017;58:370-9.

880. Lin Q, Fu F, Shen L, Zhu B. Pentraxin 3 in the assessment of ventilator-associated pneumonia: an early marker of severity. Heart Lung. 2013;42:139-45.

881. Liu S, Qu X, Liu F, Wang C. Pentraxin 3 as a prognostic biomarker in patients with systemic inflammation or infection. Mediators Inflamm. 2014;2014:421429.

882. Mauri T, Bellani G, Patroniti N, Coppadoro A, Peri G, Cuccovillo I et al. Persisting high levels of plasma pentraxin 3 over the first days after severe sepsis and septic shock onset are associated with mortality. Intensive Care Med. 2010;36:621-9.

883. Muller B, Peri G, Doni A, Torri V, Landmann R, Bottazzi B et al. Circulating levels of the long pentraxin PTX3 correlate with severity of infection in critically ill patients. Crit Care Med. 2001;29:1404-7.

884. Thulborn SJ, Dilpazir M, Haldar K, Mistry V, Brightling CE, Barer MR et al. Investigating the role of pentraxin 3 as a biomarker for bacterial infection in subjects with COPD. Int J Chron Obstruct Pulmon Dis. 2017;12:1199-205.

885. Vanska M, Koivula I, Hamalainen S, Pulkki K, Nousiainen T, Jantunen E et al. High pentraxin 3 level predicts septic shock and bacteremia at the onset of febrile neutropenia after intensive chemotherapy of hematologic patients. Haematologica. 2011;96:1385-9.

886. Mantovani A, Garlanda C, Doni A, Bottazzi B. Pentraxins in innate immunity: from C-reactive protein to the long pentraxin PTX3. J Clin Immunol. 2008;28:1-13.

887. Gressner OA, Koch A, Sanson E, Trautwein C, Tacke F. High C5a levels are associated with increased mortality in sepsis patients--no enhancing effect by actin-free Gc-globulin. Clin Biochem. 2008;41:974-80.

888. Hecke F, Schmidt U, Kola A, Bautsch W, Klos A, Kohl J. Circulating complement proteins in multiple trauma patients--correlation with injury severity, development of sepsis, and outcome. Crit Care Med. 1997;25:2015-24.

889. Stove S, Welte T, Wagner TO, Kola A, Klos A, Bautsch W et al. Circulating complement proteins in patients with sepsis or systemic inflammatory response syndrome. Clin Diagn Lab Immunol. 1996;3:175-83.

890. Lendak D, Mihajlovic D, Mitic G, Ubavic M, Novakov-Mikic A, Boban J et al. Complement component consumption in sepsis correlates better with hemostatic system parameters than with inflammatory biomarkers. Thromb Res. 2018;170:126-32.

891. Charchaflieh J, Rushbrook J, Worah S, Zhang M. Activated complement factors as disease markers for sepsis. Dis Markers. 2015;2015:382463.

892. Delogu G, Lo Bosco L, Marandola M, Famularo G, Lenti L, Ippoliti F et al. Heat shock protein (HSP70) expression in septic patients. J Crit Care. 1997;12:188-92.

893. Ofenstein JP, Heidemann S, Juett-Wilstermann A, Sarnaik A. Expression of stress proteins HSP 72 & HSP 32 in response to endotoxemia. Ann Clin Lab Sci. 2000;30:92-8.

894. Ramaglia V, Harapa GM, White N, Buck LT. Bacterial infection and tissue-specific Hsp72, -73 and -90 expression in western painted turtles. Comp Biochem Physiol C Toxicol Pharmacol. 2004;138:139-48.

895. Weiss YG, Bouwman A, Gehan B, Schears G, Raj N, Deutschman CS. Cecal ligation and double puncture impairs heat shock protein 70 (HSP-70) expression in the lungs of rats. Shock. 2000;13:19-23.

896. Wheeler DS, Fisher LE, Jr., Catravas JD, Jacobs BR, Carcillo JA, Wong HR. Extracellular hsp70 levels in children with septic shock. Pediatr Crit Care Med. 2005;6:308-11.

897. Qu B, Jia Y, Liu Y, Wang H, Ren G, Wang H. The detection and role of heat shock protein 70 in various nondisease conditions and disease conditions: a literature review. Cell Stress Chaperones. 2015;20:885-92.

898. Gukasjan R, Raptis DA, Schulz HU, Halangk W, Graf R. Pancreatic stone protein predicts outcome in patients with peritonitis in the ICU. Crit Care Med. 2013;41:1027-36.

899. Jiri Z, Kyr M, Vavrina M, Fedora M. Pancreatic stone protein - a possible biomarker of multiorgan failure and mortality in children sepsis. Cytokine. 2014;66:106-11.

900. Klein HJ, Csordas A, Falk V, Slankamenac K, Rudiger A, Schonrath F et al. Pancreatic stone protein predicts postoperative infection in cardiac surgery patients irrespective of cardiopulmonary bypass or surgical technique. PLoS One. 2015;10:e0120276.

901. Palmiere C, Augsburger M. Pancreatic stone protein as a postmortem biochemical marker for the diagnosis of sepsis. Leg Med (Tokyo). 2015;17:9-13.

902. Que YA, Delodder F, Guessous I, Graf R, Bain M, Calandra T et al. Pancreatic stone protein as an early biomarker predicting mortality in a prospective cohort of patients with sepsis requiring ICU management. Crit Care. 2012;16:R114.

903. Tribl B, Filipp D, Bodeker H, Yu P, Hammerrmuller I, McKerlie C et al. Pseudomonas pneumonia-mediated sepsis induces expression of pancreatitis-associated protein-I in rat pancreas. Pancreas. 2004;29:33-40.

904. Wu R, Zha L, Hu JH, Wu JM, Tian ZF. The value of pancreatic stone protein in the prediction of infected neonates. Minerva Pediatr. 2017;69:489-94.

905. Bimmler D, Schiesser M, Perren A, Scheele G, Angst E, Meili S et al. Coordinate regulation of PSP/reg and PAP isoforms as a family of secretory stress proteins in an animal model of chronic pancreatitis. J Surg Res. 2004;118:122-35.

906. Karampela I, Kandri E, Antonakos G, Vogiatzakis E, Christodoulatos GS, Nikolaidou A et al. Kinetics of circulating fetuin-A may predict mortality independently from adiponectin, high molecular weight adiponectin and prognostic factors in critically ill patients with sepsis: A prospective study. J Crit Care. 2017;41:78-85.

907. Li W, Zhu S, Li J, Huang Y, Zhou R, Fan X et al. A hepatic protein, fetuin-A, occupies a protective role in lethal systemic inflammation. PLoS One. 2011;6:e16945.

908. Berbee JF, van der Hoogt CC, de Haas CJ, van Kessel KP, Dallinga-Thie GM, Romijn JA et al. Plasma apolipoprotein CI correlates with increased survival in patients with severe sepsis. Intensive Care Med. 2008;34:907-11.

909. Schippers EF, Berbee JF, van Disseldorp IM, Versteegh MI, Havekes LM, Rensen PC et al. Preoperative apolipoprotein CI levels correlate positively with the proinflammatory response in patients experiencing endotoxemia following elective cardiac surgery. Intensive Care Med. 2008;34:1492-7.

910. Berbee JF, van der Hoogt CC, Kleemann R, Schippers EF, Kitchens RL, van Dissel JT et al. Apolipoprotein CI stimulates the response to lipopolysaccharide and reduces mortality in gram-negative sepsis. FASEB J. 2006;20:2162-4.

911. Forceville X, Mostert V, Pierantoni A, Vitoux D, Le TP, Plouvier E et al. Selenoprotein P, rather than glutathione peroxidase, as a potential marker of septic shock and related syndromes. Eur Surg Res. 2009;43:338-47.

912. Hollenbach B, Morgenthaler NG, Struck J, Alonso C, Bergmann A, Kohrle J et al. New assay for the measurement of selenoprotein P as a sepsis biomarker from serum. J Trace Elem Med Biol. 2008;22:24-32.

913. Lee WJ, Chen YL, Chu YW, Chien DS. Comparison of glutathione peroxidase-3 protein expression and enzyme bioactivity in normal subjects and patients with sepsis. Clin Chim Acta. 2019;489:177-82.

914. Burk RF, Hill KE. Selenoprotein P: an extracellular protein with unique physical characteristics and a role in selenium homeostasis. Annu Rev Nutr. 2005;25:215-35.

915. Alcoba G, Manzano S, Lacroix L, Galetto-Lacour A, Gervaix A. Proadrenomedullin and copeptin in pediatric pneumonia: a prospective diagnostic accuracy study. BMC Infect Dis. 2015;15:347.

916. Battista S, Audisio U, Galluzzo C, Maggiorotto M, Masoero M, Forno D et al. Assessment of diagnostic and prognostic role of copeptin in the clinical setting of sepsis. Biomed Res Int. 2016;2016:3624730.

917. Jiang L, Feng B, Gao D, Zhang Y. Plasma concentrations of copeptin, C-reactive protein and procalcitonin are positively correlated with APACHE II scores in patients with sepsis. J Int Med Res. 2015;43:188-95.

918. Lee JH, Chan YH, Lai OF, Puthucheary J. Vasopressin and copeptin levels in children with sepsis and septic shock. Intensive Care Med. 2013;39:747-53.

919. Muller B, Morgenthaler N, Stolz D, Schuetz P, Muller C, Bingisser R et al. Circulating levels of copeptin, a novel biomarker, in lower respiratory tract infections. Eur J Clin Invest. 2007;37:145-52.

920. Purhonen AK, Vanska M, Hamalainen S, Pulkki K, Lehtikangas M, Kuittinen T et al. Plasma copeptin in the assessment of febrile neutropenia. Peptides. 2012;36:129-32.

921. Seligman R, Papassotiriou J, Morgenthaler NG, Meisner M, Teixeira PJ. Copeptin, a novel prognostic biomarker in ventilator-associated pneumonia. Crit Care. 2008;12:R11.

922. Lukaszyk E, Malyszko J. Copeptin: Pathophysiology and potential clinical impact. Adv Med Sci. 2015;60:335-41.

923. Dahl B, Schiodt FV, Ott P, Wians F, Lee WM, Balko J et al. Plasma concentration of Gc-globulin is associated with organ dysfunction and sepsis after injury. Crit Care Med. 2003;31:152-6.

924. Lee WM, Galbraith RM. The extracellular actin-scavenger system and actin toxicity. N Engl J Med. 1992;326:1335-41.

925. Zhou Y, Dong H, Zhong Y, Huang J, Lv J, Li J. The Cold-Inducible RNA-Binding Protein (CIRP) level in peripheral blood predicts sepsis outcome. PLoS One. 2015;10:e0137721.

926. Yang WL, Sharma A, Wang Z, Li Z, Fan J, Wang P. Cold-inducible RNA-binding protein causes endothelial dysfunction via activation of Nlrp3 inflammasome. Sci Rep. 2016;6:26571.

927. Donnino MW, Cocchi MN, Salciccioli JD, Kim D, Naini AB, Buettner C et al. Coenzyme Q10 levels are low and may be associated with the inflammatory cascade in septic shock. Crit Care. 2011;15:R189.

928. Acosta MJ, Vazquez Fonseca L, Desbats MA, Cerqua C, Zordan R, Trevisson E et al. Coenzyme Q biosynthesis in health and disease. Biochim Biophys Acta. 2016;1857:1079-85.

929. Montuschi P, Barnes PJ, Roberts LJ. Isoprostanes: markers and mediators of oxidative stress. FASEB J. 2004;18:1791-800.

930. Puthucheary SD, Nathan SA. Comparison of serum F2 isoprostane levels in diabetic patients and diabetic patients infected with Burkholderia pseudomallei. Singapore Med J. 2008;49:117-20.

931. Ware LB, Fessel JP, May AK, Roberts LJ. Plasma biomarkers of oxidant stress and development of organ failure in severe sepsis. Shock. 2011;36:12-7.

932. Ploder M, Kurz K, Spittler A, Neurauter G, Roth E, Fuchs D. Early increase of plasma homocysteine in sepsis patients with poor outcome. Mol Med. 2010;16:498-504.

933. Schroecksnadel K, Frick B, Winkler C, Leblhuber F, Wirleitner B, Fuchs D. Hyperhomocysteinemia and immune activation. Clin Chem Lab Med. 2003;41:1438-43.

934. Lorente L, Martin MM, Abreu-Gonzalez P, Dominguez-Rodriguez A, Labarta L, Diaz C et al. Prognostic value of malondialdehyde serum levels in severe sepsis: a multicenter study. PLoS One. 2013;8:e53741.

935. Akbar SR, Long DM, Hussain K, Alhajhusain A, Ahmed US, Iqbal HI et al. Hyperuricemia: an early marker for severity of illness in sepsis. Int J Nephrol. 2015;2015:301021.

936. Giovannini I, Chiarla C, Giuliante F, Vellone M, Ardito F, Pallavicini F et al. Biochemical and clinical correlates of hypouricemia in surgical and critically ill patients. Clin Chem Lab Med. 2007;45:1207-10.

937. Montero-Chacon LB, Padilla-Cuadra JI, Chiou SH, Torrealba-Acosta G. High-density lipoprotein, mean platelet volume, and uric acid as biomarkers for outcomes in patients with sepsis: An observational study. J Intensive Care Med. 2018;885066618772825.

938. Mazzali M, Hughes J, Kim YG, Jefferson JA, Kang DH, Gordon KL et al. Elevated uric acid increases blood pressure in the rat by a novel crystal-independent mechanism. Hypertension. 2001;38:1101-6.

939. Pascual C, Karzai W, Meier-Hellmann A, Oberhoffer M, Horn A, Bredle D et al. Total plasma antioxidant capacity is not always decreased in sepsis. Crit Care Med. 1998;26:705-9.

940. Yao L, Liu Z, Zhu J, Li B, Chai C, Tian Y. Higher serum level of myoglobin could predict more severity and poor outcome for patients with sepsis. Am J Emerg Med. 2016;34:948-52.

941. Sakurai S, Kuroko Y, Shimizu S, Kawada T, Akiyama T, Yamazaki T et al. Effects of intravenous cariporide on release of norepinephrine and myoglobin during myocardial ischemia/reperfusion in rabbits. Life Sci. 2014;114:102-6.

942. Cheng WE, Shih CM, Hang LW, Wu KY, Yang HL, Hsu WH et al. Urinary biomarker of oxidative stress correlating with outcome in critically septic patients. Intensive Care Med. 2007;33:1468-72.

943. Shigenaga MK, Ames BN. Assays for 8-hydroxy-2'-deoxyguanosine: a biomarker of in vivo oxidative DNA damage. Free Radic Biol Med. 1991;10:211-6.

944. Otani K, Shimizu S, Chijiiwa K, Yamaguchi K, Kuroki S, Tanaka M. Increased urinary excretion of bilirubin oxidative metabolites in septic patients: a new marker for oxidative stress in vivo. J Surg Res. 2001;96:44-9.

945. Yamaguchi T, Shioji I, Sugimoto A, Komoda Y, Nakajima H. Chemical structure of a new family of bile pigments from human urine. J Biochem. 1994;116:298-303.

946. Biller K, Fae P, Germann R, Drexel H, Walli AK, Fraunberger P. Cholesterol rather than procalcitonin or C-reactive protein predicts mortality in patients with infection. Shock. 2014;42:129-32.

947. Chien JY, Jerng JS, Yu CJ, Yang PC. Low serum level of high-density lipoprotein cholesterol is a poor prognostic factor for severe sepsis. Crit Care Med. 2005;33:1688-93.

948. Cirstea M, Walley KR, Russell JA, Brunham LR, Genga KR, Boyd JH. Decreased high-density lipoprotein cholesterol level is an early prognostic marker for organ dysfunction and death in patients with suspected sepsis. J Crit Care. 2017;38:289-94.

949. Guirgis FW, Donnelly JP, Dodani S, Howard G, Safford MM, Levitan EB et al. Cholesterol levels and long-term rates of community-acquired sepsis. Crit Care. 2016;20:408.

950. Lagrost L, Girard C, Grosjean S, Masson D, Deckert V, Gautier T et al. Low preoperative cholesterol level is a risk factor of sepsis and poor clinical outcome in patients undergoing cardiac surgery with cardiopulmonary bypass. Crit Care Med. 2014;42:1065-73.

951. Memis D, Gursoy O, Tasdogan M, Sut N, Kurt I, Ture M et al. High C-reactive protein and low cholesterol levels are prognostic markers of survival in severe sepsis. J Clin Anesth. 2007;19:186-91.

952. Yamano S, Shimizu K, Ogura H, Hirose T, Hamasaki T, Shimazu T et al. Low total cholesterol and high total bilirubin are associated with prognosis in patients with prolonged sepsis. J Crit Care. 2016;31:36-40.

953. Lappalainen M, Jokkala J, Juutilainen A, Hamalainen S, Koivula I, Jantunen E et al. Novel biomarker candidates for febrile neutropenia in hematological patients using nontargeted metabolomics. Dis Markers. 2018;2018:6964529.

954. Larson MC, Woodliff JE, Hillery CA, Kearl TJ, Zhao M. Phosphatidylethanolamine is externalized at the surface of microparticles. Biochim Biophys Acta. 2012;1821:1501-7.

955. Meinhold H, Gramm HJ, Meissner W, Zimmermann J, Schwander J, Dennhardt R et al. Elevated serum diiodotyrosine (DIT) in severe infections and sepsis: DIT, a possible new marker of leukocyte activity. J Clin Endocrinol Metab. 1991;72:945-53.

956. Burger AG, Engler D, Buergi U, Weissel M, Steiger G, Ingbar SH et al. Ether link cleavage is the major pathway of iodothyronine metabolism in the phagocytosing human leukocyte and also occurs in vivo in the rat. J Clin Invest. 1983;71:935-49.

957. Tamion F, Le Cam-Duchez V, Menard JF, Girault C, Coquerel A, Bonmarchand G. Erythropoietin and renin as biological markers in critically ill patients. Crit Care. 2004;8:R328-R335.

958. Jelkmann W. Erythropoietin: structure, control of production, and function. Physiol Rev. 1992;72:449-89.

959. Yilmaz Z, Ilcol YO, Ulus IH. Endotoxin increases plasma leptin and ghrelin levels in dogs. Crit Care Med. 2008;36:828-33.

960. Bornstein SR, Licinio J, Tauchnitz R, Engelmann L, Negrao AB, Gold P et al. Plasma leptin levels are increased in survivors of acute sepsis: associated loss of diurnal rhythm, in cortisol and leptin secretion. J Clin Endocrinol Metab. 1998;83:280-3.

961. Grigoras I, Branisteanu DD, Ungureanu D, Rusu D, Ristescu I. Early dynamics of leptin plasma level in surgical critically ill patients. a prospective comparative study. Chirurgia (Bucur). 2014;109:66-72.

962. Tzanela M, Orfanos SE, Tsirantonaki M, Kotanidou A, Sotiropoulou C, Christophoraki M et al. Leptin alterations in the course of sepsis in humans. In Vivo. 2006;20:565-70.

963. Yousef AA, Amr YM, Suliman GA. The diagnostic value of serum leptin monitoring and its correlation with tumor necrosis factor-alpha in critically ill patients: a prospective observational study. Crit Care. 2010;14:R33.

964. Sanchez-Margalet V, Martin-Romero C, Santos-Alvarez J, Goberna R, Najib S, Gonzalez-Yanes C. Role of leptin as an immunomodulator of blood mononuclear cells: mechanisms of action. Clin Exp Immunol. 2003;133:11-9.

965. Koch A, Sanson E, Voigt S, Helm A, Trautwein C, Tacke F. Serum adiponectin upon admission to the intensive care unit may predict mortality in critically ill patients. J Crit Care. 2011;26:166-74.

966. Teoh H, Quan A, Bang KW, Wang G, Lovren F, Vu V et al. Adiponectin deficiency promotes endothelial activation and profoundly exacerbates sepsis-related mortality. Am J Physiol Endocrinol Metab. 2008;295:E658-E664.

967. Skott O. Renin. Am J Physiol Regul Integr Comp Physiol. 2002;282:R937-R939.

968. Gao M, Zhang L, Liu Y, Yang M, Wang N, Wang K et al. Use of blood urea nitrogen, creatinine, interleukin-6, granulocyte-macrophage colony stimulating factor in combination to predict the severity and outcome of abdominal sepsis in rats. Inflamm Res. 2012;61:889-97.

969. Kennon C, Overturf G, Bessman S, Sierra E, Smith KJ, Brann B. Granulocyte colony-stimulating factor as a marker for bacterial infection in neonates. J Pediatr. 1996;128:765-9.

970. Perry SE, Mostafa SM, Wenstone R, McLaughlin PJ. Low plasma granulocyte-macrophage colony stimulating factor is an indicator of poor prognosis in sepsis. Intensive Care Med. 2002;28:981-4.

971. Presneill JJ, Waring PM, Layton JE, Maher DW, Cebon J, Harley NS et al. Plasma granulocyte colony-stimulating factor and granulocyte-macrophage colony-stimulating factor levels in

critical illness including sepsis and septic shock: relation to disease severity, multiple organ dysfunction, and mortality. Crit Care Med. 2000;28:2344-54.

972. Russell JA, Fjell C, Hsu JL, Lee T, Boyd J, Thair S et al. Vasopressin compared with norepinephrine augments the decline of plasma cytokine levels in septic shock. Am J Respir Crit Care Med. 2013;188:356-64.

973. Barth E, Fischer G, Schneider EM, Moldawer LL, Georgieff M, Weiss M. Peaks of endogenous G-CSF serum concentrations are followed by an increase in respiratory burst activity of granulocytes in patients with septic shock. Cytokine. 2002;17:275-84.

974. Gaddnas F, Koskela M, Koivukangas V, Risteli J, Oikarinen A, Laurila J et al. Markers of collagen synthesis and degradation are increased in serum in severe sepsis: a longitudinal study of 44 patients. Crit Care. 2009;13:R53.

975. Zakynthinos S, Papanikolaou S, Mentzelopoulos S, Konstandelou E, Psachoulia C, Mavrommatis A. Procollagen type III aminoterminal propeptide as biomarker of host response in severe sepsis. J Crit Care. 2013;28:577-85.

976. Waydhas C, Nast-Kolb D, Trupka A, Lenk S, Duswald KH, Schweiberer L et al. Increased serum concentrations of procollagen type III peptide in severely injured patients: an indicator of fibrosing activity? Crit Care Med. 1993;21:240-7.

977. Shin J, Hwang SY, Jo IJ, Kim WY, Ryoo SM, Kang GH et al. Prognostic value of the lactate/albumin ratio for predicting 28-day mortality in critically ill sepsis patients. Shock. 2018;50:545-50.

978. Wang XY, Li WQ, Lu J, Li N, Li JS. Lipopolysaccharide decreasing albumin expression in rat hepatocytes. Hepatobiliary Pancreat Dis Int. 2005;4:410-5.

979. Yin M, Si L, Qin W, Li C, Zhang J, Yang H et al. Predictive value of serum albumin level for the prognosis of severe sepsis without exogenous human albumin administration: A prospective cohort study. J Intensive Care Med. 2018;33:687-94.

980. Takegawa R, Kabata D, Shimizu K, Hisano S, Ogura H, Shintani A et al. Serum albumin as a risk factor for death in patients with prolonged sepsis: An observational study. J Crit Care. 2019;51:139-44.

981. Kendall H, Abreu E, Cheng AL. Serum albumin trend is a predictor of mortality in ICU patients with sepsis. Biol Res Nurs. 2019;21:237-44.

982. Fleck A, Raines G, Hawker F, Trotter J, Wallace PI, Ledingham IM et al. Increased vascular permeability: a major cause of hypoalbuminaemia in disease and injury. Lancet. 1985;1:781-4.

983. Weiss M, Elsharkawi M, Welt K, Schneider EM. Transient leukocytosis, granulocyte colony-stimulating factor plasma concentrations, and apoptosis determined by binding of annexin V by peripheral leukocytes in patients with severe sepsis. Ann N Y Acad Sci. 2003;1010:742-7.

984. Reutelingsperger CP, van Heerde WL. Annexin V, the regulator of phosphatidylserine-catalyzed inflammation and coagulation during apoptosis. Cell Mol Life Sci. 1997;53:527-32.

985. Maury E, Blanchard HS, Chauvin P, Guglielminotti J, Alzieu M, Guidet B et al. Circulating endotoxin and antiendotoxin antibodies during severe sepsis and septic shock. J Crit Care. 2003;18:115-20.

986. Strutz F, Heller G, Krasemann K, Krone B, Muller GA. Relationship of antibodies to endotoxin core to mortality in medical patients with sepsis syndrome. Intensive Care Med. 1999;25:435-44.

987. Rothenburger M, Soeparwata R, Deng MC, Berendes E, Schmid C, Tjan TD et al. The impact of anti-endotoxin core antibodies on endotoxin and cytokine release and ventilation time after cardiac surgery. J Am Coll Cardiol. 2001;38:124-30.

988. Rylatt D, Wilson K, Kemp BE, Elms MJ, Manickavasagam B, Shi W et al. A rapid test for endotoxin in whole blood. Prog Clin Biol Res. 1995;392:273-84.

989. Elsbach P. The bactericidal/permeability-increasing protein (BPI) in antibacterial host defense. J Leukoc Biol. 1998;64:14-8.

990. Ahmed AI, Soliman RA, Samir S. Cell free DNA and procalcitonin as early markers of complications in ICU patients with multiple trauma and major surgery. Clin Lab. 2016;62:2395-404.

991. Dwivedi DJ, Toltl LJ, Swystun LL, Pogue J, Liaw KL, Weitz JI et al. Prognostic utility and characterization of cell-free DNA in patients with severe sepsis. Crit Care. 2012;16:R151.

992. Forsblom E, Aittoniemi J, Ruotsalainen E, Helmijoki V, Huttunen R, Jylhava J et al. High cell-free DNA predicts fatal outcome among Staphylococcus aureus bacteraemia patients with intensive care unit treatment. PLoS One. 2014;9:e87741.

993. Garnacho-Montero J, Huici-Moreno MJ, Gutierrez-Pizarraya A, Lopez I, Marquez-Vacaro JA, Macher H et al. Prognostic and diagnostic value of eosinopenia, C-reactive protein,

procalcitonin, and circulating cell-free DNA in critically ill patients admitted with suspicion of sepsis. Crit Care. 2014;18:R116.

994. Hou YQ, Liang DY, Lou XL, Zhang M, Zhang ZH, Zhang LR. Branched DNA-based Alu quantitative assay for cell-free plasma DNA levels in patients with sepsis or systemic inflammatory response syndrome. J Crit Care. 2016;31:90-5.

995. Moreira VG, Prieto B, Rodriguez JS, Alvarez FV. Usefulness of cell-free plasma DNA, procalcitonin and C-reactive protein as markers of infection in febrile patients. Ann Clin Biochem. 2010;47:253-8.

996. Nguyen DN, Stensballe A, Lai JC, Jiang P, Brunse A, Li Y et al. Elevated levels of circulating cell-free DNA and neutrophil proteins are associated with neonatal sepsis and necrotizing enterocolitis in immature mice, pigs and infants. Innate Immun. 2017;23:524-36.

997. Purhonen AK, Juutilainen A, Vanska M, Lehtikangas M, Lakkisto P, Hamalainen S et al. Human plasma cell-free DNA as a predictor of infectious complications of neutropenic fever in hematological patients. Infect Dis (Lond). 2015;47:255-9.

998. Rhodes A, Wort SJ, Thomas H, Collinson P, Bennett ED. Plasma DNA concentration as a predictor of mortality and sepsis in critically ill patients. Crit Care. 2006;10:R60.

999. Vrazo AC, Hontz AE, Figueira SK, Butler BL, Ferrell JM, Binkowski BF et al. Live cell evaluation of granzyme delivery and death receptor signaling in tumor cells targeted by human natural killer cells. Blood. 2015;126:e1-e10.

1000. Duplessis C, Gregory M, Frey K, Bell M, Truong L, Schully K et al. Evaluating the discriminating capacity of cell death (apoptotic) biomarkers in sepsis. J Intensive Care. 2018;6:72.

1001. Xin Y, Gao X, Wang W, Xu X, Yu L, Ju X et al. Circulating cell-free DNA indicates M1/M2 responses during septic peritonitis. Biochem Biophys Res Commun. 2016;477:589-94.

1002. Delogu G, Famularo G, Amati F, Signore L, Antonucci A, Trinchieri V et al. Ceramide concentrations in septic patients: a possible marker of multiple organ dysfunction syndrome. Crit Care Med. 1999;27:2413-7.

1003. Rozenova KA, Deevska GM, Karakashian AA, Nikolova-Karakashian MN. Studies on the role of acid sphingomyelinase and ceramide in the regulation of tumor necrosis factor alpha (TNFalpha)-converting enzyme activity and TNFalpha secretion in macrophages. J Biol Chem. 2010;285:21103-13.

1004. Chen Q, Ye L, Jin Y, Zhang N, Lou T, Qiu Z et al. Circulating nucleosomes as a predictor of sepsis and organ dysfunction in critically ill patients. Int J Infect Dis. 2012;16:e558-e564.

1005. Yehya N, Thomas NJ, Margulies SS. Circulating nucleosomes are associated with mortality in pediatric acute respiratory distress syndrome. Am J Physiol Lung Cell Mol Physiol. 2016;310:L1177-L1184.

1006. Zeerleder S, Zwart B, Wuillemin WA, Aarden LA, Groeneveld AB, Caliezi C et al. Elevated nucleosome levels in systemic inflammation and sepsis. Crit Care Med. 2003;31:1947-51.

1007. Patel P, Walborn A, Rondina M, Fareed J, Hoppensteadt D. Markers of Inflammation and Infection in sepsis and disseminated intravascular coagulation. Clin Appl Thromb Hemost. 2019;25:1-6.

1008. Holdenrieder S, Stieber P. Clinical use of circulating nucleosomes. Crit Rev Clin Lab Sci. 2009;46:1-24.

1009. Efron PA, Martins A, Minnich D, Tinsley K, Ungaro R, Bahjat FR et al. Characterization of the systemic loss of dendritic cells in murine lymph nodes during polymicrobial sepsis. J Immunol. 2004;173:3035-43.

1010. Guisset O, Dilhuydy MS, Thiebaut R, Lefevre J, Camou F, Sarrat A et al. Decrease in circulating dendritic cells predicts fatal outcome in septic shock. Intensive Care Med. 2007;33:148-52.

1011. Steinman RM, Hemmi H. Dendritic cells: translating innate to adaptive immunity. Curr Top Microbiol Immunol. 2006;311:17-58.

1012. de Jong HK, Koh GC, Bulder I, Stephan F, Wiersinga WJ, Zeerleder SS. Diabetes-independent increase of factor VII-activating protease activation in patients with Gram-negative sepsis (melioidosis). J Thromb Haemost. 2015;13:41-6.

1013. Stephan F, Hazelzet JA, Bulder I, Boermeester MA, van Till JO, van der Poll T et al. Activation of factor VII-activating protease in human inflammation: a sensor for cell death. Crit Care. 2011;15:R110.

1014. Stephan F, Aarden LA, Zeerleder S. FSAP, a new player in inflammation? Hamostaseologie. 2012;32:51-5.

1015. Accardo-Palumbo A, D'Amelio L, Pileri D, D'Arpa N, Mogavero R, Amato G et al. Reduction of plasma granzyme A correlates with severity of sepsis in burn patients. Burns. 2010;36:811-8.

1016. Schlapbach LJ, Aebi C, Hansen AG, Hirt A, Jensenius JC, Ammann RA. H-ficolin serum concentration and susceptibility to fever and neutropenia in paediatric cancer patients. Clin Exp Immunol. 2009;157:83-9.

1017. Matsushita M, Kuraya M, Hamasaki N, Tsujimura M, Shiraki H, Fujita T. Activation of the lectin complement pathway by H-ficolin (Hakata antigen). J Immunol. 2002;168:3502-6.

1018. Lacroix-Desmazes S, Bayry J, Kaveri SV, Hayon-Sonsino D, Thorenoor N, Charpentier J et al. High levels of catalytic antibodies correlate with favorable outcome in sepsis. Proc Natl Acad Sci U S A. 2005;102:4109-13.

1019. Lacroix-Desmazes S, Mallet V, Wootla B, Kaveri SV. Catalytic antibodies and severe sepsis. Discov Med. 2005;5:209-12.

1020. Baek YW, Brokat S, Padbury JF, Pinar H, Hixson DC, Lim YP. Inter-alpha inhibitor proteins in infants and decreased levels in neonatal sepsis. J Pediatr. 2003;143:11-5.

1021. Chaaban H, Singh K, Huang J, Siryaporn E, Lim YP, Padbury JF. The role of inter-alpha inhibitor proteins in the diagnosis of neonatal sepsis. J Pediatr. 2009;154:620-2.

1022. Opal SM, Lim YP, Siryaporn E, Moldawer LL, Pribble JP, Palardy JE et al. Longitudinal studies of inter-alpha inhibitor proteins in severely septic patients: a potential clinical marker and mediator of severe sepsis. Crit Care Med. 2007;35:387-92.

1023. Decembrino L, DeAmici M, De Silvestri A, Manzoni P, Paolillo P, Stronati M. Plasma lactoferrin levels in newborn preterm infants with sepsis. J Matern Fetal Neonatal Med. 2017;30:2890-3.

1024. Gutteberg TJ, Rokke O, Jorgensen T, Andersen O. Lactoferrin as an indicator of septicemia and endotoxemia in pigs. Scand J Infect Dis. 1988;20:659-66.

1025. Soderquist B, Sundqvist KG, Jones I, Holmberg H, Vikerfors T. Interleukin-6, C-reactive protein, lactoferrin and white blood cell count in patients with S. aureus septicemia. Scand J Infect Dis. 1995;27:375-80.

1026. Barber MF, Kronenberg Z, Yandell M, Elde NC. Antimicrobial functions of lactoferrin promote genetic conflicts in ancient primates and modern humans. PLoS Genet. 2016;12:e1006063.

1027. Williams JP, Thompson JP, Young SP, Gold SJ, McDonald J, Rowbotham DJ et al. Nociceptin and urotensin-II concentrations in critically ill patients with sepsis. Br J Anaesth. 2008;100:810-4.

1028. Serrano-Gomez A, Thompson JP, Lambert DG. Nociceptin/orphanin FQ in inflammation and sepsis. Br J Anaesth. 2011;106:6-12.

1029. Shimizu T, Endo Y, Tabata T, Mori T, Hanasawa K, Tsuchiya M et al. Diagnostic and predictive value of the silkworm larvae plasma test for postoperative infection following gastrointestinal surgery. Crit Care Med. 2005;33:1288-95.

1030. Tsuchiya M, Asahi N, Suzuoki F, Ashida M, Matsuura S. Detection of peptidoglycan and beta-glucan with silkworm larvae plasma test. FEMS Immunol Med Microbiol. 1996;15:129-34.

1031. Brosche T, Bertsch T, Sieber CC, Hoffmann U. Reduced plasmalogen concentration as a surrogate marker of oxidative stress in elderly septic patients. Arch Gerontol Geriatr. 2013;57:66-9.

1032. Lessig J, Fuchs B. Plasmalogens in biological systems: their role in oxidative processes in biological membranes, their contribution to pathological processes and aging and plasmalogen analysis. Curr Med Chem. 2009;16:2021-41.

1033. Aliefendioglu D, Gursoy T, Caglayan O, Aktas A, Ovali F. Can resistin be a new indicator of neonatal sepsis? Pediatr Neonatol. 2014;55:53-7.

1034. Gokmen Z, Ozkiraz S, Kulaksizoglu S, Kilicdag H, Ozel D, Ecevit A et al. Resistin--a novel feature in the diagnosis of sepsis in premature neonates. Am J Perinatol. 2013;30:513-7.

1035. Khattab AA, El-Mekkawy MS, Helwa MA, Omar ES. Utility of serum resistin in the diagnosis of neonatal sepsis and prediction of disease severity in term and late preterm infants. J Perinat Med. 2018;46:919-25.

1036. Macdonald SP, Stone SF, Neil CL, van Eeden PE, Fatovich DM, Arendts G et al. Sustained elevation of resistin, NGAL and IL-8 are associated with severe sepsis/septic shock in the emergency department. PLoS One. 2014;9:e110678.

1037. Macdonald SPJ, Bosio E, Neil C, Arendts G, Burrows S, Smart L et al. Resistin and NGAL are associated with inflammatory response, endothelial activation and clinical outcomes in sepsis. Inflamm Res. 2017;66:611-9.

1038. Sunden-Cullberg J, Nystrom T, Lee ML, Mullins GE, Tokics L, Andersson J et al. Pronounced elevation of resistin correlates with severity of disease in severe sepsis and septic shock. Crit Care Med. 2007;35:1536-42.

1039. Bokarewa M, Nagaev I, Dahlberg L, Smith U, Tarkowski A. Resistin, an adipokine with potent proinflammatory properties. J Immunol. 2005;174:5789-95.

1040. Snall J, Linner A, Uhlmann J, Siemens N, Ibold H, Janos M et al. Differential neutrophil responses to bacterial stimuli: Streptococcal strains are potent inducers of heparin-binding protein and resistin-release. Sci Rep. 2016;6:21288.

1041. Su L, Cao L, Zhou R, Jiang Z, Xiao K, Kong W et al. Identification of novel biomarkers for sepsis prognosis via urinary proteomic analysis using iTRAQ labeling and 2D-LC-MS/MS. PLoS One. 2013;8:e54237.

1042. Fang W, Goldberg ML, Pohl NM, Bi X, Tong C, Xiong B et al. Functional and physical interaction between the selenium-binding protein 1 (SBP1) and the glutathione peroxidase 1 selenoprotein. Carcinogenesis. 2010;31:1360-6.

1043. Zurek J, Kyr M, Vavrina M, Fedora M. Trefoil factor 2 expression and its significance as a predictor of severity of sepsis in children. Peptides. 2013;46:1-5.

1044. Alison MR, Chinery R, Poulsom R, Ashwood P, Longcroft JM, Wright NA. Experimental ulceration leads to sequential expression of spasmolytic polypeptide, intestinal trefoil factor, epidermal growth factor and transforming growth factor alpha mRNAs in rat stomach. J Pathol. 1995;175:405-14.

1045. Kornblit B, Hellemann D, Munthe-Fog L, Bonde J, Strom JJ, Madsen HO et al. Plasma YKL-40 and CHI3L1 in systemic inflammation and sepsis-experience from two prospective cohorts. Immunobiology. 2013;218:1227-34.

1046. Rathcke CN, Vestergaard H. YKL-40, a new inflammatory marker with relation to insulin resistance and with a role in endothelial dysfunction and atherosclerosis. Inflamm Res. 2006;55:221-7.

1047. Adrie C, Bachelet M, Vayssier-Taussat M, Russo-Marie F, Bouchaert I, Adib-Conquy M et al. Mitochondrial membrane potential and apoptosis peripheral blood monocytes in severe human sepsis. Am J Respir Crit Care Med. 2001;164:389-95.

1048. Peck-Palmer OM, Unsinger J, Chang KC, McDonough JS, Perlman H, McDunn JE et al. Modulation of the Bcl-2 family blocks sepsis-induced depletion of dendritic cells and macrophages. Shock. 2009;31:359-66.

1049. Delogu G, Famularo G, Tellan G, Marandola M, Antonucci A, Signore M et al. Lymphocyte apoptosis, caspase activation and inflammatory response in septic shock. Infection. 2008;36:485-7.

1050. Scott AM, Saleh M. The inflammatory caspases: guardians against infections and sepsis. Cell Death Differ. 2007;14:23-31.

1051. Bergmann A, Bohuon C. Decrease of serum dipeptidylpeptidase activity in severe sepsis patients: relationship to procalcitonin. Clin Chim Acta. 2002;321:123-6.

1052. Mentlein R. Dipeptidyl-peptidase IV (CD26)--role in the inactivation of regulatory peptides. Regul Pept. 1999;85:9-24.

1053. Halis H, Gunes T, Korkut S, Saraymen B, Sen A, Bastug O et al. In the diagnosis of neonatal sepsis importance of gelsolin and relationship with mortality and morbidity. Med Hypotheses. 2016;94:77-80.

1054. Wang H, Cheng B, Chen Q, Wu S, Lv C, Xie G et al. Time course of plasma gelsolin concentrations during severe sepsis in critically ill surgical patients. Crit Care. 2008;12:R106.

1055. Lee PS, Waxman AB, Cotich KL, Chung SW, Perrella MA, Stossel TP. Plasma gelsolin is a marker and therapeutic agent in animal sepsis. Crit Care Med. 2007;35:849-55.

1056. Duswald KH, Jochum M, Schramm W, Fritz H. Released granulocytic elastase: an indicator of pathobiochemical alterations in septicemia after abdominal surgery. Surgery. 1985;98:892-9.

1057. Geiger R, Sokal S, Trefz G, Siebeck M, Hoffmann H. PMN elastase and leukocyte neutral proteinase inhibitor (LNPI) from granulocytes as inflammation markers in experimental-septicemia. Adv Exp Med Biol. 1988;240:465-71.

1058. Peters KM, Koberg K, Rosendahl T, Haubeck HD. PMN elastase in bone and joint infections. Int Orthop. 1994;18:352-5.

1059. Brinkmann V, Reichard U, Goosmann C, Fauler B, Uhlemann Y, Weiss DS et al. Neutrophil extracellular traps kill bacteria. Science. 2004;303:1532-5.

1060. Borgel D, Clauser S, Bornstain C, Bieche I, Bissery A, Remones V et al. Elevated growth-arrest-specific protein 6 plasma levels in patients with severe sepsis. Crit Care Med. 2006;34:219-22.

1061. Ekman C, Linder A, Akesson P, Dahlback B. Plasma concentrations of Gas6 (growth arrest specific protein 6) and its soluble tyrosine kinase receptor sAxl in sepsis and systemic inflammatory response syndromes. Crit Care. 2010;14:R158.

1062. Hasanbasic I, Cuerquis J, Varnum B, Blostein MD. Intracellular signaling pathways involved in Gas6-Axl-mediated survival of endothelial cells. Am J Physiol Heart Circ Physiol. 2004;287:H1207-H1213.

1063. Tegtmeyer FK, Horn C, Richter A, van Wees J. Elastase alpha 1 proteinase inhibitor complex, granulocyte count, ratio of immature to total granulocyte count, and C-reactive protein in neonatal septicaemia. Eur J Pediatr. 1992;151:353-6.

1064. Fujita J, Nakamura H, Yamagishi Y, Yamaji Y, Shiotani T, Irino S. Elevation of plasma truncated elastase alpha 1-proteinase inhibitor complexes in patients with inflammatory lung diseases. Chest. 1992;102:129-34.

1065. Anand D, Ray S, Srivastava LM, Bhargava S. Evolution of serum hyaluronan and syndecan levels in prognosis of sepsis patients. Clin Biochem. 2016;49:768-76.

1066. Berg S, Brodin B, Hesselvik F, Laurent TC, Maller R. Elevated levels of plasma hyaluronan in septicaemia. Scand J Clin Lab Invest. 1988;48:727-32.

1067. Berg S, Jansson I, Hesselvik FJ, Laurent TC, Lennquist S, Walther S. Hyaluronan: relationship to hemodynamics and survival in porcine injury and sepsis. Crit Care Med. 1992;20:1315-21.

1068. Schachnik NC, Peruhype-Magalhaes V, Paula GM, Lucas F, Jr., Freitas VM, Martins-Filho OA et al. Intracellular nitric oxide assessment in whole blood leukocytes by flow cytometry: optimization and applicability to monitor patients with chronic graft nephropathy. J Immunol Methods. 2009;343:103-11.

1069. Ahn WG, Jung JS, Kwon HY, Song DK. Alteration of lysophosphatidylcholine-related metabolic parameters in the plasma of mice with experimental sepsis. Inflammation. 2017;40:537-45.

1070. Cho WH, Park T, Park YY, Huh JW, Lim CM, Koh Y et al. Clinical significance of enzymatic lysophosphatidylcholine (LPC) assay data in patients with sepsis. Eur J Clin Microbiol Infect Dis. 2012;31:1805-10.

1071. Ngwenya BZ, Yamamoto N. Activation of peritoneal macrophages by lysophosphatidylcholine. Biochim Biophys Acta. 1985;839:9-15.

1072. Serrano-Gomez S, Burgos-Angulo G, Nino-Vargas DC, Nino ME, Cardenas ME, Chacon-Valenzuela E et al. Predictive value of matrix metalloproteinases and their inhibitors for mortality in septic patients: A cohort study. J Intensive Care Med. 2017;885066617732284.

1073. Sivula M, Hastbacka J, Kuitunen A, Lassila R, Tervahartiala T, Sorsa T et al. Systemic matrix metalloproteinase-8 and tissue inhibitor of metalloproteinases-1 levels in severe sepsis-associated coagulopathy. Acta Anaesthesiol Scand. 2015;59:176-84.

1074. Vanlaere I, Libert C. Matrix metalloproteinases as drug targets in infections caused by gram-negative bacteria and in septic shock. Clin Microbiol Rev. 2009;22:224-39.

1075. Arnalich F, Garcia-Palomero E, Lopez J, Jimenez M, Madero R, Renart J et al. Predictive value of nuclear factor kappaB activity and plasma cytokine levels in patients with sepsis. Infect Immun. 2000;68:1942-5.

1076. Christman JW, Lancaster LH, Blackwell TS. Nuclear factor kappa B: a pivotal role in the systemic inflammatory response syndrome and new target for therapy. Intensive Care Med. 1998;24:1131-8.

1077. Erdem SS, Yerlikaya FH, Cicekler H, Gul M. Association between ischemia-modified albumin, homocysteine, vitamin B(12) and folic acid in patients with severe sepsis. Clin Chem Lab Med. 2012;50:1417-21.

1078. Yerlikaya FH, Kurban S, Mehmetoglu I, Annagur A, Altunhan H, Erbay E et al. Serum ischemia-modified albumin levels at diagnosis and during treatment of late-onset neonatal sepsis. J Matern Fetal Neonatal Med. 2014;27:1723-7.

1079. Yin M, Liu X, Chen X, Li C, Qin W, Han H et al. Ischemia-modified albumin is a predictor of short-term mortality in patients with severe sepsis. J Crit Care. 2017;37:7-12.

1080. Roy D, Quiles J, Gaze DC, Collinson P, Kaski JC, Baxter GF. Role of reactive oxygen species on the formation of the novel diagnostic marker ischaemia modified albumin. Heart. 2006;92:113-4.

1081. Claus RA, Bunck AC, Bockmeyer CL, Brunkhorst FM, Losche W, Kinscherf R et al. Role of increased sphingomyelinase activity in apoptosis and organ failure of patients with severe sepsis. FASEB J. 2005;19:1719-21.

1082. Levade T, Jaffrezou JP. Signalling sphingomyelinases: which, where, how and why? Biochim Biophys Acta. 1999;1438:1-17.

1083. Mitsuhashi H, Ikeuchi H, Yamashita S, Kuroiwa T, Kaneko Y, Hiromura K et al. Increased levels of serum sulfite in patients with acute pneumonia. Shock. 2004;21:99-102.

1084. Mitsuhashi H, Nojima Y, Tanaka T, Ueki K, Maezawa A, Yano S et al. Sulfite is released by human neutrophils in response to stimulation with lipopolysaccharide. J Leukoc Biol. 1998;64:595-9.

1085. Hull MA, Jones BA, Zurakowski D, Raphael B, Lo C, Jaksic T et al. Low serum citrulline concentration correlates with catheter-related bloodstream infections in children with intestinal failure. JPEN J Parenter Enteral Nutr. 2011;35:181-7.

1086. Shen LJ, Guan YY, Wu XP, Wang Q, Wang L, Xiao T et al. Serum citrulline as a diagnostic marker of sepsis-induced intestinal dysfunction. Clin Res Hepatol Gastroenterol. 2015;39:230-6.

1087. Steelman SM, Johnson P, Jackson A, Schulze J, Chowdhary BP. Serum metabolomics identifies citrulline as a predictor of adverse outcomes in an equine model of gut-derived sepsis. Physiol Genomics. 2014;46:339-47.

1088. Ware LB, Magarik JA, Wickersham N, Cunningham G, Rice TW, Christman BW et al. Low plasma citrulline levels are associated with acute respiratory distress syndrome in patients with severe sepsis. Crit Care. 2013;17:R10.

1089. Erez A, Nagamani SC, Shchelochkov OA, Premkumar MH, Campeau PM, Chen Y et al. Requirement of argininosuccinate lyase for systemic nitric oxide production. Nat Med. 2011;17:1619-26.

1090. Kuroda K, Wake H, Mori S, Hinotsu S, Nishibori M, Morimatsu H. Decrease in histidine-rich glycoprotein as a novel biomarker to predict sepsis among systemic inflammatory response syndrome. Crit Care Med. 2018;46:570-6.

1091. Wake H, Mori S, Liu K, Morioka Y, Teshigawara K, Sakaguchi M et al. Histidine-rich glycoprotein prevents septic lethality through regulation of immunothrombosis and inflammation. EBioMedicine. 2016;9:180-94.

1092. Decembrino L, De Amici M, Pozzi M, De Silvestri A, Stronati M. Serum calprotectin: A potential biomarker for neonatal sepsis. J Immunol Res. 2015;2015:147973.

1093. Huang L, Li J, Han Y, Zhao S, Zheng Y, Sui F et al. Serum calprotectin expression as a diagnostic marker for sepsis in postoperative intensive care unit patients. J Interferon Cytokine Res. 2016;36:607-16.

1094. Jonsson N, Nilsen T, Gille-Johnson P, Bell M, Martling CR, Larsson A et al. Calprotectin as an early biomarker of bacterial infections in critically ill patients: an exploratory cohort assessment. Crit Care Resusc. 2017;19:205-13.

1095. Simm M, Soderberg E, Larsson A, Castegren M, Nilsen T, Eriksson M et al. Performance of plasma calprotectin as a biomarker of early sepsis: a pilot study. Biomark Med. 2016;10:811-8.

1096. Terrin G, Passariello A, Manguso F, Salvia G, Rapacciuolo L, Messina F et al. Serum calprotectin: an antimicrobial peptide as a new marker for the diagnosis of sepsis in very low birth weight newborns. Clin Dev Immunol. 2011;2011:291085.

1097. Bartakova E, Stefan M, Stranikova A, Pospisilova L, Arientova S, Beran O et al. Calprotectin and calgranulin C serum levels in bacterial sepsis. Diagn Microbiol Infect Dis. 2019;93:219-26.

1098. Hetland G, Talgo GJ, Fagerhol MK. Chemotaxins C5a and fMLP induce release of calprotectin (leucocyte L1 protein) from polymorphonuclear cells in vitro. Mol Pathol. 1998;51:143-8.

1099. Jiang ZM, Yang QH, Zhu CQ. UCP2 in early diagnosis and prognosis of sepsis. Eur Rev Med Pharmacol Sci. 2017;21:549-53.

1100. Jaburek M, Varecha M, Gimeno RE, Dembski M, Jezek P, Zhang M et al. Transport function and regulation of mitochondrial uncoupling proteins 2 and 3. J Biol Chem. 1999;274:26003-7.

1101. Chew M, Rahman M, Ihrman L, Erson A, Zhang S, Thorlacius H. Soluble CD40L (CD154) is increased in patients with shock. Inflamm Res. 2010;59:979-82.

1102. Lopez S, Prats N, Marco AJ. Expression of E-selectin, P-selectin, and intercellular adhesion molecule-1 during experimental murine listeriosis. Am J Pathol. 1999;155:1391-7.

1103. Mosad E, Elsayh KI, Eltayeb AA. Tissue factor pathway inhibitor and P-selectin as markers of sepsis-induced non-overt disseminated intravascular coagulopathy. Clin Appl Thromb Hemost. 2011;17:80-7.

1104. Schrijver IT, Kemperman H, Roest M, Kesecioglu J, de Lange DW. Soluble P-selectin as a biomarker for infection and survival in patients with a systemic inflammatory response syndrome on the intensive care unit. Biomark Insights. 2017;12:1177271916684823.

1105. Simons RK, Hoyt DB, Winchell RJ, Rose RM, Holbrook T. Elevated selectin levels after severe trauma: a marker for sepsis and organ failure and a potential target for immunomodulatory therapy. J Trauma. 1996;41:653-62.

1106. Vassiliou AG, Mastora Z, Orfanos SE, Jahaj E, Maniatis NA, Koutsoukou A et al. Elevated biomarkers of endothelial dysfunction/activation at ICU admission are associated with sepsis development. Cytokine. 2014;69:240-7.

1107. Duman A, Turkdogan KA, Avcil M, Yenisey C, Ture M, Akoz A et al. The predictive value of the inflammatory markers P-selectin and MCP1 in determining the length of stay and 30-day survival in the differentiation of sepsis patients. J Pak Med Assoc. 2018;68:1321-6.

1108. de Stoppelaar SF, Van't Veer C, Roelofs JJ, Claushuis TA, de Boer OJ, Tanck MW et al. Platelet and endothelial cell P-selectin are required for host defense against Klebsiella pneumoniae-induced pneumosepsis. J Thromb Haemost. 2015;13:1128-38.

1109. Maccarrone M, De Petrocellis L, Bari M, Fezza F, Salvati S, Di Marzo V et al. Lipopolysaccharide downregulates fatty acid amide hydrolase expression and increases anandamide levels in human peripheral lymphocytes. Arch Biochem Biophys. 2001;393:321-8.

1110. Vercelli CA, Aisemberg J, Billi S, Cervini M, Ribeiro ML, Farina M et al. Anandamide regulates lipopolysaccharide-induced nitric oxide synthesis and tissue damage in the murine uterus. Reprod Biomed Online. 2009;18:824-31.

1111. Wang Y, Liu Y, Ito Y, Hashiguchi T, Kitajima I, Yamakuchi M et al. Simultaneous measurement of anandamide and 2-arachidonoylglycerol by polymyxin B-selective adsorption and subsequent high-performance liquid chromatography analysis: increase in endogenous cannabinoids in the sera of patients with endotoxic shock. Anal Biochem. 2001;294:73-82.

1112. Szafran B, Borazjani A, Lee JH, Ross MK, Kaplan BL. Lipopolysaccharide suppresses carboxylesterase 2g activity and 2-arachidonoylglycerol hydrolysis: A possible mechanism to regulate inflammation. Prostaglandins Other Lipid Mediat. 2015;121:199-206.

1113. Crouser ED, Julian MW, Huff JE, Struck J, Cook CH. Carbamoyl phosphate synthase-1: a marker of mitochondrial damage and depletion in the liver during sepsis. Crit Care Med. 2006;34:2439-46.

1114. Struck J, Uhlein M, Morgenthaler NG, Furst W, Hoflich C, Bahrami S et al. Release of the mitochondrial enzyme carbamoyl phosphate synthase under septic conditions. Shock. 2005;23:533-8.

1115. Schuerholz T, Leuwer M, Cobas-Meyer M, Vangerow B, Kube F, Kirschfink M et al. Terminal complement complex in septic shock with capillary leakage: marker of complement activation? Eur J Anaesthesiol. 2005;22:541-7.

1116. Hataya Y, Akamizu T, Hosoda H, Kanamoto N, Moriyama K, Kangawa K et al. Alterations of plasma ghrelin levels in rats with lipopolysaccharide-induced wasting syndrome and effects of ghrelin treatment on the syndrome. Endocrinology. 2003;144:5365-71.

1117. Zhou M, Aziz M, Ochani M, Yang WL, Sharma A, Wang P. The protective role of human ghrelin in sepsis: Restoration of CD4 T cell proliferation. PLoS One. 2018;13:e0201139.

1118. Bee N, White R, Petros AJ. Hydrogen sulfide in exhaled gases from ventilated septic neonates and children: A preliminary report. Pediatr Crit Care Med. 2017;18:e327-e332.

1119. Li L, Bhatia M, Zhu YZ, Zhu YC, Ramnath RD, Wang ZJ et al. Hydrogen sulfide is a novel mediator of lipopolysaccharide-induced inflammation in the mouse. FASEB J. 2005;19:1196-8.

1120. Whiteman M, Winyard PG. Hydrogen sulfide and inflammation: the good, the bad, the ugly and the promising. Expert Rev Clin Pharmacol. 2011;4:13-32.

1121. Jones JW, Jr., Su S, Jones MB, Heniford BT, McIntyre K, Granger DK. Serum lysozyme activity can differentiate infection from rejection in organ transplant recipients. J Surg Res. 1999;84:134-7.

1122. Gotes J, Kasian K, Jacobs H, Cheng ZQ, Mink SN. Lysozyme, a mediator of sepsis that deposits in the systemic vasculature and kidney as a possible mechanism of acute organ dysfunction. Shock. 2014;41:256-65.

1123. Piliponsky AM, Chen CC, Nishimura T, Metz M, Rios EJ, Dobner PR et al. Neurotensin increases mortality and mast cells reduce neurotensin levels in a mouse model of sepsis. Nat Med. 2008;14:392-8.

1124. Tyler-McMahon BM, Boules M, Richelson E. Neurotensin: peptide for the next millennium. Regul Pept. 2000;93:125-36.

1125. Yano K, Liaw PC, Mullington JM, Shih SC, Okada H, Bodyak N et al. Vascular endothelial growth factor is an important determinant of sepsis morbidity and mortality. J Exp Med. 2006;203:1447-58.

1126. Yano K, Okada Y, Beldi G, Shih SC, Bodyak N, Okada H et al. Elevated levels of placental growth factor represent an adaptive host response in sepsis. J Exp Med. 2008;205:2623-31.

1127. Costa NA, Gut AL, Pimentel JA, Cozzolino SM, Azevedo PS, Fernandes AA et al. Erythrocyte selenium concentration predicts intensive care unit and hospital mortality in patients with septic shock: a prospective observational study. Crit Care. 2014;18:R92.

1128. Sakr Y, Reinhart K, Bloos F, Marx G, Russwurm S, Bauer M et al. Time course and relationship between plasma selenium concentrations, systemic inflammatory response, sepsis, and multiorgan failure. Br J Anaesth. 2007;98:775-84.

1129. Mertens K, Lowes DA, Webster NR, Talib J, Hall L, Davies MJ et al. Low zinc and selenium concentrations in sepsis are associated with oxidative damage and inflammation. Br J Anaesth. 2015;114:990-9.

1130. Kothari N, Keshari RS, Bogra J, Kohli M, Abbas H, Malik A et al. Increased myeloperoxidase enzyme activity in plasma is an indicator of inflammation and onset of sepsis. J Crit Care. 2011;26:435-7.

1131. Rasooli A, Ghafari E, Saedi H, Miri S. Expression changes of CD177 and MPO as novel biomarkers in lung tissue of CLP model rats. Turk J Med Sci. 2018;48:1321.

1132. Reber LL, Gillis CM, Starkl P, Jonsson F, Sibilano R, Marichal T et al. Neutrophil myeloperoxidase diminishes the toxic effects and mortality induced by lipopolysaccharide. J Exp Med. 2017;214:1249-58.

1133. Yuan W, Zhang W, Yang X, Zhou L, Hanghua Z, Xu K. Clinical significance and prognosis of serum tenascin-C in patients with sepsis. BMC Anesthesiol. 2018;18:170.

1134. Li X, Zhu Z, Zhou T, Cao X, Lu T, He J et al. Predictive value of combined serum FGF21 and free T3 for survival in septic patients. Clin Chim Acta. 2019;494:31-7.

1135. Yu Y, He J, Li S, Song L, Guo X, Yao W et al. Fibroblast growth factor 21 (FGF21) inhibits macrophage-mediated inflammation by activating Nrf2 and suppressing the NF-kappaB signaling pathway. Int Immunopharmacol. 2016;38:144-52.

1136. Kim WY, Lee SH, Kim DY, Ryu HJ, Chon GR, Park YY et al. Serum developmental endothelial locus-1 is associated with severity of sepsis in animals and humans. Sci Rep. 2019;9:13005.

1137. Choi EY, Chavakis E, Czabanka MA, Langer HF, Fraemohs L, Economopoulou M et al. Del-1, an endogenous leukocyte-endothelial adhesion inhibitor, limits inflammatory cell recruitment. Science. 2008;322:1101-4.

1138. Gao X, Liu Y, Xu F, Lin S, Song Z, Duan J et al. Assessment of apoptosis inhibitor of macrophage/CD5L as a biomarker to predict mortality in the critically ill with sepsis. Chest. 2019;156:696-705.

1139. Haruta I, Kato Y, Hashimoto E, Minjares C, Kennedy S, Uto H et al. Association of AIM, a novel apoptosis inhibitory factor, with hepatitis via supporting macrophage survival and enhancing phagocytotic function of macrophages. J Biol Chem. 2001;276:22910-4.

1140. Chung KP, Chen GY, Chuang TY, Huang YT, Chang HT, Chen YF et al. Increased plasma acetylcarnitine in sepsis is associated with multiple organ dysfunction and mortality: A multicenter cohort study. Crit Care Med. 2019;47:210-8.

1141. Yagmur E, Otto S, Koek GH, Weiskirchen R, Trautwein C, Koch A et al. Decreased CTRP3 plasma concentrations are associated with sepsis and predict mortality in critically ill patients. Diagnostics (Basel). 2019;9:E63.

1142. Petersen PS, Wolf RM, Lei X, Peterson JM, Wong GW. Immunomodulatory roles of CTRP3 in endotoxemia and metabolic stress. Physiol Rep. 2016;4:e12735.

1143. Hildebrand D, Decker SO, Koch C, Schmitt FCF, Ruhrmann S, Schneck E et al. Host-derived delta-like canonical Notch ligand 1 as a novel diagnostic biomarker for bacterial sepsis - results from a combinational secondary analysis. Front Cell Infect Microbiol. 2019;9:267.

1144. Kovall RA, Gebelein B, Sprinzak D, Kopan R. The canonical Notch signaling pathway: Structural and biochemical insights into shape, sugar, and force. Dev Cell. 2017;41:228-41.

1145. Whiting PF, Rutjes AW, Westwood ME, Mallett S, Deeks JJ, Reitsma JB et al. QUADAS-2: a revised tool for the quality assessment of diagnostic accuracy studies. Ann Intern Med. 2011;155:529-36.

1146. Rannikko J, Seiskari T, Huttunen R, Tarkiainen I, Jylhava J, Hurme M et al. Plasma cell-free DNA and qSOFA score predict 7-day mortality in 481 emergency department bacteraemia patients. J Intern Med. 2018;284:418-26.
